# Chronic alcohol consumption from adolescence-to-adulthood in mice - hypothalamic gene expression changes in the dilated cardiomyopathy signaling pathway

**DOI:** 10.1186/1471-2202-15-61

**Published:** 2014-05-09

**Authors:** Hong Zou, Ke Wang, Yang Gao, Huaiguang Song, Qinglian Xie, Meilei Jin, Guoping Zhao, Huasheng Xiao, Lei Yu

**Affiliations:** 1 Shanghai Institutes for Biological Sciences, Chinese Academy of Sciences, Shanghai, China; 2 State Key Laboratory of Genetic Engineering, Department of Microbiology, School of Life Sciences and Institute of Biomedical Sciences, Fudan University, Shanghai, China; 3 Shanghai-MOST Key Laboratory of Disease and Health Genomics, Chinese National Human Genome Center at Shanghai, National Engineering Research Center for Biochip at Shanghai, Shanghai, China; 4 Shu Guang Hospital affiliated with the Shanghai Traditional Medicine University, Shanghai, China; 5 Department of Microbiology and Li Ka Shing Institute of Health Sciences, The Chinese University of Hong Kong, Prince of Wales Hospital, Hong Kong, SAR, China; 6 Department of Genetics & Center of Alcohol Studies, Rutgers University, 607 Allison Road, Piscataway, New Jersey 08854, USA

**Keywords:** Chronic alcohol drinking, Self-administration, Adolescence, Mice, Gene expression, Hypothalamus

## Abstract

**Background:**

Adolescence is a developmental stage vulnerable to alcohol drinking-related problems and the onset of alcoholism. Hypothalamus is a key brain region for food and water intake regulation, and is one of the alcohol-sensitive brain regions. However, it is not known what would be the alcohol effect on hypothalamus following adolescent alcohol intake, chronically over the adolescent development, at moderate levels.

**Results:**

We employed a paradigm of chronic moderate alcohol intake from adolescence-to-adulthood in mice, and analyzed the alcohol effect on both behavioral and hypothalamic gene expression changes. A total of 751 genes were found and subjected to pathway analysis. The dilated cardiomyopathy (DCM) pathway was identified. The changes of ten genes under this pathway were further verified using RT-PCR. Chronic alcohol consumption during adolescence, even at moderate levels, led to a decrease of motor activity in mice, and also a concerted down regulation of signaling pathway initiating factor (SPIF) genes in the DCM signaling pathway, including β1-adrenergic receptor (Adrb1), Gs protein (Gnas), adenylyl cyclase 1 (Adcy1), and dihydropyridine receptor/L-type calcium channel (Cacna1d).

**Conclusions:**

These findings suggest that adolescent alcohol intake may trigger gene expression changes in the CNS that parallel those found in the dilated cardiomyopathy signaling pathway. If such effects also take place in humans, our findings would serve as a warning against alcohol intake in youth, such as by teens and/or college students.

## Background

Adolescence is a critical transitional stage during which youth mature into adulthood. This developmental stage is characterized by a continued brain remodeling, a process that is sensitive to the disrupting effects of alcohol [1,2]. Epidemiological studies indicate that adolescence is a developmental stage particularly vulnerable to alcohol drinking-related problems and the onset of alcoholism [3-7].

The brain is one of the major target organs of alcohol. Excessive alcohol exposure can lead to structural and functional changes of the brain [8-11], resulting in malfunctioning of the CNS activity, as well as deleterious influence of bodily function [12-14].

Hypothalamus is one of the alcohol-sensitive brain regions [15]. Being a key brain region for food and water intake regulation [16-18] and part of the reward system [19-21], the hypothalamus can modulate alcohol consumption and is involved in the development of alcoholism. It has been shown that stimulation of lateral hypothalamic regions could trigger alcohol consumption [22,23]. Neuropeptides that are found in the hypothalamus could stimulate alcohol intake [24-26].

Conversely, alcohol can also affect hypothalamic activities. Alcohol is known to activate the HPA axis [27-29]. It is likely that by modifying the activity of the HPA axis, alcohol will influence, and possibly compromise, the ability of the body to maintain or restore homeostasis and to coordinate appropriate behavioral responses in response to stressors [30]. In alcohol-dependent subjects, dopamine D3 receptor (DRD3) binding in the hypothalamus was increased [31]. Alcohol alters genes expression of LHRH secretory pathway in the hypothalamus of prepubertal female rats, resulting in suppressed LHRH secretion and delayed puberty [32,33]. The expression of hypothalamic proopiomelanocortin (POMC) gene was reduced by prenatal exposure to alcohol in rats [34], and the POMC expression reduction persisted into adulthood by postnatal alcohol feeding in mice [35]. The expression of Fos-related immediate early genes was altered following chronic alcohol administration in mice and rats [15,36,37].

Aside from alcohol, other substances of abuse potential have been shown to influence gene activity in the hypothalamus. Thus, cocaine increased POMC gene expression in rat hypothalamus [35], and escalated cocaine intake correlated with a number of genes in the lateral hypothalamic regions [38]. Nicotine exposure was shown to affect brain development, corresponding to abnormal hypothalamic gene expression of appetite regulators such as down-regulation of NPY and POMC in the arcuate nucleus of the hypothalamus [39].

Hypothalamus also plays a role in autonomic modulation of cardiovascular system. Sympathetic nervous system can also be activated by alcohol, and chronic exposure of the heart to elevated catecholamine levels released from sympathetic nerve terminals may result in pathological changes of the heart [40,41].

Studies of the effect of chronic alcohol exposure on the hypothalamus have been limited, particularly at the level of gene expression changes. Specifically, it is not known what would be the alcohol effect on hypothalamus following adolescent alcohol intake, chronically over the adolescent development, at moderate levels. In the present study, we employed a paradigm of chronic moderate alcohol intake from adolescence-to-adulthood in mice [42], and report here the alcohol effect on both behavioral and hypothalamic gene expression changes.

## Results

### Chronic alcohol consumption

**Figure 1:**
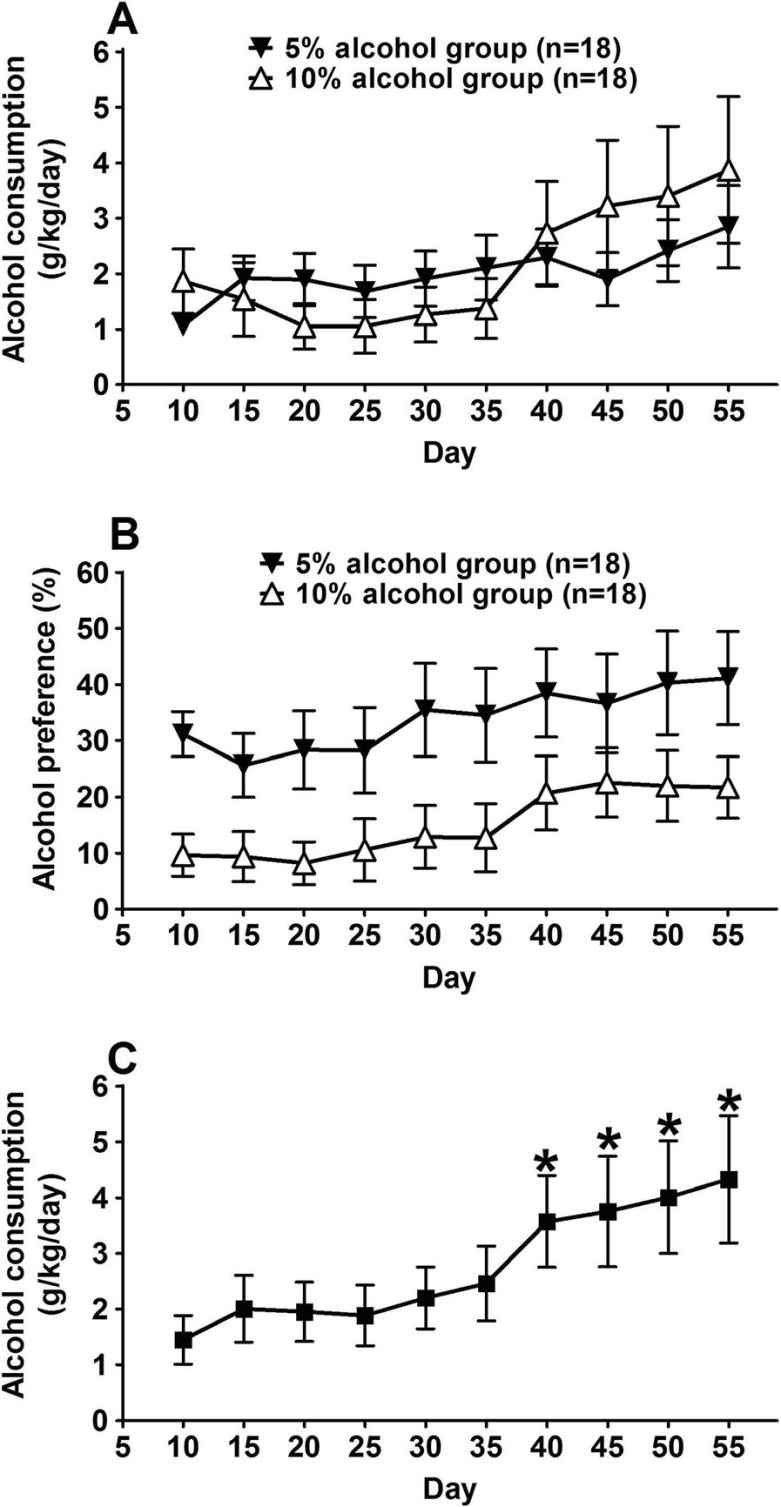
**Chronic self-administration of alcohol in mice during adolescence. (A)** Adolescent mice (3 weeks old) had free access to water and alcohol solutions. Two alcohol groups were used; one group had 5% alcohol (filled triangles), and the other had 10% alcohol (open triangles). Data are shown for daily alcohol consumption (grams of alcohol per kilogram mouse body weight per day, average over 5 days). Data are expressed as mean ± S.E.M. (n = 18 for each group). There was no statistically significant difference between the two groups at any time point (p > 0.05, two-way ANOVA with Bonferroni post-test). **(B)** Alcohol preference over water (% v/v), from the same mouse alcohol groups as in **(A)**, showing that mice from the 5% alcohol group consumed higher volume of alcohol solution than those from the 10% alcohol group. The average amounts of alcohol consumed by the 5% vs. 10% groups were not significantly different. **(C)** Alcohol consumption data of the mice used for microarray gene expression study. Mice used for the gene expression study were selected from the 5% (n = 9) and 10% (n = 10) alcohol group, respectively. Data are expressed as mean ± S.E.M. (n = 19). A steady increase of alcohol consumption was present, as the alcohol consumption data from later time points (day 40 and later) are significantly different from the data at the beginning (p < 0.05, Repeated measures ANOVA with post test of Dunnett's multiple comparison test).

We used a two-bottle free-choice paradigm to measure alcohol drinking [42], and studied effect of chronic alcohol consumption during adolescent development. Male adolescent mice (3 weeks of age) were divided into two alcohol groups, one with free access to 5% alcohol, and the other to 10% alcohol, so as to determine if there was a difference in alcohol consumption behavior due to difference in alcohol concentration. In addition, a water-only control group (each mouse had 2 bottles, both filled with water) was used. Figure 1A showed the results of daily alcohol consumption (grams of alcohol per kilogram mouse body weight per day, average over 5 days) for the 5% alcohol group (n = 18) and the 10% alcohol group (n = 18). Both groups of mice consumed similar amounts of alcohol, and there was no statistically significant difference (F(1,306) = 0.1292, p = 0.7215, two-way ANOVA with Bonferroni post-test). Figure 1B plotted alcohol preference (alcohol volume over water) from the same mouse alcohol groups, showing that mice from the 5% alcohol group consumed higher volume of alcohol solution than those from the 10% alcohol group. Since the 10% alcohol solution contained higher amount of alcohol per unit volume than the 5% alcohol solution, we calculated the absolute amounts of alcohol consumption by mice from both the 5% alcohol group and the 10% alcohol group, and found that there was no significant difference for the absolute amount of alcohol consumed between the 5% vs. 10% alcohol groups.

Because there was no significant difference for the daily alcohol consumption between the 5% and the 10% alcohol groups, we combined mice from the 5% and 10% alcohol groups, to analyze the overall pattern of alcohol consumption (Figure 1C). Over the course of adolescent alcohol exposure, a trend of increased alcohol consumption was observed, with alcohol consumption data from later time points (day 40 and later) significantly different from those in the earlier time points (p < 0.05, repeated measures ANOVA with post test of Dunnett's multiple comparison test). Average daily alcohol consumption over the duration of the entire procedure (50 days) was 2.8 g/kg/day, and for the last 5 days (day 50 – 55) it was 4.3 g/kg/day. These amounts of daily alcohol consumption represent a moderate level of alcohol consumption (see Discussion).

**Figure 2:**
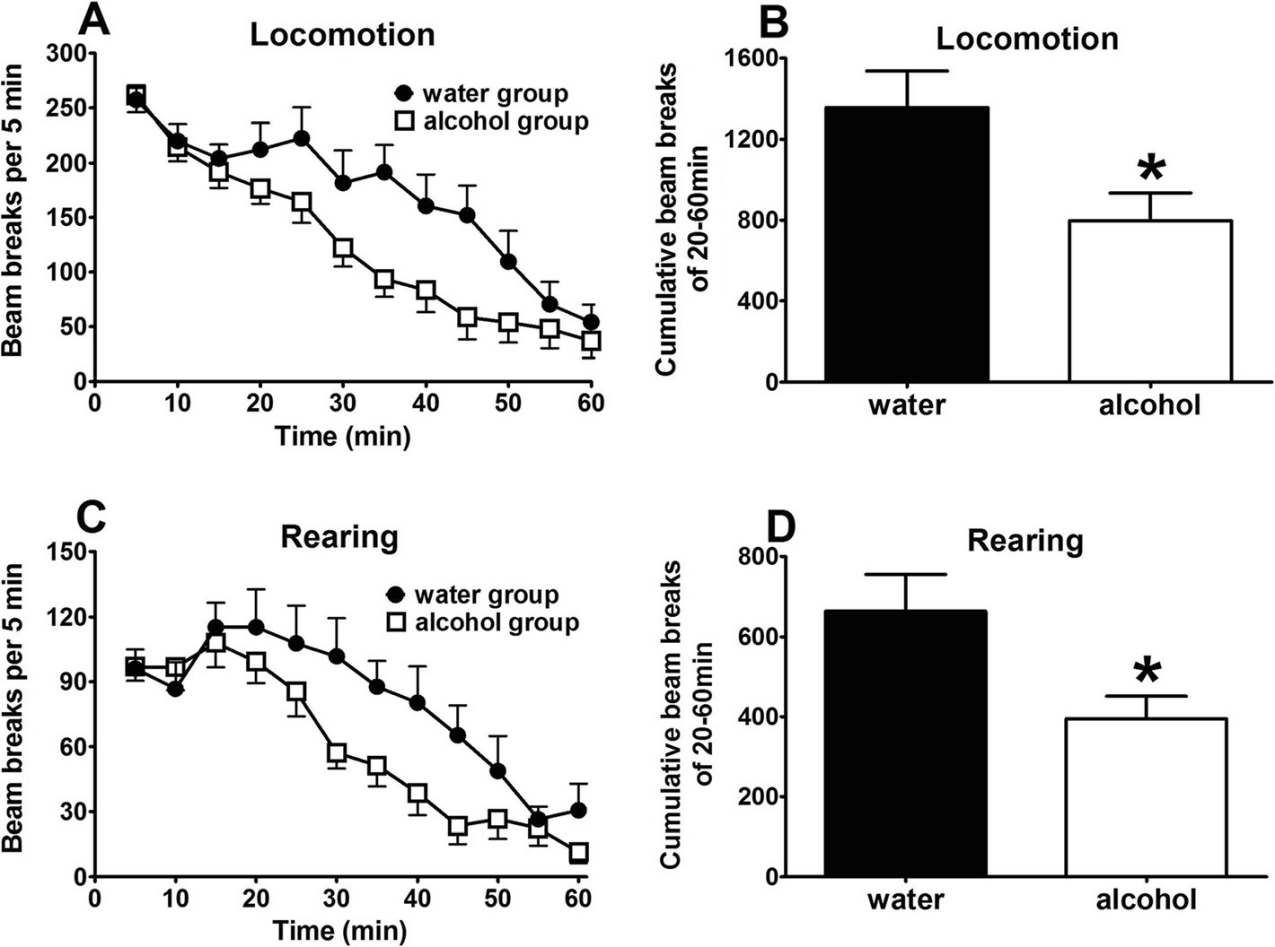
**Chronic alcohol consumption leads to a decrease of motor activity in mice.** Upper panels **(A** and **B)**: locomotion; lower panels **(C** and **D)**: rearing. Time course of locomotion **(A)** and rearing **(C)** are shown as beam breaks per 5 min. Cumulative locomotion **(B)** and rearing **(D)** for the entire period (0–60 min) are shown. Data are expressed as mean ± S.E.M. (n = 19 for the alcohol group, n = 8 for the water-only control group). *, significant difference compared with the water-only control group (p < 0.05, unpaired t-test).

Evaluation of motor activities indicated that chronic alcohol mice displayed accelerated acclimation for motor activities (Figure 2). This is consistent with reported alcohol exposure-induced locomotor activity changes [43-45].

### Gene expression changes after chronic alcohol exposure

For brain tissue processing for gene expression analysis, 19 mice with 7 weeks alcohol exposure were selected and grouped into alcohol sample groups (n = 1–3 per sample group). Samples A1-A3 had mice with relatively less alcohol consumed (0.50 ± 0.38 g/kg/day); samples A4-A9 had mice with relatively more alcohol consumed (4.80 ± 0.36 g/kg/day); these were significantly different (p < 0.05, Unpaired t test with Welch's correction). The rationale for such an approach of sample pooling was to cover the spectrum of mouse alcohol consumption behavior, so that the underlying gene expression patterns may be discernible. For water-only controls, nine mice were selected randomly, and grouped into water sample groups (n = 3 per sample group), designated samples C1-C3.

**Figure 3:**
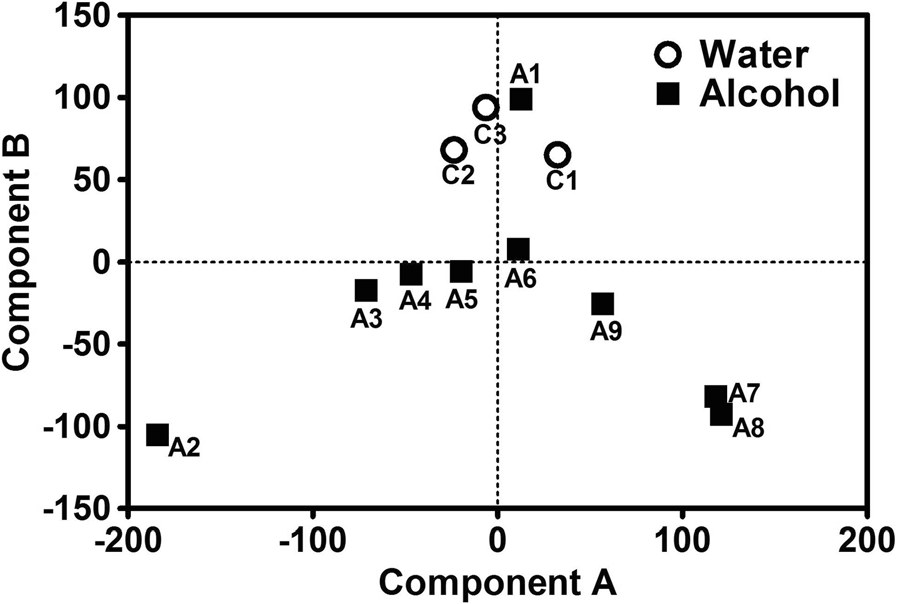
**Principal component analysis (PCA) plot of microarray gene expression study.** Twelve samples were used: C1 – C3 for water control mice (open circles), and A1 – A9 for alcohol consumption mice (filled squares). All probe sets that passed the filters for high-quality array data (see Methods) were used for the analysis. Component A is plotted on the horizontal axis and component B is plotted on the vertical axis.

RNA samples extracted from hypothalamus were used in gene expression analysis, using the Agilent mouse whole genome microarray chip set. Data from all probe sets (after passing through the filters for high-quality array data, see Methods) were used in principal component analysis (PCA). Results of PCA were shown in Figure 3, showing different distribution patterns of alcohol mice vs. water mice. For component A from PCA, both water controls and chronic alcohol mice showed results centered around the 0 axis. However, while the component A results from water samples were tightly grouped around the 0 axis, that from chronic alcohol mice were spread more widely, indicating more heterogeneity in gene expression represented in component A. For component B, results from water controls were tightly grouped above positive 50 level, while most (except sample A1) alcohol samples were near (sample A6) or in the negative value range. The distribution pattern highlighted the fact that chronic alcohol consumption mice had very different gene expression patterns compared to the water controls, indicating that chronic alcohol exposure during adolescent development lead to altered gene expression in the hypothalamus.

**Figure 4:**
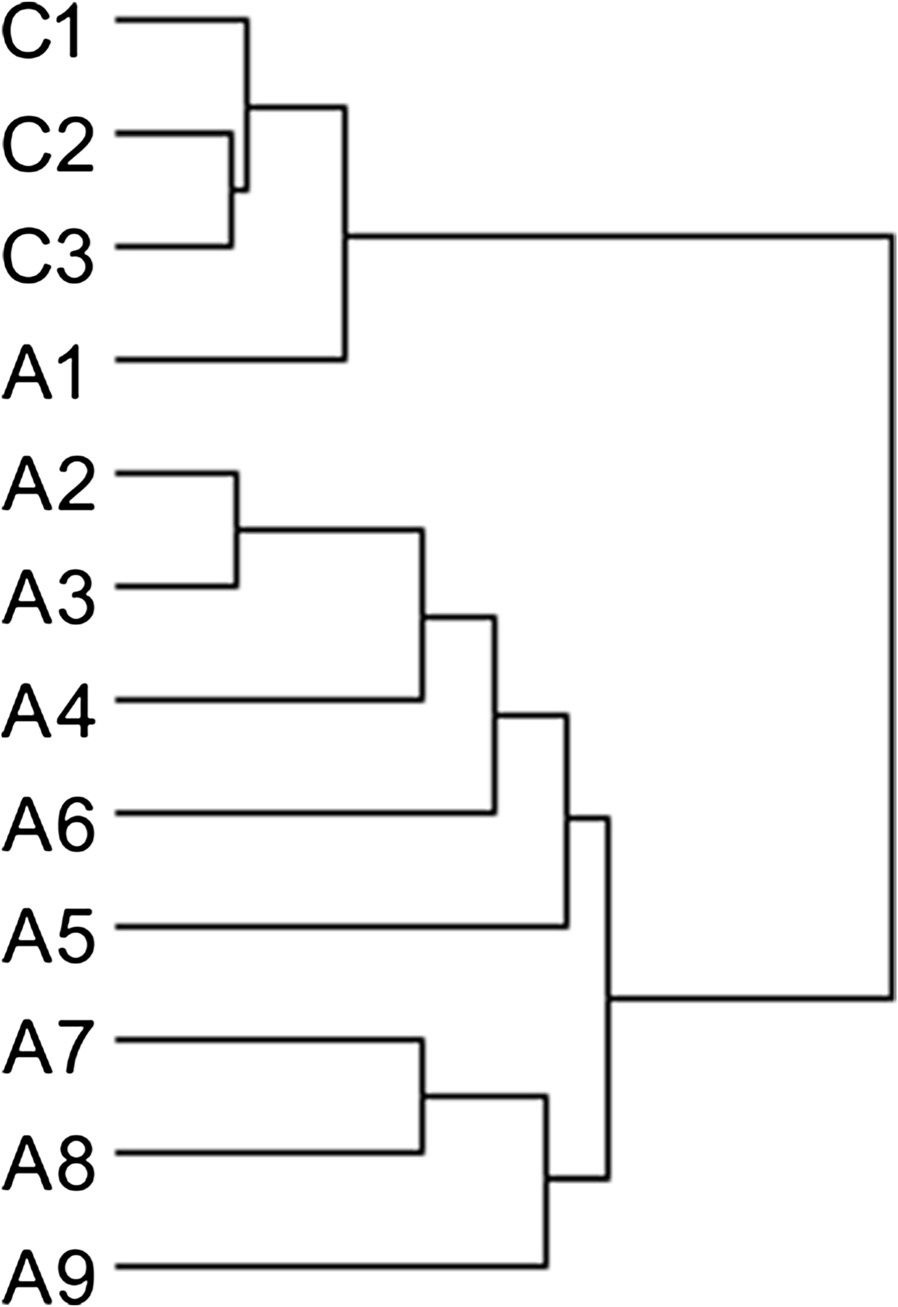
**Clustering display of differentially expressed genes using the unsupervised hierarchical clustering method.** C1-C3 represent water-only control samples; A1-A9 are for chronic alcohol samples.

Significant differences in gene expression were observed between chronic alcohol exposure and water controls, with 1900 regulated probes representing 1819 genes (890 up-regulated and 929 down-regulated, chronic alcohol relative to water controls) using the *t*-test at the *p* < 0.05 (Additional file 1: Table S1 and Additional file 2: Figure S1). Unsupervised hierarchical clustering analysis based on these differentially expressed genes also showed distinct clustering of the alcohol hypothalamus samples compared with control hypothalamus samples (Figure 4 and Additional file 2: Figure S1).

### Pathway analysis of differentially expressed genes

Of the 1819 differentially expressed genes, 340 genes showed less than 0.83 fold change (representing 20% down-regulation of chronic alcohol exposure relative to water control) and 411 genes showed more than 1.2 fold change (representing 20% up-regulation of chronic alcohol exposure relative to water control). These 751 genes were chosen for pathway analysis, and several pathways were identified (Additional file 3: Table S4). One pathway showed *p* < 0.01 — the *dilated cardiomyopathy* pathway, from the pathway enrichment analysis by both DAVID Functional Classification Analysis and Web-based Gene Set Analysis, and was chosen for further studies.

### Validation of microarray results by real-time PCR

**Figure 5:**
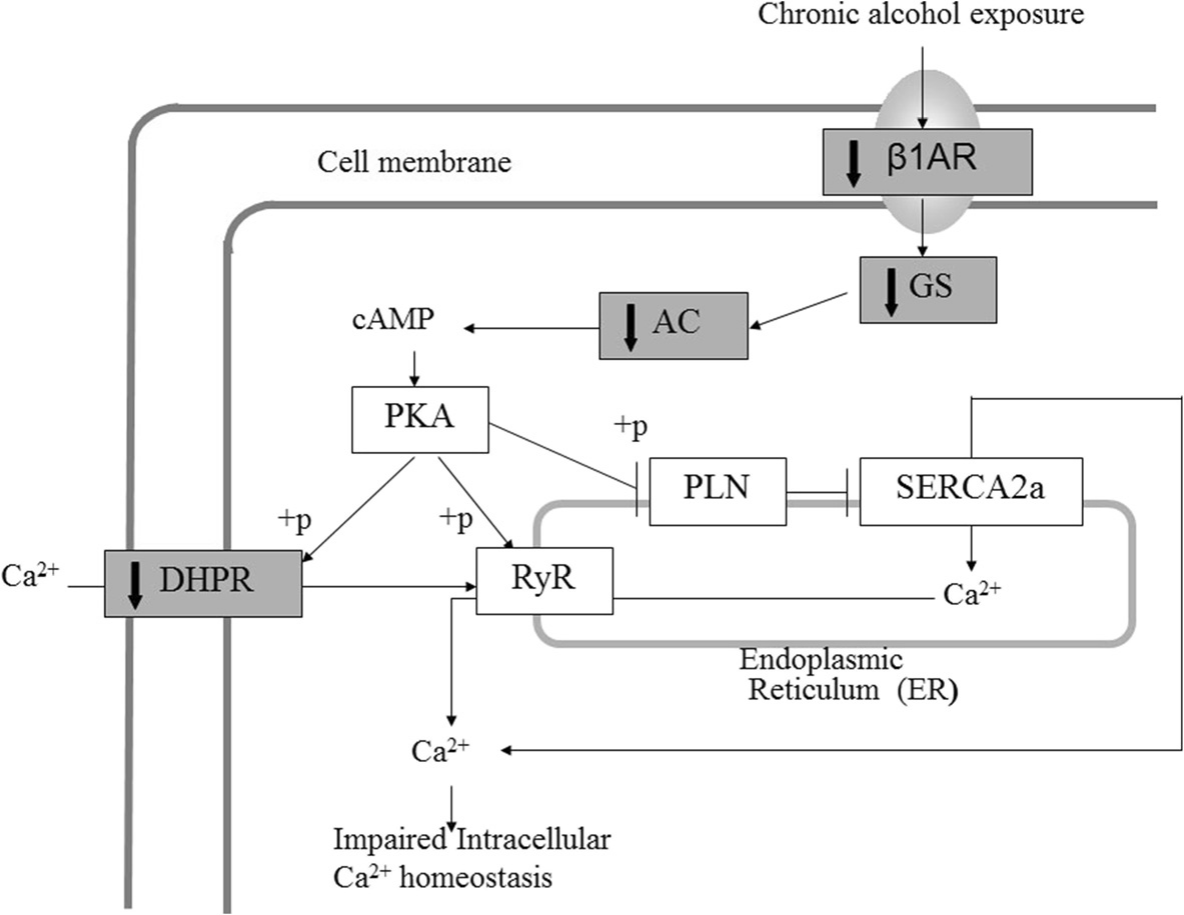
**Hypothesis of adolescent chronic alcohol consumption effect on SPIF gene down regulation in the dilated cardiomyopathy signaling pathway.** Diagram was adapted from KEGG pathway (http://www.genome.jp/kegg/) [92] for dilated cardiomyopathy (DCM), showing the β1AR-DHPR-Ca^2+^ branch of the DCM pathway. Adolescent chronic alcohol consumption is hypothesized to result in the down regulation of SPIF genes (marked with grey color and solid down arrows), including β1-adrenergic receptor (Adrb1), Gs protein (Gnas), adenylyl cyclase 1 (Adcy1), and dihydropyridine receptor/L-type calcium channel (Cacna1d). The coordinated down regulation of these SPIF genes leads to impaired intracellular Ca^2+^ homeostasis at the cellular level.

To provide independent validation of the identified gene pathway from microarray analysis of gene expression data, real-time PCR was used to measure gene expression level changes with the same RNA samples used in the microarray experiment. We studied ten genes in the dilated cardiomyopathy (DCM) pathway that were identified as differentially expressed genes (p < 0.05, showing 20% up- or down-regulation), as well as two other genes in the DCM pathway with “borderline” difference (Adrb1, p < 0.06, 0.78-fold change; Prkx, p < 0.03, 0.94-fold change) because of their key positions in the DCM pathway; in addition, Jup was tested, as an internal control, because it was among those identified by microarray analysis, but was not situated in the β1AR-DHPR-Ca^2+^ branch of the DCM pathway. Seven candidate genes among the thirteen tested were validated by real-time PCR as differentially expressed genes, giving a 53.8% positive confirmation rate. Table 1 lists the seven genes that showed significantly differentiated expression between the chronic alcohol samples and the water control samples: Adrb1, Gnas, Adcy1, Cacna1d, and Itga4 with down-regulation, while Des and Igf1 with up-regulation. Interestingly, the four genes with the strongest statistical difference (Adrb1, Gnas, Adcy1, and Cacna1d) all showed down-regulation, and they all code for proteins which initiate the signaling steps in the β1AR-DHPR-Ca^2+^ branch of the DCM pathway (see diagram in Figure 5), suggesting that this branch may be impacted by chronic alcohol consumption during adolescence.

**Table 1:** RT-PCR confirmation of differential gene expression in hypothalamus between alcohol and water groups

**Gene symbol**	**Gene name**	**Microarray p-value**	**Microarray fold change**	**RT-PCR p-value**	**RT-PCR fold change**
Cacna1d	Calcium channel, voltage-dependent, L type, alpha 1D subunit	0.005*	0.748	0.0003*	0.609
Gnas	GNAS (guanine nucleotide binding protein, alpha stimulating) complex locus	0.006*	0.586	0.001*	0.604
Adrb1	Adrenergic receptor, beta 1	0.062	0.781	0.007*	0.815
Adcy1	Adenylate cyclase 1	0.030*	0.824	0.016*	0.517
Itga4	Integrin alpha 4	0.011*	0.634	0.021*	0.734
Adcy3	Adenylate cyclase 3	0.024*	1.103	0.342	0.896
Prkx	Protein kinase, X-linked	0.033*	0.940	0.477	0.812
Cacnb4	Calcium channel, voltage-dependent, beta 4 subunit	0.017*	1.085	0.377	0.911
Igf1	Insulin-like growth factor	0.012*	1.214	0.036*	1.284
Des	Desmin	0.004*	1.488	0.044*	1.367
Sgcd	Sarcoglycan, delta (dystrophin-associated glycoprotein)	0.013*	1.568	0.084	1.251
Tnni3	Troponin I, cardiac 3	0.041*	1.401	0.232	1.272

*p < 0.05.

## Discussion

In this study, we used mice to study chronic alcohol consumption during adolescence and its effect on gene expression and behavior in adulthood. We selected ICR mice for this study, instead of the more commonly used C57BL6 mice, for two reasons: (1) ICR mouse is an outbred mouse strain with inter-individual variability — similar to the outbred nature of humans. Using an outbred mouse strain such as ICR to study alcohol effect would more closely model the effect of alcohol in humans. (2) As an inbred strain, C57BL6 mice are known to drink considerably more alcohol than other mouse strains [46]. Thus, C57BL6 mice may represent only a special case regarding alcohol drinking behavior. Outbred mice such as ICR have been shown to consume less alcohol and exhibit lower preference for alcohol than C57BL6 mice [47]; in a preference test with 3% – 30% alcohol, the mean maximally preferred concentrations of alcohol were approximately 18% for C57BL6 mice vs. 7% for ICR mice [48]. We chose a two-bottle free-choice paradigm to measure alcohol drinking, as animals have unrestricted access to both water and alcohol for their own choice, thus better modeling the free-choice nature of human access to alcohol; such an approach has been used to identify the genetic basis for alcohol preference and dependence [49-56].

In our study, mice started chronic alcohol drinking from the age of about 4 weeks old and lasted seven weeks, covering the entire period of adolescence-to-adulthood development in mice. During this time, a trend of increased alcohol consumption was observed (Figure 1C), with an overall average alcohol consumption of 2.8 g/kg/day. This represents a moderate level of alcohol consumption, as it is comparable to the lower range of alcohol intake in a cross-mouse strain study of alcohol intake during the 4-hour daily drinking session using the drinking-in-the-dark model [46]. Using FDA’s guidance for conversion of animal doses to human equivalent doses (HED) based on body surface area [57], this level of alcohol consumption in mouse is roughly equivalent to about 1–2 glasses of wine in a 60 kg adult human. Interestingly, such a relatively moderate level of alcohol drinking during adolescent development still resulted in a pattern of gene expression change in hypothalamus; in particular, for certain genes that have been shown to be involved in the dilated cardiomyopathy (Figure 5).

### Adolescent chronic alcohol intake in mice results in a pattern of gene regulation in the hypothalamus

Our results showed that chronic alcohol intake influences hypothalamic gene expression. From our microarray analysis of hypothalamic gene expression, 1,819 genes showed significant changes, of which 751 displaying gene expression change of 1.2 fold or higher. PCA (Figure 3) and clustering analysis (Figure 4) showed that, compared with water controls, alcohol intake in mice displayed a pattern of hypothalamic gene expression changes. Using pathway analysis of the differentially expressed genes, we found that dilated cardiomyopathy (DCM) pathway is one of the major pathways that was affected by chronic alcohol intake. Using RT-PCR, five genes in the DCM pathway (Adrb1, Gnas, Adcy1, Cacna1d, and Itga4) are down-regulated in chronic alcohol mice relative to water controls, and two genes were up-regulated (Des and Igf1) (Table 1). Of the five down-regulated genes, four of them (Adrb1: β-1 adrenergic receptor, β1AR; Gnas: guanine nucleotide binding protein, alpha stimulating, Gsα; Adcy1: adenylyl cyclase 1, AC1; and Cacna1d: calcium channel, voltage-dependent, L type, alpha 1D subunit, D-LTCCs) belong to the β1AR-DHPR-Ca^2+^ branch of the DCM pathway (Figure 5). The functions of these gene products are closely related: β-adrenergic receptors are coupled to G proteins including Gnas, which stimulates adenylyl cyclase, and results in increased cAMP levels. The primary target for cAMP is protein kinase A, which phosphorylates several proteins including Cacna1d, which regulates Ca^2+^ influx into the cytosol from intracellular Ca^2+^ stores endoplasmic reticulum in neurons and sarcoplasmic reticulum in cardiac myocytes. What these genes share in common is that they all code for proteins which initiate the signaling steps of this pathway. Thus, we term these gene products “signaling pathway initiating factors” (SPIFs).

Our results corroborate with evidence from human and rodent studies where chronic alcohol treatment was used. In human, chronic alcoholism was shown to be related to the reduced Gsα levels in the temporal cortices of postmortem brains [58]. Also, postmortem brains of alcoholics showed a significant reduction of adenylyl cyclase 1 in both frontal and temporal cortices [59]. In addition, adenylyl cyclase mRNA levels in the blood were significantly lower in alcoholics than in controls [59]. Cyclic AMP levels were also decreased in lymphocytes of alcoholics [60]. Some of these molecular effects appear long-lasting. For example, in platelet membranes, adenylyl cyclase activity was lower in alcoholics who had abstained from alcohol for one to four years [61].

In rodents treated with chronic alcohol, it has been shown that in myocardial membranes, β-adrenergic receptor and cAMP levels were decreased [62], similar to our observations at the mRNA levels of these proteins (Table 1). In addition, β-adrenergic receptor density in the heart was decreased by alcohol exposure [63]. There are also ample evidence of chronic alcohol-related reduction in rodents for adenylyl cyclase and G proteins in the CNS, including cerebral cortex [64,65], pituitary [66], and cerebellum and pons [67]. These reports demonstrate that chronic alcohol treatment in vivo show a consistent pattern of reduction in β-1 adrenergic receptor, G protein, and adenylyl cyclase, consistent with our observations for the SPIFs, and suggesting that alcohol-induced impairment of these SPIFs may represent a rather common pathophysiological outcome from chronic alcohol exposure.

Of the down-regulated SPIF genes in the DCM pathway, the activity and function of Cacna1d in the hypothalamus was not well-understood. Cacna1d encodes the voltage-gated L-type Ca^2+^ channel formed by a1D subunits. Ca^2+^ channel involvement in cardiovascular function is known. For example, mice deficient of the alpha-1D subunit of the Ca^2+^ channel showed bradycardia and arrhythmia as a result of the sinoatrial node dysfunction [68-70]. D-LTCC channels are present in the atrial tissues of the heart where it contributes to pace-making [68]. D-LTCC channel opening results in Ca^2+^ influx, which activities ryanodine receptor, thus mediating Ca^2+^ release from intracellular Ca^2+^ stores such as sarcoplasmic reticulum [71], leading to cardiac muscle contraction. In the brain, D-LTCC channels are important in regulating neuronal activity [72,73]. In hippocampus, for example, these channels are involved in both long-term potentiation and long-term depression by coupling with ryanodine receptors [74]. These Ca^2+^ channel functions in the hypothalamus are little understood, however.

### Potential relationship between the hypothalamic gene regulation pattern and alcohol-induced cardiovascular disease

Regarding hypothalamic gene expression influenced by alcohol, there lacks reported studies about the four SPIF genes we studied. Based on the above-cited evidence of hypothalamus involvement in cardiovascular function, and of the hypothesized involvement of some of these genes in cardiovascular diseases, it is possible that the alcohol-mediated gene down-regulation in the hypothalamus that we observed is related to alcohol-induced cardiovascular disease. Our hypothesis is based on the following considerations:

1. Hypothalamus is a key CNS site for cardiovascular regulation. Hypothalamus is involved in many functions of the sympathetic nervous system, which tightly regulates cardiovascular activity [75-78]. Therefore, changes in hypothalamic gene expression are likely to influence the cardiovascular system, including cardiac muscles. Our study documented hypothalamic gene expression changes in the DCM pathway brought on by chronic alcohol consumption. Given the key role that hypothalamus plays in sympathetic regulation of cardiovascular function, the concerted gene down regulation pattern that we observed in hypothalamus is likely to be involved in regulating cardiovascular function.

2. Chronic alcohol intake can lead to cardiac muscle damage in human and animals, resulting in alcohol-induced cardiac muscle diseases, even heart failure. In dilated cardiomyopathy and heart failure patients, sympathetic system activation and cardiac β-1 adrenergic receptor down regulation are important characteristics of end-stage dilated cardiomyopathy and heart failure [79-81]. Alcohol can activate the sympathetic nervous system, leading to down regulation of cardiac βAR, Gs, and AC in human alcoholics and rats consuming chronic alcohol [60-63]. This is consistent with our observation that in the hypothalamus of chronic alcohol-drinking mice, the SPIFs of the DCM pathway showed concerted down regulation. Thus, chronic alcoholic intake leads to the DCM pathway SPIF down regulation in both the hypothalamus and the cardiovascular system. These results suggest that the SPIFs of the DCM pathway may play an important role in the development and progression of alcohol-mediated cardiovascular system injury and diseases (such as alcohol induced cardiac disease and heart failure).

3. Cacna1d codes for the dihydropyridine receptor (DHPR), which functions as a Ca2+ channel to affect Ca2+ influx across the cell membrane. In Cacna1d partial knockout mice, heart dysfunction was reported [68-70]. In neurons, Ca2+ channels mediate depolarization-induced Ca2+ influx across the plasma membrane of excitable cells, thus regulating the key physiological processes [68,82]. There is no prior report of alcohol-induced Cacna1d down regulation in the hypothalamus. In our study, the concerted down regulation of the SPIF genes in the hypothalamus includes Cacna1d. It is possible that decreased synaptic transmission and neuropeptide secretion in the hypothalamus may influence cardiovascular functions.

4. Epidemiological studies indicate that alcoholics who consume over 90 g of alcohol a day for over five years are at high risk for developing asymptomatic DCM; those who continue to drink alcohol for over 15 years may experience symptomatic DCM, leading to potential heart failure [83,84]. It is observed that with chronic alcohol consumption, metabolic changes occur within a few weeks [85,86], but signs of depressed contractile function are detectable only after several months of alcohol consumption [87,88].

In our study, the amount of alcohol consumed by mice is in the range of moderate levels, lasting about seven weeks. DCM pathway gene expression is already influenced in the hypothalamus under such conditions. This indicates two issues: (a) the drinking activities spanned the adolescence development, highlighting the danger of adolescent alcohol consumption even at moderate levels, and the potential harmful effect later in adulthood for cardiovascular function; and (b) gene activity changes in the hypothalamus may precede noticeable changes in body’s physiology and function, suggesting that these changes in DCM pathway genes may serve as early warning signs for alcohol’s harmful effect.

It should be pointed out that hypothalamus is a neuroanatomically heterogeneous brain structure, with various nuclei subserving diverse functions. Samples in our study encompassed much of the hypothalamus, therefore potentially diluting the extent of local gene expression changes. Future studies with more localized brain tissue collection will be helpful to delineate the roles of sub-hypothalamic regions in alcohol consumption and gene modulation. Another point to note is that, although the observed differences in alcohol consumption were not statistically significant between the 5% and 10% groups of mice, there still might be subtle differences in related gene expression changes, but our study design did not allow for distinguishing expression changes that are related to the 5% vs. 10% alcohol groups. Further studies with larger group sizes would offer improved opportunities to distinguish gene expression differences between different alcohol concentration groups. Additional limitations of this study relate to the fact that only hypothalamic tissues of the brain were studied for gene expression changes; it would be beneficial in future studies to examine cardiac tissues for alcohol-affected genes observed in this study, as a way to validate the findings of the present study. Another issue of note is that in our study, access to alcohol by mice was allowed to continue throughout the study, without any washout period. This study design was chosen to avoid any potential complication from alcohol withdrawal effect, both behaviorally and at the gene expression level, that would likely to occur if a no-drinking washout period were to be instituted. Thus, because of the continued access to alcohol, acute alcohol actions may contribute to the results observed in this study.

## Conclusions

This study showed that chronic alcohol consumption during adolescence, even at moderate levels, may trigger gene expression changes in the CNS that parallel those found in dilated cardiomyopathy (DCM). If such effects also take place in humans, our findings would serve as a warning against alcohol intake in youth, such as by teens and/or college students.

## Methods

### Animals

Male ICR (Institute for Cancer Research) outbred mice were obtained from Shanghai Laboratory Animal Center, Chinese Academy of Sciences, Shanghai, China. ICR outbred mice were also known as CD-1 (Caesarean Derived-1), and it is a commonly used outbred mouse strain. Animals were singly-housed in temperature controlled animal facilities on a 12 hr:12 hr light–dark cycle with food and water available ad libitum. Recording of water/alcohol consumption was conducted during the light cycle, about 4 hours after the light cycle began. Also, behavioral tests were conducted during the light cycle, beginning 3 hours after the light cycle began, and concluding no less than 2 hours before the end of the light cycle.

Principles of laboratory animal care were followed in accordance with the Guide for the Care and Use of Laboratory Animals (Institute of Laboratory Animal Resources, 1996), the PRC National Standards for Laboratory Animal Quality, and the Guidelines for the Use of Experimental Animals.

### Chronic alcohol self-administration

For the chronic alcohol drinking experiment, the paradigm of two-bottle choice was used [42]. Each cage was equipped with two drinking tubes. The drinking tubes were made from 10 ml plastic serological pipettes (Coster Stripette), with 0.1 ml graduation. The narrow tip of the pipette was cut off and a stainless steel sipper tube was connected through a silicone tubing. Animal’s body weight and the food consumption were measured on weekdays.

Before chronic alcohol drinking began, mice (3 weeks old) were allowed to adapt to the drinking tubes with both tubes containing water from experimental day 1–5. After this adaptation period, mice were randomly assigned to 5% alcohol group, 10% alcohol group, or water-only control group (n = 16–18). For the water-only control group, two drinking tubes in each cage contained deionized water throughout the duration of the experiment. For the alcohol groups, one drinking tube contained 5% or 10% alcohol solution while the other contained water. The amount of the water or alcohol consumption was recorded daily. The solution content (water or alcohol) was refreshed every day, and the positions of the two tubes in the cage were switched daily to avoid any side bias.

### Motor activities measurement

Behavior tests began on day 50 of the experiment. For motor activity measurement, an automated beam break detection system (San Diego Instruments) was used as previously described [89]. Animals were placed individually in plastic chambers (48 × 24 × 20 cm, LxWxH) with bedding material, and the chamber was covered with a piece of glass on the top. Each chamber was placed inside a wooden enclosure (60 × 40 × 44 cm, LxWxH) that was sound- and light-proof, illuminated from inside with a fluorescence light (5 W). Motor activity was measured via automated detection of infrared beam breaks. Rearing behavior was detected by a second set of infrared photodetectors mounted 7.7 cm above the ground.

### RNA extraction and DNA microarray

Chronic alcohol drinking lasted to day 57 of the experiment without any change and interruption. Then each mouse is sacrificed by decapitation, and the hypothalamus was dissected for rapid freeze and storage at -80°C. Hypothalamus tissue samples were used for total RNA extraction.

Total RNA was isolated using the TRIzol reagent (Invitrogen, Carlsbad, CA) and purified with RNeasy column (Qiagen, Valencia, CA). RNA concentration and purity were analyzed with Nanodrop spectrophotometer (Nanodrop Technologies, Wilmington, DE), with the spectral absorption at 260 and 280 nm. RNA integrity was assessed with Bioanalyzer 2100 (Agilent Technologies, Palo Alto, CA). All RNA samples had RNA Integrity Numbers (RIN) of 8.4 or higher and 28S/18S ratio of 1.5 or higher.

RNA samples were pooled for microarray. For water-only group, nine mice were used to make three pools, with three equal amounts of RNA samples per pool. For alcohol group, nineteen mice were used to make nine pools, with one to three equal RNA samples per pool, where samples A1-A3 had mice with relatively less alcohol consumed and samples A4-A9 had mice with relatively more alcohol consumed (Additional file 4: Table S3).

The microarray study was carried out as mono-color experiment. Total RNA was Cy3 labeled according to Agilent’s Low RNA Input Fluorescent Linear Amplification Kit and hybridized onto Agilent Whole Mouse Genome 4 × 44 K G4122F microarrays containing 43,604 probes as described in the manufacturer's protocol. Slides were scanned (Agilent G2505B) at 5 μm resolution using an extended dynamic range protocol, and images were processed with Agilent Feature Extraction software 9.5. Within-array normalization was performed using the “Background detrending” software (Agilent). The non-uniform outlier features (spots) were removed and the intensity values were transformed to a log base 2 scale. The microarray data have been deposited in Gene Expression Omnibus with the accession code GSE42770.

### Microarray data analysis

The spots with low signal noise ratio (<2) were automatically eliminated, and only “perfect value” of those genes present in > 50% samples in each group were applied in further analysis. Principal component analysis (PCA) was used to summarize gene expression profiles between groups. Differentially expressed genes (DEGs) were determined by comparing with the control group, using a corresponding *t*-test with the significance level set at *p*-value < 0.05 and *q*-value (false discover rate) at < 0.05 [90]. Hierarchical clustering and visualization were performed using Cluster 3.0 and TreeView software (M. B. Eisen Laboratory, Stanford University, Stanford, CA). Average linkage with the uncentered correlation similarity metric was used for the clustering of samples. Pathway analysis was carried out using DAVID Functional Classification tool [91], the open source pathway resources of Kyoto Encyclopedia of Genes and Genomes (KEGG) (http://www.genome.jp/kegg/) [92], and the Web-based Gene Set Analysis Toolkit (WebGestalt) [93].

### Real-time PCR

Based on our analysis of significant biological pathways that are associated with the identified differentially expressed genes, real-time PCR was used to verify the status of differential expression and to exclude false positives. For internal control, we initially evaluated two genes (β-actin and glyceraldehyde-3-phosphate dehydrogenase, or Gapdh). Both β-actin and Gapdh showed stable expression in each sample in the microarray, therefore they both qualified as internal control at this point. From there, we used qRT-PCR to measure both genes’ expression levels in our samples (C1-C3, A1-A9), utilizing the NormFinder program [94]. The NormFinder software adopts a model-based variance estimation approach to identify genes suited for normalization, and provides a rank order according to gene’s expression stability. According to this program, β-actin was identified as the more stably expressed gene, and was therefore selected as reference gene for gene expression normalization in the qRT-PCR experiment. The sequences of the primers for the target genes and the internal control gene (β-actin) are listed in Additional file 5: Table S2.

For cDNA synthesis, oligo(dT) primers, 2 μg of each total RNA sample used in microarray experiment, and the Superscript II reverse transcriptase (Invitrogen) were used, following the guidelines of the manufacturer. cDNA samples were placed on ice and stored at -20°C until further use. Prior to the analysis, 20 μl of each cDNA sample was diluted with 360 μl of MilliQ water. qPCR reactions were performed with the Prism 7900 Sequence Detection System (Applied Biosystems, Foster City, CA). For each reaction, 1 μl of each diluted cDNA sample was added to a mixture containing 10 μl of 2 × SYBR green II qRT-PCR kit (Toyobo, Osaka, Japan), 1 μl of each primer (5 μM), and 8 μl of MilliQ water. Cycling conditions were 10 min 95°C, followed by 40 cycles of 15 s at 95°C and 1 min at 60°C. After cycling, a melting protocol was performed with 15 s at 95°C, 1 min at 60°C, and 15 s at 95°C, to control for product specificity.

The fold change (FC) in target gene cDNA relative to selected endogenous control gene was determined as follows: FC = 2^-∆∆Ct^, where ∆∆Ct = (Ct_Target_ - Ct_Control_)test - (Ct_Target_ - Ct_Control_)control. Ct values were defined as the number of the PCR cycles at which the fluorescence signals were detected. In qRT-PCR analysis, data are presented as mean ± SEM and analyzed with *t*-test by SPSS 13.0 (SPSS Inc, Chicago, IL).

### Statistics

Repeated measures two-way ANOVA with Bonferroni post tests was used to compare the two chronic alcohol groups (5% and 10%) in daily alcohol consumption. Repeated measures ANOVA with post test of Dunnett's multiple comparison test was used to compare daily alcohol consumption between the first time point and other time points. Unpaired t-test was used to compare the chronic alcohol and water-only control mice in locomotion and rearing behavior.

## Competing interests

The authors declare that they have no competing interests.

## Authors’ contributions

HZ, study design, literature search, chronic alcohol experiment, behavior experiment, behavior data analysis, statistical analysis, writing of the manuscript. KW, microarray gene expression experiment and data analysis, RT-PCR, statistical analysis, writing of the manuscript. YG and HS, microarray gene expression experiment and data analysis, RT-PCR. QX, chronic alcohol experiment, behavior experiment. MJ and GZ, study design, editing of the manuscript. HX, study design, microarray gene expression experiment and data analysis, editing of the manuscript. LY, study design, data analysis, writing manuscript, supervision of the study. All authors contributed to and have approved the final manuscript.

## Supplementary Material

Additional file 1: Table S1 List of differentially expressed genes identified in microarray analysis between chronic alcohol consumption and water-only control. Click here for file

Additional file 2: Figure S1 Heat map of genes differentially expressed between chronic alcohol vs. control mouse hypothalamus samples. Gene expression values are color-coded according to the scale on the right. Displayed on top is the clustering display of differentially expressed genes using the unsupervised hierarchical clustering method. C1-C3: water-only control samples; A1-A9: chronic alcohol samples. Click here for file

Additional file 3: Table S4 Top 5 pathways from DAVID Functional Classification Analysis and Web-based Gene Set Analysis. Click here for file

Additional file 4: Table S3 Pooling of RNA samples from alcohol mice. Click here for file

Additional file 5: Table S2 Sequences of primers for qRT-PCR. Click here for file
