# Supplementary material for: Chronic alcohol consumption from adolescence-to-adulthood in mice - hypothalamic gene expression changes in the dilated cardiomyopathy signaling pathway
Source: BMC Neurosci. 2014 May 9;15:61. doi: 10.1186/1471-2202-15-61 (PMC4027996; doi:10.1186/1471-2202-15-61)
Supplement: Additional file 1: Table S1 — List of differentially expressed genes identified in microarray analysis between chronic alcohol consumption and water-only control. [file 1471-2202-15-61-S1.doc]

Supplemental Table S1. List of differentially expressed genes identified in microarray analysis between chronic alcohol consumption and water-only control, ordered by fold change values (largest to smallest)

| Probe Name | Gene Symbol | p-values | Fold Change |
| --- | --- | --- | --- |
| A_52_P137500 | Fam199x | 0.009647 | 3.437374 |
| A_52_P420479 | Hand2 | 0.000476 | 2.58634 |
| A_52_P129238 | Adck1 | 0.006588 | 2.467718 |
| A_52_P499158 | Tex101 | 0.026648 | 2.432183 |
| A_51_P194503 | Olfr433 | 0.001845 | 2.394766 |
| A_51_P477604 | 4933412E24Rik | 0.024153 | 2.33567 |
| A_52_P235880 | Gm5134 | 0.001866 | 2.285832 |
| A_51_P148602 | Cxcr2 | 0.037239 | 2.267469 |
| A_51_P176086 | Ffar2 | 0.002581 | 2.195798 |
| A_51_P230298 | Hdgfl1 | 0.02317 | 2.193048 |
| A_52_P351859 | Hoxc10 | 0.03151 | 2.182479 |
| A_52_P84347 | 4931419H13Rik | 0.025122 | 2.178896 |
| A_52_P244005 | Cd80 | 0.037267 | 2.170398 |
| A_51_P305138 | A_51_P305138 | 0.014045 | 2.142748 |
| A_51_P319386 | Myo15 | 0.034908 | 2.11301 |
| A_51_P438752 | Gm4221 | 0.017936 | 2.112928 |
| A_52_P402104 | NAP063697-1 | 0.003631 | 2.110713 |
| A_51_P121607 | ENSMUST00000025428 | 0.003844 | 2.081125 |
| A_51_P187898 | H60a | 0.018505 | 2.076254 |
| A_51_P312615 | Hist1h1t | 0.008196 | 2.064466 |
| A_51_P311958 | Orm3 | 0.021355 | 2.044113 |
| A_51_P330353 | Pabpc6 | 0.001991 | 2.028968 |
| A_51_P206235 | Slc39a5 | 0.041585 | 2.028306 |
| A_51_P309589 | 2700099C18Rik | 0.004605 | 2.01521 |
| A_52_P511177 | TC1648263 | 0.009299 | 2.003253 |
| A_51_P507242 | Fosl2 | 0.016088 | 2.002214 |
| A_52_P476902 | Chd6 | 0.001747 | 1.990998 |
| A_52_P343569 | ENSMUST00000033963 | 0.002689 | 1.963256 |
| A_52_P13326 | Barhl2 | 0.006939 | 1.960216 |
| A_51_P218924 | ENSMUST00000060246 | 0.038801 | 1.956668 |
| A_52_P360241 | Prickle2 | 0.022468 | 1.924762 |
| A_52_P670612 | Meis1 | 0.010136 | 1.886717 |
| A_52_P17466 | TC1633545 | 0.000724 | 1.880044 |
| A_51_P235002 | Fabp9 | 0.006294 | 1.879341 |
| A_51_P130727 | Fkbp11 | 0.016814 | 1.863051 |
| A_51_P501873 | Krt26 | 0.002362 | 1.855484 |
| A_51_P352027 | TC1686515 | 0.007554 | 1.854663 |
| A_51_P328239 | Polr3b | 0.003662 | 1.849872 |
| A_51_P267836 | Plg | 0.011869 | 1.849838 |
| A_52_P579517 | Cox10 | 0.019341 | 1.84426 |
| A_51_P512306 | Kirrel2 | 0.032731 | 1.844085 |
| A_52_P663096 | Dgke | 0.011015 | 1.842669 |
| A_51_P303316 | Hoxa13 | 0.004352 | 1.830427 |
| A_52_P103683 | Fam178b | 0.009771 | 1.822117 |
| A_52_P61880 | Cmya5 | 0.047755 | 1.820713 |
| A_51_P191262 | Jsrp1 | 0.001604 | 1.814655 |
| A_52_P171212 | Irs1 | 0.006428 | 1.811436 |
| A_52_P571175 | NAP071276-1 | 0.004981 | 1.810223 |
| A_51_P216679 | Anxa10 | 0.039703 | 1.801879 |
| A_51_P499623 | Mlana | 0.034039 | 1.801207 |
| A_51_P392654 | Tulp1 | 0.01035 | 1.798901 |
| A_51_P449638 | Pldi | 0.010779 | 1.798386 |
| A_51_P242460 | Fgf14 | 0.002165 | 1.795466 |
| A_51_P389885 | Spic | 0.003884 | 1.788685 |
| A_51_P358256 | Nr1i3 | 0.005609 | 1.78537 |
| A_52_P646066 | Zfp609 | 0.002907 | 1.785334 |
| A_52_P356419 | Tmem95 | 0.03272 | 1.784712 |
| A_51_P487690 | Ifi44 | 0.04705 | 1.779722 |
| A_52_P519191 | Dio3os | 0.007122 | 1.774115 |
| A_51_P399845 | Fgf2 | 0.002036 | 1.771594 |
| A_51_P271832 | 2210009G21Rik | 0.037583 | 1.767853 |
| A_52_P499027 | Eif4e2 | 0.015381 | 1.75582 |
| A_51_P480153 | Pdcl | 0.034196 | 1.754594 |
| A_52_P450214 | Aipl1 | 0.046745 | 1.74847 |
| A_52_P546363 | Gata3 | 0.006662 | 1.747191 |
| A_51_P487950 | Myo1a | 0.006889 | 1.739002 |
| A_52_P411205 | ENSMUST00000076182 | 0.025495 | 1.728306 |
| A_52_P564444 | A530064D06Rik | 0.011841 | 1.715464 |
| A_52_P572659 | NAP030565-1 | 0.013054 | 1.708733 |
| A_51_P201035 | Cenpi | 0.00431 | 1.706907 |
| A_51_P380078 | Fcgbp | 0.045329 | 1.703972 |
| A_52_P127842 | TC1619269 | 0.013008 | 1.690588 |
| A_51_P164064 | Disc1 | 0.006076 | 1.688317 |
| A_52_P374676 | NAP123498-1 | 0.009772 | 1.682371 |
| A_52_P646366 | Zc3h3 | 0.002819 | 1.673529 |
| A_51_P408227 | 9130017N09Rik | 0.007046 | 1.664305 |
| A_51_P412926 | Krt27 | 0.048359 | 1.662768 |
| A_52_P988778 | A_52_P988778 | 0.042553 | 1.641682 |
| A_51_P400217 | Vpreb1 | 0.048627 | 1.639242 |
| A_51_P439426 | Acaca | 0.015146 | 1.631924 |
| A_51_P104478 | Smpx | 0.027268 | 1.624501 |
| A_51_P350395 | AK078791 | 0.018431 | 1.621854 |
| A_52_P548202 | Gm10030 | 0.011196 | 1.615792 |
| A_51_P333460 | 5430427O19Rik | 0.034109 | 1.613794 |
| A_51_P354018 | Kcnk7 | 0.044947 | 1.612436 |
| A_51_P106900 | Rax | 0.006206 | 1.608236 |
| A_51_P342276 | Palm2 | 0.02109 | 1.606134 |
| A_52_P755756 | AK087246 | 0.017941 | 1.604284 |
| A_51_P393563 | A_51_P393563 | 0.014055 | 1.603793 |
| A_51_P305896 | Cftr | 0.004352 | 1.60176 |
| A_52_P346244 | Efcab1 | 0.014002 | 1.599458 |
| A_52_P678117 | Xpr1 | 0.009877 | 1.596383 |
| A_52_P77155 | Syt10 | 0.014653 | 1.59632 |
| A_52_P276869 | C130026L21Rik | 0.040402 | 1.590353 |
| A_52_P18887 | Sprr2a1 | 0.020027 | 1.58913 |
| A_52_P658241 | Ogt | 0.001851 | 1.585834 |
| A_52_P291562 | AK015959 | 0.018959 | 1.585781 |
| A_51_P110168 | Ing5 | 0.00757 | 1.58452 |
| A_52_P558536 | Zfp30 | 0.00044 | 1.583574 |
| A_52_P485427 | Mll2 | 0.011547 | 1.582755 |
| A_52_P149766 | Myo1e | 0.020078 | 1.579751 |
| A_52_P321140 | Defb1 | 0.044822 | 1.579464 |
| A_51_P343517 | Ly6d | 0.042648 | 1.572657 |
| A_51_P389004 | Sgcd | 0.013061 | 1.568325 |
| A_52_P662622 | A_52_P662622 | 0.036657 | 1.566184 |
| A_51_P346445 | Hspb7 | 0.008111 | 1.564319 |
| A_51_P195129 | Shroom3 | 0.027936 | 1.563309 |
| A_51_P430540 | AK081855 | 0.005922 | 1.562495 |
| A_51_P502872 | 2200002D01Rik | 0.000603 | 1.562009 |
| A_52_P189984 | Lrrc1 | 0.000985 | 1.561901 |
| A_52_P62484 | D630042P16Rik | 0.015491 | 1.549647 |
| A_51_P115656 | Iqcd | 0.00713 | 1.549529 |
| A_52_P818682 | 9530006C21Rik | 0.003228 | 1.549014 |
| A_51_P417643 | Foxa1 | 0.005254 | 1.548321 |
| A_51_P358714 | AK031433 | 0.040487 | 1.545222 |
| A_51_P476518 | Fam83g | 0.030892 | 1.535902 |
| A_51_P132551 | Trip10 | 0.027124 | 1.531815 |
| A_52_P222775 | Map3k6 | 0.005743 | 1.529921 |
| A_51_P273489 | Acap1 | 0.00476 | 1.527807 |
| A_52_P493502 | Gm9806 | 0.0229 | 1.525955 |
| A_52_P270429 | 2200001I15Rik | 0.031168 | 1.523116 |
| A_52_P9489 | NAP046089-1 | 0.029501 | 1.517742 |
| A_52_P675052 | Golgb1 | 0.044657 | 1.516306 |
| A_51_P320291 | Kcnab1 | 0.004482 | 1.515505 |
| A_52_P197179 | TC1665684 | 0.020351 | 1.515367 |
| A_52_P158029 | NAP061005-1 | 0.013796 | 1.514661 |
| A_52_P27878 | E130306D19Rik | 0.028093 | 1.514328 |
| A_52_P336977 | Nat15 | 0.007094 | 1.513027 |
| A_52_P306421 | Lnx2 | 0.037252 | 1.508341 |
| A_52_P418884 | Nedd9 | 0.026879 | 1.507911 |
| A_52_P18705 | Osbpl1a | 0.001143 | 1.505516 |
| A_52_P141384 | Cdcp1 | 0.018483 | 1.504136 |
| A_51_P204121 | Nudt6 | 0.01895 | 1.503866 |
| A_51_P295511 | Aanat | 0.045497 | 1.499732 |
| A_51_P217498 | Slc2a4 | 7.76E-05 | 1.499484 |
| A_51_P464600 | Emilin3 | 0.040224 | 1.4988 |
| A_52_P275080 | CD555094 | 0.028752 | 1.498178 |
| A_52_P396401 | Rph3al | 0.033234 | 1.496863 |
| A_52_P629907 | ENSMUST00000055369 | 0.004165 | 1.496568 |
| A_51_P153079 | Ttll3 | 0.014905 | 1.496177 |
| A_51_P282940 | AU021034 | 0.029422 | 1.495961 |
| A_51_P294375 | 5730559C18Rik | 0.018836 | 1.494359 |
| A_51_P377154 | Cyp2a4 | 0.04648 | 1.492338 |
| A_52_P561272 | Ky | 0.04423 | 1.492304 |
| A_51_P117711 | Bat2l2 | 0.021224 | 1.492092 |
| A_51_P389785 | Sf3a2 | 0.020147 | 1.488163 |
| A_51_P335969 | Des | 0.003549 | 1.487852 |
| A_51_P299858 | 1700125H20Rik | 0.020982 | 1.48739 |
| A_52_P193424 | Alkbh8 | 0.010353 | 1.48595 |
| A_51_P153693 | Utp14b | 0.012136 | 1.482308 |
| A_51_P256533 | Nxf2 | 0.048986 | 1.480042 |
| A_51_P232617 | Spag17-ps | 0.003315 | 1.47833 |
| A_52_P657360 | Tnni1 | 0.042194 | 1.477139 |
| A_52_P512110 | Ccdc109a | 0.036785 | 1.476995 |
| A_52_P102630 | Rian | 0.035238 | 1.476886 |
| A_51_P291210 | Afap1 | 0.004319 | 1.476318 |
| A_52_P36261 | TC1645771 | 0.015986 | 1.463583 |
| A_52_P219314 | Vasp | 0.001623 | 1.461391 |
| A_52_P106513 | Brap | 0.01745 | 1.461191 |
| A_52_P40345 | Ppp1r14d | 0.03726 | 1.46118 |
| A_51_P251032 | AK089906 | 0.029049 | 1.460616 |
| A_51_P324524 | Calcoco2 | 0.03353 | 1.460118 |
| A_52_P385375 | Angel2 | 0.00705 | 1.454251 |
| A_51_P382331 | Gm16491 | 0.029095 | 1.45399 |
| A_52_P32121 | Ckap4 | 0.008555 | 1.453547 |
| A_52_P246255 | Cyp2a4 | 0.00782 | 1.451611 |
| A_52_P118624 | AK046951 | 0.004727 | 1.449669 |
| A_51_P261560 | 1700061J05Rik | 0.002942 | 1.449633 |
| A_52_P117408 | Tg | 0.030923 | 1.446511 |
| A_52_P218990 | H2afy2 | 0.003974 | 1.443325 |
| A_52_P597800 | Etv6 | 0.013734 | 1.442516 |
| A_52_P421399 | Cul5 | 0.006645 | 1.44128 |
| A_51_P416647 | Egfbp2 | 0.00178 | 1.43957 |
| A_51_P209736 | Atoh8 | 0.011663 | 1.439173 |
| A_51_P355258 | 4930534B04Rik | 0.001082 | 1.437251 |
| A_52_P641922 | Nhlrc3 | 0.047039 | 1.432592 |
| A_52_P663686 | I830012O16Rik | 0.018278 | 1.431617 |
| A_52_P163790 | Mageb16 | 0.009827 | 1.428378 |
| A_51_P247694 | Gpr97 | 0.029107 | 1.428118 |
| A_52_P602292 | Gm270 | 0.030532 | 1.425803 |
| A_52_P88296 | NAP057940-1 | 0.04443 | 1.42017 |
| A_51_P230142 | A930018P22Rik | 0.015567 | 1.419898 |
| A_51_P442023 | Kif17 | 0.000566 | 1.418464 |
| A_52_P174328 | Pde4dip | 0.046838 | 1.418173 |
| A_52_P280668 | Gm5797 | 0.035163 | 1.417981 |
| A_51_P486223 | Th | 0.039539 | 1.417018 |
| A_51_P371190 | Pttg1 | 0.020092 | 1.41621 |
| A_51_P356413 | Foxk1 | 0.026953 | 1.414131 |
| A_51_P312121 | Xdh | 0.004071 | 1.413743 |
| A_51_P230537 | Ccdc114 | 0.023887 | 1.410777 |
| A_51_P170807 | Map3k6 | 0.015951 | 1.406205 |
| A_51_P403578 | 1810059H22Rik | 0.042896 | 1.405918 |
| A_51_P474103 | 9430020K01Rik | 0.03655 | 1.405172 |
| A_51_P464539 | Phldb2 | 0.048911 | 1.403591 |
| A_51_P171531 | 2900006K08Rik | 0.021752 | 1.402414 |
| A_51_P386983 | Tnni3 | 0.041116 | 1.400991 |
| A_52_P246698 | Fam126a | 0.049637 | 1.400558 |
| A_51_P217697 | 1700019L03Rik | 0.032704 | 1.398809 |
| A_52_P599578 | Cald1 | 0.003713 | 1.397427 |
| A_51_P196087 | Nav1 | 0.043669 | 1.395006 |
| A_51_P219109 | Il12rb1 | 0.000112 | 1.393631 |
| A_52_P551544 | Grm7 | 0.010511 | 1.391299 |
| A_51_P486217 | Wnk4 | 0.010468 | 1.389939 |
| A_51_P468658 | Aldh1l2 | 0.032512 | 1.389408 |
| A_51_P331409 | Tacc1 | 0.031874 | 1.389186 |
| A_52_P381562 | AK035243 | 0.006556 | 1.38445 |
| A_52_P574527 | Zfp295 | 0.0047 | 1.3843 |
| A_52_P673421 | Reep3 | 0.001001 | 1.382838 |
| A_51_P269792 | Rad51l1 | 0.014762 | 1.382707 |
| A_52_P414282 | Cgn | 0.016042 | 1.382472 |
| A_52_P43266 | Abt1 | 0.027702 | 1.379595 |
| A_51_P207031 | Ncf1 | 0.015241 | 1.378746 |
| A_52_P255489 | Usp2 | 0.026344 | 1.377977 |
| A_51_P461444 | Dhfr | 0.016762 | 1.375551 |
| A_52_P403827 | St6gal2 | 0.048846 | 1.374167 |
| A_52_P325477 | Trim16 | 0.026294 | 1.372182 |
| A_52_P439874 | Arfgap3 | 0.022241 | 1.368338 |
| A_51_P517525 | Tmem169 | 0.035376 | 1.365535 |
| A_52_P342202 | Mier1 | 0.041663 | 1.364948 |
| A_52_P231762 | BQ563014 | 0.045705 | 1.364904 |
| A_52_P635312 | Irx2 | 0.024711 | 1.364661 |
| A_52_P443846 | Ppfibp1 | 0.01497 | 1.362663 |
| A_51_P305583 | Sp100 | 0.009923 | 1.362115 |
| A_51_P360122 | 2610028H24Rik | 0.045056 | 1.356739 |
| A_51_P515684 | 1700001L05Rik | 0.02286 | 1.35586 |
| A_52_P462837 | Emx2 | 0.028839 | 1.355405 |
| A_52_P233342 | Trim15 | 0.031979 | 1.354898 |
| A_51_P273157 | Pde6h | 0.014631 | 1.354366 |
| A_52_P315837 | Prx | 0.028988 | 1.353508 |
| A_51_P495242 | Lat | 0.016066 | 1.353368 |
| A_52_P493370 | Ankrd24 | 0.017238 | 1.351501 |
| A_52_P265979 | Arfrp1 | 0.016169 | 1.349315 |
| A_52_P84415 | L3mbtl2 | 0.026989 | 1.348238 |
| A_51_P396198 | C4bp-ps1 | 0.020841 | 1.347794 |
| A_51_P302398 | 1700028J19Rik | 0.037205 | 1.34738 |
| A_51_P517126 | Tceb3 | 0.023584 | 1.347032 |
| A_52_P282091 | Zfp386 | 0.027004 | 1.345985 |
| A_51_P151505 | Zfp318 | 0.023405 | 1.345859 |
| A_52_P401386 | Jup | 0.004256 | 1.344187 |
| A_52_P26265 | ENSMUST00000115107 | 0.016242 | 1.34394 |
| A_52_P509965 | Ager | 0.001432 | 1.343245 |
| A_51_P487010 | Pou3f2 | 0.005417 | 1.34201 |
| A_52_P258507 | XM_001477157 | 0.014635 | 1.340634 |
| A_51_P306710 | Cldn15 | 0.047573 | 1.340023 |
| A_52_P662796 | Dennd2d | 0.012923 | 1.339044 |
| A_51_P100856 | Fn1 | 0.018715 | 1.337918 |
| A_52_P219194 | Kcnq3 | 0.021334 | 1.337204 |
| A_52_P626069 | Chd9 | 0.039009 | 1.335746 |
| A_52_P301103 | Tob2 | 0.019567 | 1.33368 |
| A_52_P485417 | Hmgb2 | 0.0075 | 1.33296 |
| A_52_P1140283 | AK043080 | 0.029369 | 1.332866 |
| A_51_P260167 | Gpr27 | 0.048037 | 1.330163 |
| A_51_P157042 | Ctgf | 0.009997 | 1.330106 |
| A_51_P124388 | Slc45a3 | 0.049125 | 1.328663 |
| A_51_P417854 | Rab20 | 0.04309 | 1.328638 |
| A_52_P241725 | C330018D20Rik | 0.034588 | 1.328461 |
| A_51_P324535 | B4galt5 | 0.034974 | 1.327828 |
| A_52_P681727 | Eif2ak1 | 0.003675 | 1.326551 |
| A_52_P255841 | Dbr1 | 0.008258 | 1.326283 |
| A_51_P406835 | Mei1 | 0.017051 | 1.325984 |
| A_51_P260889 | Cd3g | 0.020899 | 1.325619 |
| A_52_P118516 | Heatr7a | 0.004086 | 1.325318 |
| A_52_P496133 | Spats1 | 0.044686 | 1.323652 |
| A_51_P234407 | Tjp3 | 0.013017 | 1.322839 |
| A_52_P572045 | Pfkp | 0.036875 | 1.320194 |
| A_52_P214976 | AK031369 | 0.017241 | 1.31912 |
| A_51_P300666 | Npb | 0.042664 | 1.315833 |
| A_51_P197252 | Zfp830 | 0.020626 | 1.315807 |
| A_51_P381618 | Pla1a | 0.015364 | 1.315041 |
| A_52_P450528 | ENSMUST00000068009 | 0.035085 | 1.314382 |
| A_51_P158216 | Fyco1 | 0.033214 | 1.311656 |
| A_51_P486497 | ENSMUST00000101077 | 0.024081 | 1.311088 |
| A_52_P123655 | ENSMUST00000059200 | 0.046687 | 1.310794 |
| A_52_P638337 | 5031410I06Rik | 0.008736 | 1.309611 |
| A_52_P339422 | NAP046281-1 | 0.016935 | 1.308916 |
| A_51_P507982 | Stk3 | 0.002374 | 1.308586 |
| A_52_P92213 | 2210404J11Rik | 0.046558 | 1.307535 |
| A_52_P541802 | Ifitm1 | 0.0305 | 1.306988 |
| A_52_P265578 | Vmo1 | 0.039398 | 1.306574 |
| A_52_P600474 | Kif17 | 0.024533 | 1.305958 |
| A_52_P1101113 | A_52_P1101113 | 0.004133 | 1.305546 |
| A_52_P18765 | Hsbp1l1 | 0.02769 | 1.304503 |
| A_52_P181468 | Setd8 | 0.009076 | 1.304052 |
| A_51_P221072 | C1qtnf7 | 0.013064 | 1.303403 |
| A_51_P184936 | Zbp1 | 0.049559 | 1.301709 |
| A_51_P285906 | LOC100049077 | 0.029429 | 1.30134 |
| A_51_P137808 | Zfp26 | 0.043821 | 1.300725 |
| A_51_P395014 | Ndufs3 | 0.042799 | 1.298715 |
| A_51_P317286 | ENSMUST00000100190 | 0.03509 | 1.297922 |
| A_51_P387868 | Tekt4 | 0.003341 | 1.297418 |
| A_52_P1011737 | AK039098 | 0.043884 | 1.293313 |
| A_51_P270949 | Hist1h1b | 0.038017 | 1.292147 |
| A_52_P321115 | Nufip1 | 0.025519 | 1.291593 |
| A_52_P518434 | Hmbox1 | 0.04458 | 1.290313 |
| A_51_P251465 | Exosc1 | 0.034401 | 1.289535 |
| A_52_P495372 | Peo1 | 0.035328 | 1.288614 |
| A_52_P346256 | Ptcd3 | 0.011366 | 1.288461 |
| A_52_P224844 | ENSMUST00000035327 | 0.007147 | 1.284795 |
| A_52_P525161 | Ntf3 | 0.007861 | 1.283874 |
| A_52_P100738 | TC1632787 | 0.034461 | 1.282415 |
| A_52_P257502 | Igfbp4 | 0.011476 | 1.28199 |
| A_51_P475858 | Pvrl2 | 0.022239 | 1.281379 |
| A_52_P219549 | Cdkl5 | 0.039699 | 1.28103 |
| A_52_P61735 | Flnc | 0.027638 | 1.279968 |
| A_51_P201390 | Tbc1d4 | 0.018971 | 1.279817 |
| A_52_P381430 | Tbc1d4 | 0.033589 | 1.279535 |
| A_52_P271244 | Pold3 | 0.012768 | 1.279447 |
| A_52_P298291 | AK040437 | 0.025905 | 1.277741 |
| A_51_P129480 | Ccl21a | 0.037471 | 1.275744 |
| A_51_P233226 | Atl3 | 0.035309 | 1.275705 |
| A_52_P87513 | NAP101973-1 | 0.007926 | 1.275399 |
| A_51_P503192 | Rbm15b | 0.001787 | 1.275319 |
| A_52_P85495 | BC001981 | 0.048271 | 1.274584 |
| A_51_P377094 | Col1a1 | 0.010999 | 1.273522 |
| A_52_P338651 | Pitpnm2 | 0.028493 | 1.272607 |
| A_52_P1084729 | F830010H11Rik | 0.027931 | 1.272245 |
| A_51_P399132 | A_51_P399132 | 0.04109 | 1.272012 |
| A_52_P173945 | BC048546 | 0.013957 | 1.27182 |
| A_51_P447835 | Pla2g7 | 0.048668 | 1.270605 |
| A_51_P424990 | Klhdc7a | 0.048392 | 1.270414 |
| A_52_P527834 | Grid1 | 0.006133 | 1.270116 |
| A_51_P520966 | Icosl | 0.018148 | 1.269474 |
| A_51_P425047 | Srpx2 | 0.019155 | 1.269189 |
| A_51_P235821 | Tsc22d3 | 0.018286 | 1.266784 |
| A_52_P368650 | Zswim4 | 0.001396 | 1.265682 |
| A_52_P85418 | Ankrd26 | 0.013341 | 1.265156 |
| A_51_P462428 | Galntl2 | 0.000823 | 1.263641 |
| A_52_P93467 | Sdc4 | 0.024974 | 1.262728 |
| A_51_P182796 | Tgm2 | 0.026734 | 1.260891 |
| A_52_P392456 | Rnd3 | 0.043537 | 1.260875 |
| A_52_P288997 | A630007B06Rik | 0.01752 | 1.260488 |
| A_51_P386270 | Cyp2d12 | 0.015059 | 1.260017 |
| A_52_P141404 | Rcor3 | 0.009579 | 1.259637 |
| A_52_P169181 | Auts2 | 0.004061 | 1.258669 |
| A_51_P464420 | 4921508M14Rik | 0.042177 | 1.25826 |
| A_52_P647055 | Zfp553 | 0.001082 | 1.256761 |
| A_52_P327923 | Meox1 | 0.033781 | 1.255868 |
| A_51_P235816 | Tsc22d3 | 0.027498 | 1.255514 |
| A_51_P159746 | Nkap | 0.000628 | 1.25485 |
| A_51_P262340 | Rbm3 | 0.015389 | 1.252667 |
| A_52_P480872 | Cerk | 0.027307 | 1.250532 |
| A_51_P378589 | Myof | 0.004222 | 1.250485 |
| A_52_P177988 | Fam179b | 0.009515 | 1.249858 |
| A_51_P241552 | Wdr37 | 0.021418 | 1.249452 |
| A_52_P380502 | Plec | 0.028438 | 1.248182 |
| A_51_P337101 | Rnf43 | 0.012436 | 1.246412 |
| A_51_P155482 | Pole | 0.030723 | 1.246129 |
| A_51_P451409 | Ttll13 | 0.009947 | 1.246125 |
| A_52_P73307 | Mtbp | 0.000448 | 1.243723 |
| A_52_P117029 | Safb2 | 0.00428 | 1.241882 |
| A_52_P571746 | Sh2d5 | 0.039976 | 1.24148 |
| A_52_P432852 | Ubtd2 | 0.014234 | 1.240948 |
| A_52_P388063 | Tlk1 | 0.038238 | 1.240943 |
| A_51_P139780 | Pglyrp1 | 0.004488 | 1.240657 |
| A_51_P165882 | Slc22a18 | 0.021173 | 1.239587 |
| A_52_P1197518 | TC1582889 | 0.019507 | 1.238991 |
| A_51_P367734 | Plcl1 | 0.004137 | 1.238604 |
| A_52_P407968 | ENSMUST00000023427 | 0.028837 | 1.238411 |
| A_52_P392229 | Rnf152 | 0.02911 | 1.23721 |
| A_51_P486289 | D5Ertd579e | 0.036192 | 1.234742 |
| A_52_P270384 | Sobp | 0.019086 | 1.233899 |
| A_52_P949005 | Gm6809 | 0.006641 | 1.233892 |
| A_52_P600822 | Prkcz | 0.010934 | 1.233644 |
| A_51_P282760 | Per2 | 0.010624 | 1.233106 |
| A_52_P321847 | Ube2j2 | 0.017772 | 1.232796 |
| A_52_P595389 | Unc80 | 0.013353 | 1.232361 |
| A_51_P352385 | Ism1 | 0.025463 | 1.231824 |
| A_52_P543040 | Utp14a | 0.041201 | 1.230454 |
| A_52_P161297 | Tcea3 | 0.033502 | 1.22991 |
| A_52_P581138 | Ctdspl2 | 0.031691 | 1.227796 |
| A_51_P258894 | Chst2 | 0.029321 | 1.226896 |
| A_52_P673519 | Nek1 | 0.042289 | 1.226439 |
| A_52_P320170 | Kdm4c | 0.035421 | 1.226219 |
| A_51_P478353 | Myof | 0.031542 | 1.225901 |
| A_51_P407311 | Aldh16a1 | 0.006555 | 1.223613 |
| A_52_P201494 | ENSMUST00000112470 | 0.046163 | 1.222127 |
| A_52_P652671 | Hps1 | 0.0232 | 1.221846 |
| A_51_P286215 | Zfyve20 | 0.009746 | 1.22121 |
| A_51_P199974 | Slc4a8 | 0.030669 | 1.220741 |
| A_52_P141662 | Tcf3 | 0.009008 | 1.22057 |
| A_51_P460420 | Rsf1 | 0.007527 | 1.219174 |
| A_52_P188851 | Nefh | 0.001585 | 1.217746 |
| A_52_P284814 | Nup107 | 0.018476 | 1.217593 |
| A_51_P147651 | Ccdc15 | 0.010963 | 1.217054 |
| A_52_P190339 | 2610039C10Rik | 0.030902 | 1.217028 |
| A_51_P316616 | Smagp | 0.021817 | 1.216898 |
| A_51_P307858 | 1700019N19Rik | 0.029311 | 1.21555 |
| A_51_P476687 | Klrb1c | 0.012687 | 1.21532 |
| A_52_P963805 | AK053347 | 0.033138 | 1.214976 |
| A_51_P161115 | AK050756 | 0.02363 | 1.214837 |
| A_51_P428977 | Onecut1 | 0.018954 | 1.214617 |
| A_51_P229076 | Cd2ap | 0.017585 | 1.214521 |
| A_52_P536494 | Mycn | 0.039943 | 1.214505 |
| A_51_P147942 | Igf1 | 0.011682 | 1.213774 |
| A_51_P115749 | Usp42 | 0.017276 | 1.213733 |
| A_51_P208919 | Acbd6 | 0.007574 | 1.211692 |
| A_51_P498640 | Pdxk | 0.029546 | 1.210205 |
| A_51_P330677 | Slc35e1 | 0.030385 | 1.210079 |
| A_52_P137086 | C330007P06Rik | 0.014383 | 1.208548 |
| A_51_P487062 | Olfr1344 | 0.036545 | 1.208346 |
| A_52_P267600 | Mobp | 0.03176 | 1.20799 |
| A_51_P465161 | Dhdpsl | 0.042636 | 1.207242 |
| A_51_P223489 | AK040351 | 0.030848 | 1.206855 |
| A_51_P462546 | Gmppa | 0.039518 | 1.206832 |
| A_52_P54544 | Syt14 | 0.035293 | 1.206002 |
| A_52_P27482 | Rtkn2 | 0.018462 | 1.205141 |
| A_52_P600518 | Tsc22d3 | 0.026487 | 1.205093 |
| A_51_P398673 | C130039O16Rik | 0.048065 | 1.204942 |
| A_52_P189977 | Lrrc1 | 0.037218 | 1.204432 |
| A_51_P466270 | Heph | 0.027958 | 1.204021 |
| A_52_P268920 | 4933432B09Rik | 0.035788 | 1.20396 |
| A_51_P504314 | Prpf38b | 0.03767 | 1.203858 |
| A_51_P225535 | Asxl3 | 0.005811 | 1.203699 |
| A_52_P588483 | Fbln1 | 0.047504 | 1.203447 |
| A_52_P358612 | Sirt1 | 0.002114 | 1.202958 |
| A_51_P197213 | Pnpla2 | 0.037446 | 1.202486 |
| A_51_P115268 | Ankrd26 | 0.014253 | 1.20219 |
| A_51_P256693 | AK090157 | 0.036865 | 1.201749 |
| A_51_P146432 | Fam73b | 0.03898 | 1.199674 |
| A_51_P426466 | Rai2 | 0.046794 | 1.197796 |
| A_52_P418489 | Tinagl1 | 0.032327 | 1.197763 |
| A_51_P433157 | Chd9 | 0.020737 | 1.197612 |
| A_52_P534250 | Rbms3 | 0.049034 | 1.19688 |
| A_51_P310030 | Naca | 0.010361 | 1.196407 |
| A_51_P345699 | Tnnt3 | 0.030855 | 1.195386 |
| A_52_P620497 | 9630019E01Rik | 0.04183 | 1.195357 |
| A_52_P197007 | Axin1 | 0.040751 | 1.195015 |
| A_52_P434306 | ENSMUST00000110056 | 0.010147 | 1.193895 |
| A_52_P508163 | NAP021083-001 | 0.036765 | 1.193009 |
| A_51_P162307 | Phf3 | 0.029067 | 1.192712 |
| A_52_P405828 | AK042485 | 0.008231 | 1.192131 |
| A_51_P293310 | Hepacam | 0.005471 | 1.191869 |
| A_51_P411694 | Carns1 | 0.005021 | 1.190348 |
| A_52_P452315 | Syne2 | 0.003592 | 1.189505 |
| A_52_P557360 | Mtus2 | 0.013863 | 1.189382 |
| A_52_P580145 | Zfp526 | 0.023181 | 1.18937 |
| A_51_P136303 | Cyp4f15 | 0.01357 | 1.18769 |
| A_51_P215438 | Prodh | 0.024693 | 1.187471 |
| A_51_P419389 | Bmpr2 | 0.03388 | 1.186742 |
| A_51_P412160 | Rhbdf2 | 0.047953 | 1.186161 |
| A_52_P258606 | Prdx6 | 0.047979 | 1.18511 |
| A_51_P496795 | A330049M08Rik | 0.033245 | 1.184987 |
| A_51_P409429 | Aars | 0.002079 | 1.184644 |
| A_52_P219233 | Loxl3 | 0.041123 | 1.18457 |
| A_52_P397498 | Fbxo41 | 0.007496 | 1.1845 |
| A_51_P370438 | Lpin2 | 0.002143 | 1.182681 |
| A_51_P323248 | Sdc4 | 0.040456 | 1.181353 |
| A_51_P104757 | Hcfc1 | 0.046775 | 1.181208 |
| A_51_P368313 | Vip | 0.035834 | 1.181025 |
| A_52_P190647 | Mxd3 | 0.002362 | 1.180659 |
| A_52_P577624 | Dnajc28 | 0.004202 | 1.179741 |
| A_52_P655120 | Grik2 | 0.040717 | 1.179459 |
| A_51_P391367 | Tcfap4 | 0.023739 | 1.178822 |
| A_51_P119900 | Chadl | 0.005823 | 1.176214 |
| A_51_P215475 | Ptprb | 0.002149 | 1.175756 |
| A_52_P491849 | Trp53 | 0.044121 | 1.175651 |
| A_51_P218814 | Rpl22l1 | 0.01144 | 1.175175 |
| A_52_P629487 | Selenbp1 | 0.017794 | 1.174992 |
| A_52_P529751 | CF747846 | 0.014719 | 1.174773 |
| A_51_P259358 | Csrp2bp | 0.017336 | 1.172952 |
| A_52_P494622 | Nr4a2 | 0.031647 | 1.172804 |
| A_52_P57416 | Wdr59 | 0.045739 | 1.172471 |
| A_51_P231184 | Anpep | 0.010864 | 1.171984 |
| A_51_P287986 | Clstn1 | 0.009676 | 1.171357 |
| A_51_P246924 | Tppp3 | 0.001566 | 1.171349 |
| A_51_P430423 | Ada | 0.04921 | 1.170652 |
| A_52_P146485 | Chd3 | 0.00809 | 1.170478 |
| A_52_P574214 | Rrp1b | 0.042372 | 1.169136 |
| A_51_P324161 | Ankrd26 | 0.036717 | 1.169059 |
| A_52_P193440 | E130102H24Rik | 0.041797 | 1.167769 |
| A_51_P420547 | Clic5 | 0.013743 | 1.167325 |
| A_52_P297731 | Ube2i | 0.030937 | 1.166545 |
| A_51_P461504 | Eef2k | 0.026621 | 1.165983 |
| A_52_P358388 | Ppp4r2 | 0.039496 | 1.165626 |
| A_52_P653367 | Slc26a2 | 0.018725 | 1.165369 |
| A_52_P81980 | Cbara1 | 0.004941 | 1.164614 |
| A_51_P423356 | Katnal2 | 0.038802 | 1.164422 |
| A_51_P169617 | Taf3 | 0.009873 | 1.164308 |
| A_52_P552832 | Ndufa4 | 0.007226 | 1.164258 |
| A_51_P515623 | Qpctl | 0.025585 | 1.164073 |
| A_51_P153170 | Cyb5r3 | 0.046547 | 1.163869 |
| A_52_P559618 | Ifitm2 | 0.045579 | 1.163714 |
| A_52_P425651 | LOC100048534 | 0.01405 | 1.163048 |
| A_52_P475976 | Cep290 | 0.031149 | 1.162955 |
| A_52_P543460 | Pbx1 | 0.026225 | 1.161995 |
| A_52_P545255 | Cpsf2 | 0.032514 | 1.161947 |
| A_51_P404298 | Cox16 | 0.002057 | 1.161916 |
| A_52_P39644 | Nrxn3 | 0.033415 | 1.161482 |
| A_52_P328078 | Atp5b | 0.020126 | 1.161462 |
| A_51_P221802 | Tpr | 0.018244 | 1.160763 |
| A_52_P112203 | Spire1 | 0.00048 | 1.160742 |
| A_51_P224617 | Eea1 | 0.013775 | 1.160497 |
| A_51_P108020 | Zmynd15 | 0.036424 | 1.159993 |
| A_51_P490397 | Pold3 | 0.005362 | 1.159949 |
| A_52_P74619 | Pogz | 0.049122 | 1.159799 |
| A_52_P259658 | Mia3 | 0.014599 | 1.159487 |
| A_52_P68306 | Dlgap2 | 0.047733 | 1.158643 |
| A_52_P118591 | Tnfsf12-Tnfsf13 | 0.037474 | 1.158155 |
| A_51_P409988 | Arl13b | 0.012455 | 1.157889 |
| A_51_P291438 | Tnc | 0.048341 | 1.157862 |
| A_51_P406429 | Pdk1 | 0.01401 | 1.157261 |
| A_51_P388048 | Pcdha4 | 0.00461 | 1.156443 |
| A_52_P297009 | Itpk1 | 0.008896 | 1.156413 |
| A_51_P226922 | Pcsk1n | 0.040316 | 1.156055 |
| A_52_P205789 | Unc119b | 0.021389 | 1.155938 |
| A_51_P506284 | Pdlim7 | 0.012642 | 1.155717 |
| A_52_P570652 | Flywch1 | 0.043772 | 1.154287 |
| A_52_P212479 | Rims3 | 0.035143 | 1.153452 |
| A_51_P394524 | Rpl11 | 0.000898 | 1.153183 |
| A_52_P520583 | 1700084E18Rik | 0.000697 | 1.153177 |
| A_52_P489193 | Pdlim7 | 0.036253 | 1.152958 |
| A_51_P515348 | Pgs1 | 0.044915 | 1.152814 |
| A_52_P107571 | Rsrc1 | 0.04589 | 1.152635 |
| A_51_P442366 | Diap2 | 0.000531 | 1.152064 |
| A_51_P122425 | Ctf1 | 0.006498 | 1.151862 |
| A_52_P508369 | NAP037603-1 | 0.01064 | 1.151718 |
| A_52_P627727 | 2210409E12Rik | 0.042472 | 1.151555 |
| A_52_P147733 | 4930431P19Rik | 0.00526 | 1.151501 |
| A_51_P492676 | Sardh | 0.040153 | 1.151497 |
| A_52_P14881 | Myo9b | 0.043794 | 1.150345 |
| A_52_P189970 | Stc1 | 0.046967 | 1.150233 |
| A_51_P351286 | Pcsk7 | 0.019769 | 1.149762 |
| A_52_P142154 | Pcyt2 | 0.010118 | 1.149636 |
| A_51_P234330 | Rtkn | 0.007004 | 1.149135 |
| A_52_P600531 | Bahd1 | 0.013966 | 1.148852 |
| A_51_P314397 | Crip2 | 0.011268 | 1.14831 |
| A_51_P369392 | AK078618 | 0.025916 | 1.147487 |
| A_52_P539161 | Rdh11 | 0.013138 | 1.147348 |
| A_52_P13783 | 4930523C07Rik | 0.035252 | 1.147097 |
| A_51_P378158 | Bbs1 | 0.018271 | 1.146751 |
| A_52_P276302 | Tshz1 | 0.018608 | 1.146454 |
| A_51_P373112 | BC031781 | 0.023228 | 1.146327 |
| A_52_P519737 | Scml4 | 0.028846 | 1.14573 |
| A_51_P267634 | Orai2 | 0.002247 | 1.145682 |
| A_52_P191567 | Plcl1 | 0.030607 | 1.145663 |
| A_52_P248378 | Cry2 | 0.01256 | 1.145275 |
| A_52_P600750 | NAP056541-1 | 0.032686 | 1.144827 |
| A_51_P361492 | Pou2f1 | 0.028391 | 1.144425 |
| A_52_P370311 | Plbd2 | 0.009397 | 1.14411 |
| A_51_P279154 | Bex1 | 0.010865 | 1.143913 |
| A_52_P365316 | Hecw1 | 0.027374 | 1.143718 |
| A_51_P187507 | Acad10 | 0.049749 | 1.143429 |
| A_51_P165342 | Anxa2 | 0.042237 | 1.143212 |
| A_52_P244162 | Golph3 | 0.01593 | 1.142656 |
| A_51_P103533 | Lmtk2 | 0.029584 | 1.142479 |
| A_51_P260504 | Arhgef4 | 0.008187 | 1.1415 |
| A_52_P458790 | ENSMUST00000120818 | 0.029518 | 1.141449 |
| A_52_P58059 | Usp34 | 0.021235 | 1.140334 |
| A_51_P150195 | Mtmr3 | 0.041863 | 1.14002 |
| A_52_P547965 | Chd1 | 0.037652 | 1.139588 |
| A_52_P614582 | Ube4b | 0.026245 | 1.139442 |
| A_51_P282523 | Gpr124 | 0.03395 | 1.139147 |
| A_51_P152234 | Trip4 | 0.03138 | 1.138611 |
| A_51_P502068 | A_51_P502068 | 0.019282 | 1.138427 |
| A_51_P128499 | Dennd3 | 0.04498 | 1.138293 |
| A_51_P200291 | Golga3 | 0.021359 | 1.138029 |
| A_51_P129720 | Hectd3 | 0.029964 | 1.137592 |
| A_51_P441234 | Pcdh10 | 0.012513 | 1.137528 |
| A_51_P106294 | Gck | 0.014512 | 1.137126 |
| A_52_P518808 | LOC676546 | 0.035573 | 1.136764 |
| A_52_P661 | Plxna4 | 0.045489 | 1.136631 |
| A_52_P97206 | Usp47 | 0.024086 | 1.136598 |
| A_51_P404300 | Cox16 | 0.007701 | 1.136276 |
| A_51_P389447 | ORF19 | 0.033871 | 1.135984 |
| A_52_P139399 | Ncapd2 | 0.024123 | 1.135961 |
| A_52_P84384 | Flna | 0.005121 | 1.135449 |
| A_51_P431543 | 1700027L20Rik | 0.034937 | 1.135109 |
| A_52_P987461 | ENSMUST00000071067 | 0.04579 | 1.134799 |
| A_51_P298107 | Vit | 0.004927 | 1.134748 |
| A_51_P215217 | Ccdc65 | 0.049215 | 1.134603 |
| A_52_P456059 | Pfdn1 | 0.030481 | 1.134391 |
| A_52_P197223 | Sec14l1 | 0.042175 | 1.134285 |
| A_51_P161362 | Gnl2 | 0.040318 | 1.13405 |
| A_51_P236588 | 9430038I01Rik | 0.036872 | 1.133439 |
| A_52_P561584 | Srp68 | 0.025332 | 1.133342 |
| A_52_P147816 | ENSMUST00000113017 | 0.034035 | 1.133167 |
| A_51_P151897 | R3hcc1 | 0.014039 | 1.132895 |
| A_52_P681771 | Pnpla2 | 0.033446 | 1.132758 |
| A_52_P138895 | Qsox2 | 0.040507 | 1.132739 |
| A_52_P142496 | Herc1 | 0.040488 | 1.132461 |
| A_52_P356343 | Fam13a | 0.031284 | 1.132312 |
| A_52_P199747 | Zscan18 | 0.029384 | 1.132293 |
| A_52_P358349 | Mtdh | 0.00782 | 1.131928 |
| A_51_P114797 | Cbln2 | 0.025108 | 1.131868 |
| A_52_P50623 | Itk | 0.044392 | 1.131812 |
| A_51_P342707 | Pold2 | 0.023429 | 1.131489 |
| A_51_P248044 | Lamb2 | 0.027934 | 1.131376 |
| A_51_P265151 | Arhgef10 | 0.023597 | 1.131334 |
| A_51_P135618 | Dlk1 | 0.044112 | 1.130867 |
| A_51_P364153 | Obfc1 | 0.002357 | 1.130743 |
| A_52_P13802 | Cbfa2t3 | 0.046806 | 1.130502 |
| A_51_P161582 | Ddr1 | 0.012511 | 1.130115 |
| A_52_P495237 | Pcnt | 0.024554 | 1.129273 |
| A_51_P158598 | Scrn2 | 0.037305 | 1.129232 |
| A_51_P211980 | Rgs3 | 0.007475 | 1.129226 |
| A_51_P385415 | Egln2 | 0.047784 | 1.129172 |
| A_52_P442145 | Scn2a1 | 0.014598 | 1.129023 |
| A_51_P369690 | Ubxn11 | 0.024585 | 1.128967 |
| A_51_P437883 | Mzf1 | 0.044239 | 1.128705 |
| A_52_P641147 | Rhbdd2 | 0.037608 | 1.128633 |
| A_52_P503308 | 2700097O09Rik | 0.048387 | 1.12859 |
| A_51_P376765 | Nfx1 | 0.019071 | 1.128572 |
| A_51_P452768 | Cyp4f14 | 0.018618 | 1.128517 |
| A_52_P229770 | Akap13 | 0.03196 | 1.128287 |
| A_52_P622244 | 6030419C18Rik | 0.010551 | 1.128144 |
| A_52_P30803 | TC1610785 | 0.002464 | 1.127334 |
| A_52_P605500 | Mmp24 | 0.003095 | 1.12731 |
| A_52_P355075 | Rab40c | 0.015084 | 1.127233 |
| A_51_P411296 | Nup50 | 0.01396 | 1.127186 |
| A_52_P483269 | Slc22a15 | 0.013575 | 1.12699 |
| A_51_P212592 | Pamr1 | 0.0435 | 1.126547 |
| A_52_P551129 | Grm7 | 0.004397 | 1.125519 |
| A_51_P507280 | Patl1 | 0.049794 | 1.125425 |
| A_52_P309177 | Zhx1 | 0.026093 | 1.125266 |
| A_51_P403799 | Spnb3 | 0.048129 | 1.125135 |
| A_52_P106251 | Git2 | 0.02107 | 1.123396 |
| A_52_P185485 | Arpc4 | 0.02919 | 1.123357 |
| A_52_P492856 | Spnb2 | 0.013709 | 1.123319 |
| A_51_P251587 | Psmd9 | 0.039045 | 1.123003 |
| A_52_P493394 | Vps24 | 0.031198 | 1.122664 |
| A_51_P275679 | Rassf5 | 0.019279 | 1.122311 |
| A_52_P489778 | Ablim1 | 0.007044 | 1.121593 |
| A_52_P72839 | Sec23a | 0.006181 | 1.121232 |
| A_52_P518715 | Nsf | 0.040828 | 1.121036 |
| A_51_P331090 | Ift140 | 0.020082 | 1.120353 |
| A_52_P632669 | Pcdhac2 | 0.028283 | 1.12018 |
| A_52_P119510 | Inpp1 | 0.003169 | 1.119907 |
| A_52_P427024 | Ldlr | 0.03261 | 1.119225 |
| A_52_P448205 | ENSMUST00000084120 | 0.003423 | 1.119073 |
| A_52_P233701 | Cbln2 | 0.006847 | 1.119023 |
| A_52_P626770 | Ilf3 | 0.033659 | 1.118673 |
| A_52_P569023 | Sphkap | 0.015201 | 1.118538 |
| A_52_P164017 | Scamp4 | 0.01702 | 1.11846 |
| A_52_P653543 | Taf4a | 0.044974 | 1.117315 |
| A_52_P229320 | Rptor | 0.048392 | 1.116861 |
| A_52_P64601 | Msl1 | 0.036867 | 1.1168 |
| A_51_P272106 | Cirbp | 0.046755 | 1.116736 |
| A_52_P375323 | Slc9a9 | 0.00555 | 1.116452 |
| A_52_P320904 | AI987944 | 0.043261 | 1.116405 |
| A_51_P484200 | Lrrc56 | 0.032091 | 1.116127 |
| A_51_P477458 | Grid2 | 0.01516 | 1.116018 |
| A_51_P195237 | Ccdc75 | 0.011478 | 1.115527 |
| A_51_P272993 | Ntm | 0.015048 | 1.115313 |
| A_51_P270436 | Ficd | 0.014489 | 1.114676 |
| A_52_P153685 | Hecw2 | 0.017722 | 1.114664 |
| A_51_P407304 | ENSMUST00000134655 | 0.021794 | 1.114664 |
| A_52_P566406 | Xpc | 0.017641 | 1.114557 |
| A_52_P78123 | Dcaf12 | 0.036528 | 1.114359 |
| A_51_P466828 | Fam109a | 0.040849 | 1.114342 |
| A_51_P190791 | Dcp2 | 0.005776 | 1.114151 |
| A_52_P635271 | Btbd6 | 0.008635 | 1.114137 |
| A_52_P25193 | Tcp11l2 | 0.027495 | 1.11394 |
| A_51_P149946 | Pdhx | 0.047747 | 1.113782 |
| A_51_P110814 | Nudt9 | 0.011218 | 1.112857 |
| A_52_P481686 | Wtip | 0.042444 | 1.112688 |
| A_51_P458168 | Vegfb | 0.000593 | 1.112611 |
| A_52_P376183 | A830010M20Rik | 0.021241 | 1.112208 |
| A_52_P369581 | Atm | 0.023044 | 1.111932 |
| A_52_P35164 | Trp53i13 | 0.011824 | 1.111469 |
| A_51_P394833 | Tshz1 | 0.025805 | 1.111288 |
| A_52_P371718 | Tgs1 | 0.018812 | 1.11091 |
| A_51_P493886 | Gpt2 | 0.048649 | 1.110565 |
| A_51_P472292 | Igfbp7 | 0.03698 | 1.11051 |
| A_52_P627863 | Ssbp3 | 0.04283 | 1.110269 |
| A_52_P46447 | Crim1 | 0.045609 | 1.109986 |
| A_52_P74229 | ENSMUST00000107080 | 0.030714 | 1.109942 |
| A_52_P222021 | Ensa | 0.004304 | 1.109766 |
| A_52_P42231 | Nol3 | 0.002273 | 1.109719 |
| A_52_P44030 | Exoc3 | 0.004889 | 1.109401 |
| A_52_P20354 | Chchd2 | 0.00805 | 1.109282 |
| A_52_P465386 | NAP040605-1 | 0.043419 | 1.1091 |
| A_51_P303000 | Zbtb44 | 0.036436 | 1.108841 |
| A_52_P411331 | Vac14 | 0.033278 | 1.108786 |
| A_52_P252322 | Pdcd11 | 0.025997 | 1.108232 |
| A_51_P333111 | Aox1 | 0.024474 | 1.108201 |
| A_52_P653585 | Gnai1 | 0.020587 | 1.10815 |
| A_51_P113274 | Hps6 | 0.049884 | 1.108115 |
| A_51_P411297 | Nup50 | 0.003203 | 1.107551 |
| A_52_P638746 | Phf14 | 0.01853 | 1.107537 |
| A_51_P129108 | Atf6 | 0.025271 | 1.107343 |
| A_51_P111907 | Slc22a17 | 0.047048 | 1.107332 |
| A_52_P110913 | AK087420 | 0.040557 | 1.107112 |
| A_52_P11413 | Camsap1l1 | 0.028027 | 1.1071 |
| A_52_P70395 | Stk36 | 0.02703 | 1.106436 |
| A_51_P375201 | Plk3 | 0.038693 | 1.10628 |
| A_52_P427494 | Gpbp1l1 | 0.004187 | 1.106194 |
| A_51_P343787 | Morf4l1 | 0.006416 | 1.105833 |
| A_52_P381846 | Rasa3 | 0.008997 | 1.105828 |
| A_51_P179697 | Fam57b | 0.048533 | 1.105816 |
| A_52_P241676 | Slc9a3r2 | 0.045665 | 1.105802 |
| A_52_P7406 | Vps37a | 0.01174 | 1.105695 |
| A_52_P480939 | Tnpo1 | 0.03206 | 1.105324 |
| A_51_P109171 | Os9 | 0.019708 | 1.105301 |
| A_51_P355987 | Setd1a | 0.049716 | 1.105041 |
| A_52_P558163 | Secisbp2 | 0.033695 | 1.104926 |
| A_51_P152222 | Rnf220 | 0.019563 | 1.104387 |
| A_52_P446625 | AW555464 | 0.005933 | 1.104376 |
| A_52_P360308 | Osbpl3 | 0.034721 | 1.103987 |
| A_51_P385390 | Tmub1 | 0.022305 | 1.103934 |
| A_52_P352541 | Zfp389 | 0.020379 | 1.103902 |
| A_52_P587441 | Ctnnd2 | 0.005749 | 1.1038 |
| A_52_P173860 | BC046331 | 0.041018 | 1.10369 |
| A_52_P216525 | Adcy3 | 0.02425 | 1.103178 |
| A_51_P413348 | Ezr | 0.003537 | 1.102901 |
| A_51_P461828 | Tbp | 0.021376 | 1.102897 |
| A_51_P335702 | Tmem114 | 0.015809 | 1.102709 |
| A_51_P370163 | Ctr9 | 0.001458 | 1.102382 |
| A_51_P253279 | Cntn6 | 0.038784 | 1.102362 |
| A_51_P282518 | Efha2 | 0.029778 | 1.102249 |
| A_52_P228079 | Atf1 | 0.022032 | 1.102188 |
| A_51_P297552 | Med16 | 0.02801 | 1.101955 |
| A_51_P425284 | Aldoc | 0.036581 | 1.101785 |
| A_51_P113527 | Eif4a1 | 0.020958 | 1.101716 |
| A_51_P446558 | 1810026J23Rik | 0.01506 | 1.10166 |
| A_52_P665309 | Ankrd13a | 0.048974 | 1.101562 |
| A_52_P449328 | BC020419 | 0.031237 | 1.101374 |
| A_52_P321225 | Mapre1 | 0.040489 | 1.101347 |
| A_52_P582150 | Gosr2 | 0.040852 | 1.101061 |
| A_52_P920129 | Gnai1 | 0.030442 | 1.100564 |
| A_51_P210082 | Ercc4 | 0.027028 | 1.100239 |
| A_52_P328044 | Tle1 | 0.027743 | 1.100197 |
| A_52_P365342 | Lrrn2 | 0.030386 | 1.100149 |
| A_52_P213402 | Dhx34 | 0.01768 | 1.099548 |
| A_51_P243288 | Rpl34 | 0.040817 | 1.099338 |
| A_52_P467718 | Ap3m2 | 0.004154 | 1.099275 |
| A_51_P417725 | Pik3r2 | 0.025231 | 1.099221 |
| A_51_P468073 | Ggt1 | 0.049057 | 1.099118 |
| A_51_P221510 | Fam81a | 0.015498 | 1.098958 |
| A_51_P252328 | Dcaf10 | 0.040939 | 1.098713 |
| A_52_P516059 | Tia1 | 0.024863 | 1.098522 |
| A_52_P359718 | Wnk2 | 0.02493 | 1.09847 |
| A_51_P482951 | Fpgs | 0.036395 | 1.098255 |
| A_51_P174591 | Prdx5 | 0.029569 | 1.098183 |
| A_51_P422457 | Plcl1 | 0.030691 | 1.097974 |
| A_51_P293665 | Cxx1b | 0.04066 | 1.097879 |
| A_52_P447477 | Prepl | 0.039854 | 1.097674 |
| A_51_P155604 | Eif4ebp3 | 0.045097 | 1.097559 |
| A_51_P433733 | Nucb1 | 0.028532 | 1.097316 |
| A_52_P57013 | Nxn | 0.015869 | 1.096663 |
| A_51_P337210 | Lrrc4 | 0.022218 | 1.095915 |
| A_51_P462047 | Prpf38a | 0.004758 | 1.095476 |
| A_51_P505156 | Dgkz | 0.04422 | 1.095384 |
| A_51_P188602 | Orai3 | 0.01773 | 1.095328 |
| A_52_P619761 | Set | 0.006404 | 1.094812 |
| A_52_P105164 | Nudcd3 | 0.038438 | 1.094529 |
| A_51_P511448 | Sgsm1 | 0.024645 | 1.094508 |
| A_51_P466964 | Pgp | 0.040754 | 1.094447 |
| A_51_P390276 | Cwc25 | 0.034248 | 1.094384 |
| A_51_P443569 | Mum1 | 0.016281 | 1.094356 |
| A_51_P168665 | Prpf8 | 0.030821 | 1.094329 |
| A_51_P520802 | BC020535 | 0.043499 | 1.094209 |
| A_51_P103209 | Ttc7 | 0.047649 | 1.094077 |
| A_52_P375876 | Arf3 | 0.004609 | 1.093867 |
| A_52_P375598 | Rai1 | 0.014516 | 1.093824 |
| A_52_P352735 | Ccdc153 | 0.025445 | 1.093668 |
| A_51_P130332 | Hspa12b | 0.038779 | 1.093443 |
| A_51_P325914 | Jun | 0.030886 | 1.093343 |
| A_51_P152685 | Pcnxl2 | 0.008241 | 1.092824 |
| A_51_P426739 | Gpt | 0.031035 | 1.092777 |
| A_52_P138806 | Dlgap3 | 0.008443 | 1.092499 |
| A_52_P158923 | Mbd1 | 0.01189 | 1.0921 |
| A_51_P185135 | Gpr108 | 0.015228 | 1.092033 |
| A_51_P165870 | Nxn | 0.014245 | 1.09192 |
| A_51_P122085 | Usp20 | 0.000729 | 1.091624 |
| A_51_P249360 | Suox | 0.002808 | 1.0913 |
| A_52_P220347 | Ufd1l | 0.017231 | 1.090886 |
| A_52_P638513 | 2310061J03Rik | 0.00645 | 1.090258 |
| A_51_P431018 | Selenbp2 | 0.047619 | 1.090193 |
| A_51_P384618 | Dhdds | 0.005394 | 1.089893 |
| A_52_P193533 | Slc25a30 | 0.005746 | 1.089186 |
| A_52_P364970 | Chchd7 | 0.003351 | 1.08906 |
| A_51_P364894 | Tgs1 | 0.039686 | 1.089 |
| A_51_P127545 | Rps7 | 0.044617 | 1.08897 |
| A_51_P211152 | Phf14 | 0.047819 | 1.088726 |
| A_52_P498690 | Mdga2 | 0.020359 | 1.088504 |
| A_51_P132718 | 2310010J17Rik | 0.014743 | 1.088397 |
| A_51_P499940 | Hmgb2 | 0.025531 | 1.088379 |
| A_51_P389597 | Ins2 | 0.038207 | 1.088241 |
| A_52_P170054 | Ndufv3 | 0.040799 | 1.088031 |
| A_51_P475049 | Uchl1 | 0.012398 | 1.087771 |
| A_52_P265358 | Igsf1 | 0.016899 | 1.087634 |
| A_51_P390285 | Larp6 | 0.001863 | 1.087208 |
| A_51_P250358 | Prpf39 | 0.049014 | 1.086776 |
| A_52_P315030 | Gm7428 | 0.045687 | 1.086726 |
| A_52_P274591 | Slc25a29 | 0.049685 | 1.086023 |
| A_52_P214612 | ENSMUST00000097723 | 0.026722 | 1.085958 |
| A_51_P468240 | Dennd4a | 0.040474 | 1.085851 |
| A_52_P128068 | 9030409G11Rik | 0.047334 | 1.085553 |
| A_51_P463765 | Timp3 | 0.037108 | 1.085452 |
| A_51_P398621 | Cacnb4 | 0.01651 | 1.085394 |
| A_52_P462358 | Fam160a2 | 0.012391 | 1.085056 |
| A_52_P480425 | Parp3 | 0.03021 | 1.08461 |
| A_52_P455055 | Golgb1 | 0.042218 | 1.084489 |
| A_51_P272283 | Cmbl | 0.040427 | 1.084377 |
| A_52_P321325 | Zhx3 | 0.003793 | 1.083992 |
| A_51_P483908 | Dctn1 | 0.032735 | 1.083938 |
| A_51_P236829 | Zswim7 | 0.046954 | 1.083767 |
| A_52_P599055 | D14Ertd436e | 0.031715 | 1.083508 |
| A_51_P391996 | Pgd | 0.023859 | 1.083502 |
| A_51_P388826 | Gprasp1 | 0.00943 | 1.083458 |
| A_51_P286172 | Lzts2 | 0.046096 | 1.082918 |
| A_51_P472538 | Tmem134 | 0.03932 | 1.082611 |
| A_52_P123485 | Tcf25 | 0.00255 | 1.082577 |
| A_51_P109835 | Uqcrc1 | 0.038893 | 1.082514 |
| A_52_P247603 | Cdk17 | 0.048976 | 1.082292 |
| A_52_P678942 | Plxnb2 | 0.046484 | 1.081825 |
| A_52_P680827 | Abat | 0.034148 | 1.081652 |
| A_51_P201237 | Larp1b | 0.007864 | 1.0816 |
| A_51_P376501 | ENSMUST00000154193 | 0.028474 | 1.081588 |
| A_51_P360918 | Ehd3 | 0.038193 | 1.081178 |
| A_51_P139848 | Wbp5 | 0.012477 | 1.080851 |
| A_51_P355375 | Huwe1 | 0.003783 | 1.080008 |
| A_51_P114472 | Fbxo2 | 0.038443 | 1.079903 |
| A_51_P193270 | Mga | 0.018169 | 1.079802 |
| A_51_P216108 | Rps6kl1 | 0.011666 | 1.079365 |
| A_51_P249250 | Ubxn1 | 0.033075 | 1.079298 |
| A_51_P194224 | Zbtb49 | 0.044608 | 1.079203 |
| A_51_P462814 | Sars2 | 0.043782 | 1.079188 |
| A_52_P557378 | Slmo1 | 0.027643 | 1.079027 |
| A_51_P209280 | Rab31 | 0.007083 | 1.079005 |
| A_52_P182843 | Taf1 | 0.022995 | 1.077742 |
| A_51_P434776 | Jmjd8 | 0.023252 | 1.077517 |
| A_51_P295575 | Cutc | 0.038624 | 1.076556 |
| A_52_P745 | Acox3 | 0.02554 | 1.07602 |
| A_52_P98232 | Hira | 0.011703 | 1.075961 |
| A_51_P304219 | Fam113a | 0.018256 | 1.075829 |
| A_52_P280899 | Wdr33 | 0.049328 | 1.075763 |
| A_51_P292757 | Acbd4 | 0.026749 | 1.075474 |
| A_52_P627327 | Nav3 | 0.046041 | 1.074997 |
| A_51_P331870 | Rnf145 | 0.002089 | 1.074846 |
| A_52_P671525 | Zmat1 | 0.036719 | 1.074805 |
| A_51_P181851 | Eml3 | 0.009965 | 1.07454 |
| A_52_P422162 | Armc8 | 0.032218 | 1.074184 |
| A_52_P535255 | Syf2 | 0.019167 | 1.074009 |
| A_51_P349036 | Snapc4 | 0.017111 | 1.073661 |
| A_51_P199567 | Med26 | 0.016481 | 1.072762 |
| A_51_P359359 | 5430437P03Rik | 0.045103 | 1.072592 |
| A_51_P294288 | Rhbdd2 | 0.042338 | 1.072559 |
| A_52_P337478 | Snx19 | 0.013454 | 1.072351 |
| A_51_P209225 | Tada3 | 0.011643 | 1.072342 |
| A_51_P428464 | Cdyl2 | 0.002113 | 1.072091 |
| A_52_P209172 | Mrpl41 | 0.009987 | 1.071905 |
| A_51_P248962 | Stxbp5 | 0.042579 | 1.071578 |
| A_52_P475889 | Uba52 | 0.009379 | 1.07154 |
| A_52_P423587 | AK129341 | 0.047916 | 1.071531 |
| A_51_P125716 | Ogfr | 0.045843 | 1.071098 |
| A_51_P364099 | Tsr2 | 0.036724 | 1.071014 |
| A_51_P507172 | 2310022A10Rik | 0.007383 | 1.070936 |
| A_51_P514270 | Add2 | 0.011719 | 1.070848 |
| A_52_P665742 | Srebf2 | 0.00527 | 1.07051 |
| A_52_P276792 | Foxp1 | 0.038536 | 1.070401 |
| A_51_P501312 | Gm16515 | 0.044814 | 1.070315 |
| A_51_P477179 | Sirt3 | 0.026408 | 1.070172 |
| A_51_P412817 | Dnahc2 | 0.040897 | 1.070156 |
| A_51_P461884 | Abl2 | 0.014361 | 1.07008 |
| A_52_P307893 | Pip4k2b | 0.025662 | 1.069461 |
| A_51_P128148 | Chmp1a | 0.042947 | 1.069371 |
| A_51_P171200 | Golm1 | 0.031769 | 1.069199 |
| A_51_P310850 | Plekha6 | 0.036327 | 1.06913 |
| A_52_P305073 | Pias1 | 0.045411 | 1.068551 |
| A_52_P50107 | Set | 0.031699 | 1.068496 |
| A_51_P121359 | Zc3h18 | 0.035505 | 1.068423 |
| A_51_P276321 | Szt2 | 0.046663 | 1.068297 |
| A_52_P31641 | Tmem145 | 0.037519 | 1.068184 |
| A_52_P579448 | Oscp1 | 0.037145 | 1.068067 |
| A_51_P383369 | Odz3 | 0.046168 | 1.067791 |
| A_51_P173212 | Osbpl2 | 0.029115 | 1.067786 |
| A_51_P141932 | Anks1 | 0.010504 | 1.06776 |
| A_51_P163953 | Nsg2 | 0.019402 | 1.067294 |
| A_52_P533731 | NAP030571-1 | 0.029937 | 1.067124 |
| A_51_P400773 | Hdgfrp2 | 0.039168 | 1.066889 |
| A_51_P209697 | Atcay | 0.020191 | 1.066748 |
| A_51_P425680 | Ivd | 0.039916 | 1.066726 |
| A_52_P208471 | Madd | 0.038238 | 1.066348 |
| A_51_P356355 | Cds2 | 0.024098 | 1.066297 |
| A_52_P826021 | B3galt1 | 0.049847 | 1.06612 |
| A_51_P426283 | Dhx30 | 0.023737 | 1.06481 |
| A_51_P494006 | Rbm16 | 0.037711 | 1.064647 |
| A_51_P392705 | Xpc | 0.033135 | 1.064087 |
| A_52_P578562 | Slc41a1 | 0.029179 | 1.063934 |
| A_52_P463386 | Saps1 | 0.027353 | 1.063494 |
| A_52_P516097 | Leng9 | 0.03288 | 1.063241 |
| A_51_P416387 | Morf4l2 | 0.021742 | 1.063139 |
| A_51_P430973 | Paqr7 | 0.01739 | 1.063064 |
| A_51_P237766 | Nsfl1c | 0.015543 | 1.062779 |
| A_51_P139400 | Taf1 | 0.042288 | 1.062492 |
| A_52_P346556 | AK038627 | 0.028413 | 1.062068 |
| A_51_P401921 | Srebf1 | 0.011857 | 1.061775 |
| A_51_P381522 | Gtf2ird1 | 0.011582 | 1.061408 |
| A_52_P1182012 | Tceb2 | 0.042927 | 1.061393 |
| A_52_P102773 | Hk1 | 0.022458 | 1.061154 |
| A_51_P167322 | Ankrd17 | 0.023279 | 1.061069 |
| A_52_P605266 | Ptprd | 0.033567 | 1.061055 |
| A_51_P381150 | Phf15 | 0.011382 | 1.060654 |
| A_52_P212501 | Tmem59 | 0.024913 | 1.059924 |
| A_52_P468472 | App | 0.039996 | 1.05971 |
| A_51_P498023 | Scyl1 | 0.026575 | 1.058945 |
| A_52_P16841 | Alkbh8 | 0.004769 | 1.058309 |
| A_52_P536082 | Prpf4 | 0.028426 | 1.058173 |
| A_51_P373589 | ENSMUST00000045517 | 0.016025 | 1.057917 |
| A_52_P50959 | Cdc26 | 0.045699 | 1.057916 |
| A_52_P499879 | Gstz1 | 0.025337 | 1.057861 |
| A_52_P515247 | Arhgdib | 0.043969 | 1.057059 |
| A_51_P195506 | Csf1 | 0.036764 | 1.056383 |
| A_51_P212038 | Atp6v0e2 | 0.030083 | 1.056158 |
| A_52_P316413 | Tmem57 | 0.011973 | 1.055619 |
| A_51_P438514 | Haus7 | 0.037242 | 1.055443 |
| A_52_P149163 | Mark4 | 0.018042 | 1.054948 |
| A_52_P163978 | Psmd2 | 0.041844 | 1.054731 |
| A_51_P184773 | Wdr1 | 0.005879 | 1.054449 |
| A_52_P570266 | Psmb10 | 0.010775 | 1.054266 |
| A_51_P223144 | Add3 | 0.044748 | 1.053771 |
| A_52_P421249 | Habp4 | 0.012677 | 1.053526 |
| A_51_P101255 | Coasy | 0.035493 | 1.053477 |
| A_51_P118763 | Ahctf1 | 0.038221 | 1.053429 |
| A_52_P424784 | Clstn2 | 0.041266 | 1.053329 |
| A_51_P142923 | Chka | 0.020291 | 1.053137 |
| A_51_P226142 | Fam116a | 0.046268 | 1.052455 |
| A_51_P155085 | Dennd2a | 0.024997 | 1.052199 |
| A_52_P547234 | Tomm70a | 0.013455 | 1.052063 |
| A_51_P157554 | Brd7 | 0.039473 | 1.051774 |
| A_51_P481644 | Mbip | 0.030487 | 1.051699 |
| A_52_P24986 | Agpat6 | 0.014868 | 1.049953 |
| A_52_P489119 | Nol6 | 0.025651 | 1.049263 |
| A_51_P217236 | Derl1 | 0.047612 | 1.047112 |
| A_51_P325274 | Zkscan5 | 0.038241 | 1.046737 |
| A_52_P378056 | ENSMUST00000072583 | 0.011254 | 1.04657 |
| A_51_P173233 | Lysmd1 | 0.032578 | 1.045693 |
| A_51_P344046 | Nt5m | 0.014552 | 1.044454 |
| A_51_P459741 | Gprasp1 | 0.043763 | 1.044298 |
| A_51_P436002 | Hivep1 | 0.017075 | 1.041508 |
| A_51_P157255 | Sdc2 | 0.014372 | 1.041232 |
| A_51_P130660 | Mrps30 | 0.032632 | 1.039198 |
| A_51_P356303 | Stx8 | 0.042494 | 1.038449 |
| A_51_P230439 | Ppfibp2 | 0.045642 | 1.038416 |
| A_52_P576678 | A_52_P576678 | 0.049043 | 1.037757 |
| A_52_P265889 | Sap18 | 0.030481 | 1.037687 |
| A_52_P513281 | Wrnip1 | 0.040153 | 1.037024 |
| A_51_P122769 | Nfyb | 0.04658 | 1.031093 |
| A_51_P191669 | Chgb | 0.012189 | 1.030081 |
| A_52_P408338 | S100pbp | 0.025348 | 1.022844 |
| A_51_P284476 | Gpm6b | 0.034216 | 0.976169 |
| A_51_P245275 | H2afx | 0.013866 | 0.972409 |
| A_51_P127615 | Slbp | 0.034108 | 0.96673 |
| A_51_P469522 | Hs2st1 | 0.040999 | 0.966513 |
| A_51_P110931 | 1700022C21Rik | 0.047644 | 0.965537 |
| A_51_P274768 | Rps25 | 0.035848 | 0.964128 |
| A_52_P294675 | NAP027199-1 | 0.032691 | 0.964077 |
| A_51_P201520 | Cnot2 | 0.03438 | 0.961879 |
| A_52_P273748 | Zfp157 | 0.025018 | 0.960855 |
| A_52_P103260 | AA987161 | 0.041832 | 0.959791 |
| A_51_P136888 | Rb1 | 0.040189 | 0.959047 |
| A_51_P133037 | Ppp1r14b | 0.034486 | 0.958869 |
| A_51_P102652 | Mdp1 | 0.049067 | 0.957692 |
| A_51_P327904 | Yipf1 | 0.009973 | 0.95718 |
| A_51_P120257 | Uba2 | 0.003072 | 0.957115 |
| A_52_P526750 | Atg4d | 0.049385 | 0.956365 |
| A_52_P134382 | Rplp2 | 0.038493 | 0.956326 |
| A_51_P115829 | Tbcb | 0.029323 | 0.954937 |
| A_51_P112445 | Wdr3 | 0.042991 | 0.954548 |
| A_52_P373369 | Phc1 | 0.038827 | 0.954502 |
| A_51_P334571 | Uba52 | 0.000253 | 0.954426 |
| A_51_P300143 | Uqcr11 | 0.025557 | 0.954221 |
| A_51_P136456 | 2700060E02Rik | 0.017317 | 0.953169 |
| A_52_P601055 | Mtss1 | 0.035742 | 0.953006 |
| A_52_P40241 | Zfp746 | 0.042159 | 0.952475 |
| A_51_P243930 | Qrsl1 | 0.032468 | 0.950964 |
| A_51_P501963 | Prps1 | 0.040664 | 0.950566 |
| A_52_P427992 | Fibp | 0.049085 | 0.949618 |
| A_51_P142653 | Son | 0.014189 | 0.949455 |
| A_51_P223575 | Tbc1d23 | 0.037805 | 0.94871 |
| A_51_P115027 | Afg3l2 | 0.031851 | 0.948408 |
| A_51_P398579 | Zfp280d | 0.02443 | 0.948202 |
| A_51_P477676 | Mcph1 | 0.02617 | 0.947155 |
| A_52_P546228 | Mettl3 | 0.042526 | 0.946849 |
| A_52_P642501 | Ddx46 | 0.024346 | 0.946748 |
| A_52_P633752 | Apc | 0.011894 | 0.946402 |
| A_52_P207743 | NAP028864-1 | 0.012308 | 0.946395 |
| A_51_P267194 | Faim | 0.035795 | 0.946257 |
| A_51_P496715 | 4930444A02Rik | 0.047936 | 0.945966 |
| A_51_P323212 | Shisa9 | 0.010232 | 0.945893 |
| A_51_P372463 | Fxr1 | 0.014772 | 0.945027 |
| A_52_P635608 | NAP071064-1 | 0.047525 | 0.94481 |
| A_52_P295906 | Ammecr1l | 0.04519 | 0.944633 |
| A_51_P411225 | Wipf1 | 0.025586 | 0.944537 |
| A_51_P488039 | Stk16 | 0.034407 | 0.944418 |
| A_51_P394735 | Arhgef18 | 0.008385 | 0.944153 |
| A_51_P387334 | Uqcr10 | 0.006978 | 0.943884 |
| A_52_P608444 | Nfat5 | 0.038847 | 0.943737 |
| A_51_P475502 | Ndufa8 | 0.047853 | 0.943727 |
| A_51_P455632 | Stx16 | 0.03533 | 0.943653 |
| A_51_P384879 | Mdh2 | 0.015176 | 0.943304 |
| A_51_P468505 | 2810408M09Rik | 0.04567 | 0.942732 |
| A_52_P447985 | Pgpep1 | 0.037335 | 0.942509 |
| A_52_P201015 | NAP112575-1 | 0.025711 | 0.942123 |
| A_52_P466741 | Hax1 | 0.013446 | 0.942017 |
| A_51_P386999 | 2900056L01Rik | 0.013018 | 0.941039 |
| A_51_P492245 | Wdr61 | 0.039776 | 0.94091 |
| A_52_P208787 | B230217C12Rik | 0.021862 | 0.940729 |
| A_52_P402394 | Tmem121 | 0.044305 | 0.94072 |
| A_51_P251892 | Magoh | 0.034689 | 0.940425 |
| A_51_P323195 | Atp13a4 | 0.001635 | 0.940198 |
| A_52_P510986 | Etf1 | 0.039467 | 0.940154 |
| A_51_P179864 | Prkx | 0.032612 | 0.939602 |
| A_52_P664619 | Zfp472 | 0.015325 | 0.938943 |
| A_51_P374268 | Psenen | 0.047872 | 0.938939 |
| A_52_P456617 | Plekhm3 | 0.044734 | 0.938338 |
| A_51_P180108 | Arpc5l | 0.008783 | 0.938331 |
| A_52_P75415 | Atp5l | 0.046822 | 0.938168 |
| A_52_P161780 | ENSMUST00000112634 | 0.010317 | 0.937829 |
| A_51_P271591 | Arl10 | 0.045194 | 0.937776 |
| A_51_P197321 | Clta | 0.037173 | 0.93771 |
| A_51_P455027 | Ebna1bp2 | 0.024043 | 0.93766 |
| A_51_P370099 | Pdgfa | 0.001882 | 0.937287 |
| A_51_P402393 | Klhl8 | 0.019237 | 0.937259 |
| A_51_P374034 | Ccdc45 | 0.025084 | 0.937203 |
| A_52_P69955 | Tesc | 0.046872 | 0.936876 |
| A_51_P356871 | NAP057003-1 | 0.029951 | 0.936274 |
| A_51_P406638 | Rfc1 | 0.027882 | 0.93606 |
| A_51_P390387 | ENSMUST00000028350 | 0.000687 | 0.935948 |
| A_51_P273609 | Itpka | 0.032842 | 0.935946 |
| A_52_P239292 | AI597479 | 0.032791 | 0.935803 |
| A_52_P641892 | Ptplad1 | 0.028146 | 0.935732 |
| A_51_P419263 | Rnmt | 0.043526 | 0.935666 |
| A_52_P348522 | NAP061133-1 | 0.036845 | 0.935578 |
| A_52_P236642 | Bnip1 | 0.023144 | 0.935325 |
| A_51_P259861 | Mettl9 | 0.007508 | 0.935254 |
| A_51_P206346 | Reep3 | 0.041318 | 0.935103 |
| A_51_P399625 | Cnih | 0.014292 | 0.934969 |
| A_51_P256653 | Lmbr1 | 0.044518 | 0.934757 |
| A_51_P110471 | Ddah1 | 0.021954 | 0.934755 |
| A_51_P211491 | Gusb | 0.005406 | 0.93466 |
| A_51_P438619 | Sobp | 0.042204 | 0.934273 |
| A_51_P192654 | Atp5s | 0.034322 | 0.934063 |
| A_51_P402994 | Ddx3y | 0.019486 | 0.933999 |
| A_51_P394339 | Eif2b1 | 0.04003 | 0.933995 |
| A_51_P430055 | Tspan7 | 0.014857 | 0.933735 |
| A_52_P482830 | Pnkd | 5.87E-05 | 0.933374 |
| A_52_P323503 | NAP103789-1 | 0.019632 | 0.933133 |
| A_51_P176972 | Amigo2 | 0.024037 | 0.933087 |
| A_52_P337679 | Canx | 0.039598 | 0.932702 |
| A_51_P454152 | Ercc6 | 0.044905 | 0.932671 |
| A_51_P518586 | Emg1 | 0.036752 | 0.932603 |
| A_51_P354724 | Wdr83 | 0.04547 | 0.932461 |
| A_51_P437786 | Rbm28 | 0.01683 | 0.932325 |
| A_52_P350301 | Gm9763 | 0.012564 | 0.932077 |
| A_52_P286141 | Cisd2 | 0.038871 | 0.931957 |
| A_51_P280192 | 1810027O10Rik | 0.034434 | 0.931932 |
| A_52_P681456 | Rftn2 | 0.004374 | 0.931916 |
| A_52_P93354 | 4922501C03Rik | 0.040127 | 0.931571 |
| A_51_P127297 | Hsd11b1 | 0.038197 | 0.931279 |
| A_52_P60760 | Ate1 | 0.036269 | 0.93109 |
| A_52_P297270 | Fbxo33 | 0.029407 | 0.930731 |
| A_52_P612537 | Ss18l1 | 0.012685 | 0.930687 |
| A_52_P293076 | Fech | 0.039239 | 0.930685 |
| A_52_P314129 | Pkia | 0.029496 | 0.93045 |
| A_51_P478544 | A_51_P478544 | 0.039006 | 0.930364 |
| A_52_P2632 | Negr1 | 0.005089 | 0.930247 |
| A_51_P380401 | Cars2 | 0.02374 | 0.93021 |
| A_51_P362161 | Ubqln4 | 0.023828 | 0.930154 |
| A_51_P229536 | Nqo2 | 0.005952 | 0.930039 |
| A_51_P291347 | Odc1 | 0.021144 | 0.930034 |
| A_51_P133229 | Sulf2 | 0.033343 | 0.930001 |
| A_52_P685938 | 4732418C07Rik | 0.035085 | 0.929902 |
| A_52_P538470 | Spats2l | 0.005805 | 0.929709 |
| A_52_P636608 | Fam184b | 0.044373 | 0.929069 |
| A_52_P739568 | AK082480 | 0.023133 | 0.929038 |
| A_52_P334188 | Sfn | 0.037675 | 0.928974 |
| A_52_P36256 | TC1637880 | 0.024524 | 0.928906 |
| A_51_P169032 | 2810004N23Rik | 0.046558 | 0.928652 |
| A_51_P408644 | Eif2ak4 | 0.041417 | 0.928409 |
| A_52_P472799 | Ecd | 0.027081 | 0.928389 |
| A_51_P365952 | Trdmt1 | 0.035607 | 0.928241 |
| A_51_P335094 | ENSMUST00000113904 | 0.022924 | 0.928125 |
| A_51_P114910 | Cstb | 0.031586 | 0.927925 |
| A_51_P275989 | Ccdc107 | 0.012677 | 0.927873 |
| A_51_P390884 | Pacsin2 | 0.036116 | 0.927422 |
| A_51_P276142 | Gpr37l1 | 0.016894 | 0.92731 |
| A_52_P404005 | ENSMUST00000093326 | 0.02312 | 0.92728 |
| A_51_P425768 | 2610301B20Rik | 0.030314 | 0.927032 |
| A_51_P266949 | Tmem144 | 0.02463 | 0.926809 |
| A_51_P412338 | Vps41 | 0.004356 | 0.926443 |
| A_52_P171064 | Wnk1 | 0.004846 | 0.926253 |
| A_51_P150648 | Lamp1 | 0.004122 | 0.926216 |
| A_51_P518823 | Acsl6 | 0.024806 | 0.926151 |
| A_51_P398723 | Flt1 | 0.040358 | 0.926146 |
| A_51_P408172 | Col6a4 | 0.049545 | 0.925883 |
| A_51_P257541 | E130309F12Rik | 0.014005 | 0.925806 |
| A_51_P253732 | Il17rd | 0.04693 | 0.92579 |
| A_52_P76931 | Wdhd1 | 0.025804 | 0.925762 |
| A_51_P232778 | Ykt6 | 0.021834 | 0.925678 |
| A_51_P469261 | NAP057024-1 | 0.003225 | 0.92563 |
| A_51_P343556 | Cdv3 | 0.030205 | 0.925421 |
| A_52_P45797 | Rg9mtd2 | 0.047884 | 0.925359 |
| A_52_P478532 | Sfrs13a | 0.047385 | 0.925344 |
| A_51_P493720 | Tfam | 0.037343 | 0.925192 |
| A_52_P612165 | Brap | 0.044097 | 0.925087 |
| A_51_P160344 | Cenpv | 0.033035 | 0.925062 |
| A_52_P117334 | Thumpd3 | 0.040404 | 0.925059 |
| A_51_P453635 | Mdk | 0.048023 | 0.924827 |
| A_52_P41014 | 4933427D14Rik | 0.015502 | 0.924757 |
| A_51_P420918 | Ly6i | 0.042302 | 0.923976 |
| A_51_P423444 | Fez2 | 0.026755 | 0.923969 |
| A_52_P621418 | Gm4995 | 0.016264 | 0.92391 |
| A_52_P428446 | Tsn | 0.04485 | 0.92381 |
| A_51_P191743 | Pcdhb16 | 0.016054 | 0.923599 |
| A_51_P369636 | Hat1 | 0.04254 | 0.923444 |
| A_51_P338542 | Mrps23 | 0.022886 | 0.923349 |
| A_51_P124798 | Tmem192 | 0.010553 | 0.923301 |
| A_52_P607255 | Slc2a13 | 0.032713 | 0.923257 |
| A_52_P603635 | Ykt6 | 0.039926 | 0.923253 |
| A_52_P103391 | B4galt1 | 0.015971 | 0.923225 |
| A_51_P215922 | Casp6 | 0.03434 | 0.923173 |
| A_52_P303176 | 1810037I17Rik | 0.039484 | 0.923052 |
| A_51_P292073 | Haghl | 0.049294 | 0.922956 |
| A_52_P496142 | Col4a2 | 0.042024 | 0.922675 |
| A_51_P217336 | Scamp1 | 0.026949 | 0.92265 |
| A_52_P195809 | Bdh2 | 0.024248 | 0.922619 |
| A_52_P385736 | 2610017I09Rik | 0.033727 | 0.921961 |
| A_51_P153176 | Agfg1 | 0.028947 | 0.921768 |
| A_51_P201567 | Tial1 | 0.047371 | 0.921722 |
| A_52_P249588 | D1Bwg0212e | 0.045761 | 0.921705 |
| A_51_P148494 | Rnft1 | 0.038295 | 0.921677 |
| A_51_P323081 | Bhlhb9 | 0.047888 | 0.921503 |
| A_52_P229709 | Ube2d3 | 0.048122 | 0.921323 |
| A_51_P193000 | Bbs4 | 0.014897 | 0.921137 |
| A_52_P417859 | 4933439F18Rik | 0.017069 | 0.920814 |
| A_51_P135517 | Coch | 0.038682 | 0.92079 |
| A_51_P493700 | Cep350 | 0.035214 | 0.920717 |
| A_51_P206971 | Mnat1 | 0.043559 | 0.920639 |
| A_52_P399972 | Grpel1 | 0.011393 | 0.920488 |
| A_52_P467930 | Prdx6-ps2 | 0.032204 | 0.920104 |
| A_52_P635791 | Krtap4-6 | 0.048366 | 0.920047 |
| A_51_P376959 | Tmeff2 | 0.014834 | 0.920008 |
| A_51_P234853 | Sdhb | 0.027073 | 0.91997 |
| A_52_P40363 | Grb10 | 0.017904 | 0.919935 |
| A_51_P191586 | Arpc1a | 0.014293 | 0.919698 |
| A_51_P483639 | Prtg | 0.026024 | 0.919691 |
| A_51_P420901 | Srp54a | 0.040866 | 0.919313 |
| A_52_P500622 | 3110057O12Rik | 0.012444 | 0.919113 |
| A_52_P367681 | Sdhaf2 | 0.04682 | 0.919041 |
| A_52_P421698 | Cyp2u1 | 0.02919 | 0.919035 |
| A_52_P26216 | Numb | 0.024294 | 0.918765 |
| A_52_P467438 | Cd99l2 | 0.005489 | 0.918665 |
| A_52_P708886 | BB755555 | 0.029061 | 0.918663 |
| A_51_P411264 | Txndc16 | 0.022002 | 0.918631 |
| A_51_P361638 | Rps14 | 0.044387 | 0.918411 |
| A_51_P122867 | Syt16 | 0.049612 | 0.918399 |
| A_51_P152826 | Golt1b | 0.021168 | 0.91826 |
| A_51_P307168 | Ddah1 | 0.005988 | 0.917908 |
| A_52_P237102 | Dpy19l3 | 0.042917 | 0.917624 |
| A_51_P437538 | Gm5124 | 0.003703 | 0.917362 |
| A_52_P332081 | Negr1 | 0.034259 | 0.917306 |
| A_51_P341571 | Klhdc2 | 0.036989 | 0.917233 |
| A_52_P939541 | AK037972 | 0.046379 | 0.917153 |
| A_51_P130757 | Acer3 | 0.017974 | 0.917059 |
| A_51_P183275 | Sympk | 0.007563 | 0.917051 |
| A_52_P606679 | Tceb3 | 0.010774 | 0.917029 |
| A_51_P394484 | Mllt3 | 0.019903 | 0.916978 |
| A_51_P392291 | Pdk3 | 0.0237 | 0.916781 |
| A_52_P312563 | Rnmt | 0.015165 | 0.916512 |
| A_51_P179688 | 1810048J11Rik | 0.042688 | 0.916303 |
| A_52_P55893 | Baalc | 0.037762 | 0.916032 |
| A_51_P391668 | D8Ertd738e | 0.025097 | 0.915753 |
| A_51_P475076 | Vbp1 | 0.005345 | 0.915747 |
| A_52_P626247 | NAP001627-002 | 0.046619 | 0.915704 |
| A_51_P482801 | Asb3 | 0.016261 | 0.915349 |
| A_52_P158710 | Dcbld1 | 0.039721 | 0.915332 |
| A_51_P481159 | Cbr3 | 0.032126 | 0.915201 |
| A_51_P291764 | D030074E01Rik | 0.049216 | 0.915131 |
| A_52_P45170 | Mospd1 | 0.017363 | 0.91482 |
| A_51_P236864 | Parp8 | 0.022678 | 0.91458 |
| A_52_P676271 | Tuba4a | 0.000167 | 0.914293 |
| A_51_P106191 | Ranbp9 | 0.018607 | 0.914201 |
| A_52_P585652 | Fndc3b | 0.008253 | 0.914055 |
| A_51_P404067 | Med20 | 0.011237 | 0.914005 |
| A_52_P625640 | Trim9 | 0.03607 | 0.913652 |
| A_51_P163305 | Nudt5 | 0.001925 | 0.913563 |
| A_52_P620893 | Armc8 | 0.045243 | 0.91347 |
| A_51_P348183 | Tmem141 | 0.027309 | 0.913127 |
| A_51_P417016 | Srsf3 | 0.017052 | 0.91273 |
| A_52_P364386 | Fbxl19 | 0.033759 | 0.912469 |
| A_51_P480046 | Entpd5 | 0.001772 | 0.912382 |
| A_52_P601698 | Uxt | 0.009636 | 0.912336 |
| A_51_P514623 | Cd302 | 0.049975 | 0.91221 |
| A_51_P334604 | Wbp4 | 0.001035 | 0.912176 |
| A_51_P272123 | Ndufa10 | 0.023098 | 0.911937 |
| A_51_P268953 | Tmem64 | 0.038758 | 0.911932 |
| A_52_P406371 | Med14 | 0.035674 | 0.911824 |
| A_51_P322334 | Rheb | 0.035682 | 0.911755 |
| A_51_P218563 | Yrdc | 0.02118 | 0.910627 |
| A_51_P485651 | 3110052M02Rik | 0.013702 | 0.91045 |
| A_52_P586944 | Bmpr1b | 0.027583 | 0.910417 |
| A_51_P122246 | Creld2 | 0.015317 | 0.910307 |
| A_51_P247614 | Ncrna00086 | 0.008432 | 0.909458 |
| A_52_P625808 | Sf3b1 | 0.001234 | 0.909324 |
| A_52_P300424 | Derl1 | 0.049749 | 0.909255 |
| A_51_P130110 | Idh3b | 0.036889 | 0.909198 |
| A_51_P183197 | 2310002J15Rik | 0.044501 | 0.909169 |
| A_51_P221062 | Prkar2b | 0.032811 | 0.90902 |
| A_51_P106799 | Pparg | 0.026528 | 0.908872 |
| A_52_P237948 | Tm6sf1 | 0.032554 | 0.908788 |
| A_51_P351919 | Zfp454 | 0.020715 | 0.908715 |
| A_52_P314181 | AK040606 | 0.00284 | 0.908674 |
| A_52_P473045 | Usp37 | 0.044788 | 0.908378 |
| A_52_P988461 | AK082583 | 0.015174 | 0.90821 |
| A_51_P107934 | Pak7 | 0.009042 | 0.908043 |
| A_51_P316129 | Atg4a | 0.014531 | 0.907988 |
| A_52_P323305 | Wdr7 | 0.016646 | 0.907453 |
| A_52_P434038 | Lsm2 | 0.001178 | 0.907396 |
| A_52_P980224 | AK084027 | 0.022865 | 0.907046 |
| A_51_P456657 | Tgif2 | 0.031017 | 0.906482 |
| A_52_P126158 | Irgm1 | 0.023539 | 0.906475 |
| A_51_P241269 | Actg2 | 0.033959 | 0.906006 |
| A_51_P465988 | Ganc | 0.024176 | 0.905996 |
| A_52_P796682 | Ccne1 | 0.019177 | 0.905986 |
| A_52_P339543 | C1d | 0.026045 | 0.905962 |
| A_51_P164939 | Tmem150a | 0.001449 | 0.905865 |
| A_52_P88007 | Slc6a7 | 0.042142 | 0.905786 |
| A_52_P374642 | Dlat | 0.028557 | 0.90503 |
| A_51_P484753 | Tmem66 | 0.000262 | 0.904976 |
| A_51_P507664 | Slc25a18 | 0.02615 | 0.904801 |
| A_51_P260169 | Gstm5 | 0.010627 | 0.904761 |
| A_52_P612382 | Cdc25b | 0.027374 | 0.904731 |
| A_52_P503809 | Tuba1c | 0.027122 | 0.904452 |
| A_52_P70216 | Gm12942 | 0.043842 | 0.904446 |
| A_51_P258435 | Zdhhc3 | 0.010631 | 0.904037 |
| A_51_P160113 | Copb1 | 0.036128 | 0.903962 |
| A_52_P290544 | Ccnl1 | 0.01503 | 0.903795 |
| A_51_P460153 | Xrn2 | 0.004093 | 0.903397 |
| A_52_P426863 | C230052I12Rik | 0.026396 | 0.903162 |
| A_51_P253303 | Gucy1b3 | 0.019586 | 0.90313 |
| A_51_P224300 | Tm9sf3 | 0.014849 | 0.9031 |
| A_51_P486660 | Trafd1 | 0.03141 | 0.902886 |
| A_51_P180362 | Scarb2 | 0.008927 | 0.902101 |
| A_51_P390676 | Hnrpll | 0.048429 | 0.90186 |
| A_52_P134666 | Srpr | 0.045988 | 0.901171 |
| A_52_P550620 | Nsmce2 | 0.014194 | 0.901161 |
| A_52_P21986 | Fam69a | 0.026139 | 0.901084 |
| A_52_P322555 | Pex13 | 0.006067 | 0.901051 |
| A_51_P458428 | Pim2 | 0.009579 | 0.900794 |
| A_51_P365790 | AK043052 | 0.022773 | 0.900792 |
| A_51_P430082 | Tst | 0.017754 | 0.900696 |
| A_52_P789023 | Sarnp | 0.013249 | 0.90064 |
| A_51_P286665 | Rbl1 | 0.024468 | 0.900595 |
| A_51_P119597 | Ap3b1 | 0.031168 | 0.90022 |
| A_51_P352296 | Sfrp1 | 0.027623 | 0.900164 |
| A_52_P241819 | Usp46 | 0.016803 | 0.900151 |
| A_52_P566030 | Polr3k | 0.033037 | 0.900083 |
| A_52_P328714 | Fbxw2 | 0.014549 | 0.900028 |
| A_51_P342556 | Zfhx4 | 0.001886 | 0.899671 |
| A_51_P135832 | Fermt2 | 0.013109 | 0.899491 |
| A_51_P328645 | Mapk1ip1l | 0.014782 | 0.899397 |
| A_51_P418560 | Lnx2 | 0.011698 | 0.899124 |
| A_51_P404236 | Atp6v0b | 0.040039 | 0.898329 |
| A_52_P67983 | Lcmt2 | 0.041515 | 0.898313 |
| A_52_P110257 | Wdr83 | 0.022245 | 0.898286 |
| A_52_P296632 | Gm6742 | 0.036222 | 0.89817 |
| A_51_P192130 | Stk10 | 0.015259 | 0.897415 |
| A_51_P471386 | Cdh20 | 0.008618 | 0.897288 |
| A_51_P462444 | Tm2d3 | 0.007291 | 0.896896 |
| A_52_P148222 | Nrcam | 0.043211 | 0.896842 |
| A_51_P213641 | Ap2b1 | 0.00267 | 0.89645 |
| A_52_P126513 | Eif2c3 | 0.033061 | 0.896157 |
| A_51_P511176 | Triap1 | 0.021 | 0.895966 |
| A_52_P407668 | A_52_P407668 | 0.030916 | 0.895925 |
| A_52_P564962 | ENSMUST00000049836 | 0.027146 | 0.895754 |
| A_52_P198019 | Fuz | 0.046311 | 0.895749 |
| A_51_P456816 | Nlgn3 | 0.024711 | 0.895157 |
| A_51_P413461 | Parg | 0.023258 | 0.894995 |
| A_52_P568781 | Sec63 | 0.023529 | 0.894902 |
| A_51_P486150 | Ocln | 0.019336 | 0.894755 |
| A_52_P435569 | Amz2 | 0.035435 | 0.894735 |
| A_52_P106929 | Atp5f1 | 0.031031 | 0.894679 |
| A_51_P249824 | Smap1 | 0.014159 | 0.894644 |
| A_51_P505400 | 9630003H22Rik | 0.024624 | 0.894534 |
| A_52_P24783 | Pds5b | 0.014107 | 0.894532 |
| A_52_P641367 | Pppde2 | 0.047375 | 0.894522 |
| A_51_P317376 | Synpo | 0.032737 | 0.894484 |
| A_51_P143152 | Luzp1 | 0.008291 | 0.894362 |
| A_52_P87892 | Fam175b | 0.00981 | 0.894254 |
| A_52_P1108539 | AK086969 | 0.001598 | 0.894058 |
| A_52_P345616 | 2700078E11Rik | 0.046804 | 0.894051 |
| A_51_P386549 | Dync1i2 | 0.03636 | 0.893868 |
| A_51_P106373 | Sdhc | 0.047625 | 0.893823 |
| A_51_P474431 | Cdc25b | 0.049904 | 0.893577 |
| A_52_P580912 | Fbxo2 | 0.038446 | 0.893361 |
| A_51_P455866 | Elf5 | 0.02478 | 0.892921 |
| A_51_P225134 | Myh2 | 0.020685 | 0.892819 |
| A_51_P416613 | 1810058I24Rik | 0.030898 | 0.892737 |
| A_52_P505277 | Ubxn10 | 0.032945 | 0.892509 |
| A_51_P122238 | Uchl5 | 0.018298 | 0.89214 |
| A_52_P391098 | Crem | 0.012405 | 0.892058 |
| A_52_P289692 | Gm16372 | 0.024722 | 0.891907 |
| A_52_P368945 | Skap2 | 0.000931 | 0.891903 |
| A_52_P228932 | Gys1 | 0.046942 | 0.891657 |
| A_52_P557459 | Npas3 | 0.034509 | 0.891639 |
| A_51_P407786 | Ly6h | 0.006783 | 0.891617 |
| A_51_P481546 | Lass5 | 0.015813 | 0.891325 |
| A_52_P14526 | Zyg11b | 0.032545 | 0.891139 |
| A_52_P499453 | Myt1l | 0.005726 | 0.891044 |
| A_51_P366931 | Prc1 | 0.028317 | 0.890943 |
| A_51_P335916 | Mrpl46 | 0.016498 | 0.890648 |
| A_52_P24648 | Kdm2b | 0.032201 | 0.890624 |
| A_52_P197608 | NAP123493-1 | 0.032471 | 0.890434 |
| A_51_P174555 | Ptrh2 | 0.042134 | 0.890127 |
| A_51_P430327 | Gmpr2 | 0.001549 | 0.889918 |
| A_52_P279786 | Whsc1 | 0.004292 | 0.889839 |
| A_52_P438299 | Mfn1 | 0.003854 | 0.889814 |
| A_52_P96360 | NAP058766-1 | 0.010652 | 0.889249 |
| A_51_P398848 | Trim37 | 0.04943 | 0.888995 |
| A_52_P142191 | Aph1b | 0.004514 | 0.888421 |
| A_51_P322677 | Dusp14 | 0.025683 | 0.888255 |
| A_52_P537696 | Mettl8 | 0.013187 | 0.888078 |
| A_52_P91054 | NAP068771-1 | 0.005855 | 0.887977 |
| A_51_P119039 | Npy5r | 0.017845 | 0.887757 |
| A_52_P17369 | Ociad1 | 0.006653 | 0.88754 |
| A_51_P300602 | A_51_P300602 | 0.023107 | 0.887384 |
| A_52_P375803 | Tmem183a | 0.049995 | 0.886846 |
| A_51_P326483 | Pus3 | 0.001555 | 0.886841 |
| A_51_P386648 | Glod5 | 0.005115 | 0.886826 |
| A_51_P339356 | Rnf24 | 0.033429 | 0.886627 |
| A_51_P123262 | 6330527O06Rik | 0.021756 | 0.886594 |
| A_51_P284678 | Krcc1 | 0.001499 | 0.886265 |
| A_51_P329269 | AK041510 | 0.036239 | 0.886188 |
| A_51_P217047 | Gng10 | 0.042315 | 0.885876 |
| A_51_P193346 | ENSMUST00000086068 | 0.035656 | 0.885677 |
| A_51_P429308 | Neto2 | 0.04731 | 0.885539 |
| A_52_P40704 | Sap130 | 0.003731 | 0.885458 |
| A_52_P36293 | ENSMUST00000113229 | 0.020862 | 0.885451 |
| A_51_P135066 | Tmem106b | 0.046294 | 0.885395 |
| A_51_P248304 | Vps26b | 0.012216 | 0.88535 |
| A_51_P185906 | Abi3 | 0.048161 | 0.885128 |
| A_51_P423030 | Mta1 | 0.023287 | 0.885111 |
| A_51_P435068 | Acadsb | 0.032711 | 0.884738 |
| A_51_P121506 | Ate1 | 0.031799 | 0.88471 |
| A_51_P395405 | Klhl5 | 0.030832 | 0.884444 |
| A_52_P637282 | Wipf1 | 0.016906 | 0.884235 |
| A_52_P723727 | AK086918 | 0.040795 | 0.883979 |
| A_51_P156363 | Hnrnpul1 | 0.006766 | 0.883919 |
| A_51_P456465 | Cldn10a | 0.030663 | 0.883722 |
| A_52_P395381 | Zfp282 | 0.04013 | 0.883414 |
| A_52_P279379 | Zcchc11 | 0.004566 | 0.883139 |
| A_52_P506147 | ENSMUST00000085433 | 0.033053 | 0.882919 |
| A_51_P356229 | Nxf1 | 0.036865 | 0.882791 |
| A_52_P200155 | Ppp2r5a | 0.035736 | 0.882351 |
| A_52_P1149594 | A_52_P1149594 | 0.048633 | 0.882236 |
| A_52_P537492 | Tmx2 | 0.017407 | 0.881997 |
| A_52_P571406 | Snx6 | 0.007623 | 0.88191 |
| A_52_P280469 | TC1688953 | 0.037928 | 0.881859 |
| A_51_P102943 | Hsf1 | 0.002661 | 0.881775 |
| A_51_P318401 | Stt3a | 0.014691 | 0.881472 |
| A_52_P176361 | Hist1h2bm | 0.026024 | 0.881444 |
| A_52_P295878 | A730094L24Rik | 0.023949 | 0.881104 |
| A_52_P900990 | CB321262 | 0.047431 | 0.880982 |
| A_51_P162984 | Prm1 | 0.035772 | 0.880594 |
| A_52_P390030 | NAP000001-074 | 0.039561 | 0.880432 |
| A_52_P404895 | Tmem62 | 0.022055 | 0.880378 |
| A_52_P50216 | Spin1 | 0.011573 | 0.8798 |
| A_52_P310225 | Dnaja1 | 0.02778 | 0.879779 |
| A_52_P596146 | AK136420 | 0.001558 | 0.879664 |
| A_52_P25141 | Tmem37 | 0.043494 | 0.8794 |
| A_52_P964088 | AK081376 | 0.024056 | 0.879122 |
| A_52_P345840 | NAP059333-1 | 0.021999 | 0.878731 |
| A_51_P210072 | Smu1 | 0.033846 | 0.878572 |
| A_51_P297068 | Tmod1 | 0.018472 | 0.878469 |
| A_52_P261020 | Orc4l | 0.005184 | 0.87812 |
| A_51_P351672 | AK081115 | 0.011324 | 0.878067 |
| A_51_P173224 | Kcnh8 | 0.01723 | 0.878058 |
| A_51_P284937 | Gfm1 | 0.010956 | 0.878012 |
| A_51_P108573 | Tmem62 | 0.026585 | 0.877953 |
| A_52_P1156034 | AK047993 | 0.000553 | 0.877667 |
| A_52_P480088 | Col27a1 | 0.045107 | 0.877665 |
| A_51_P221998 | Man1c1 | 0.015872 | 0.877592 |
| A_51_P232708 | Npff | 0.049954 | 0.877414 |
| A_51_P255757 | Bcl2l13 | 0.014804 | 0.877365 |
| A_51_P253227 | Fbxo16 | 0.046282 | 0.877255 |
| A_52_P196732 | Nek6 | 0.023259 | 0.877091 |
| A_52_P454815 | Fgf11 | 0.009863 | 0.877017 |
| A_52_P75220 | 3110021N24Rik | 0.023703 | 0.876935 |
| A_51_P476357 | Magt1 | 0.032097 | 0.876849 |
| A_51_P258829 | Tmod4 | 0.043239 | 0.876794 |
| A_51_P212491 | Pfkfb3 | 0.032248 | 0.876773 |
| A_51_P486190 | Pabpc2 | 0.037204 | 0.876643 |
| A_51_P330369 | Ssx2ip | 0.00198 | 0.876294 |
| A_52_P215387 | D030063E12 | 0.015025 | 0.876056 |
| A_52_P851214 | AK049981 | 0.011241 | 0.875674 |
| A_51_P423859 | Capn3 | 0.028397 | 0.875499 |
| A_52_P670812 | Snord123 | 0.015267 | 0.875152 |
| A_51_P109295 | Pcdhb3 | 0.017745 | 0.874947 |
| A_52_P167189 | Isca1 | 0.017063 | 0.874797 |
| A_52_P167500 | NAP020770-001 | 0.044195 | 0.874304 |
| A_52_P163515 | LOC630077 | 0.041732 | 0.874211 |
| A_51_P482552 | Vegfa | 0.023617 | 0.874112 |
| A_51_P455647 | Car2 | 0.006479 | 0.873987 |
| A_52_P360529 | Frmd4a | 0.016256 | 0.873742 |
| A_52_P321875 | Rpl31 | 0.036753 | 0.873648 |
| A_51_P350332 | Rbpms | 0.030914 | 0.873122 |
| A_52_P7937 | Ppap2a | 0.010595 | 0.872885 |
| A_51_P476127 | Spry3 | 0.039631 | 0.872831 |
| A_52_P1115713 | AK038604 | 0.017791 | 0.872779 |
| A_52_P463271 | Gmds | 0.021192 | 0.872735 |
| A_52_P981301 | A_52_P981301 | 0.012273 | 0.872497 |
| A_52_P627631 | Pim2 | 0.011169 | 0.872312 |
| A_52_P6944 | Paf1 | 0.044163 | 0.872296 |
| A_52_P732508 | A_52_P732508 | 0.023292 | 0.872139 |
| A_52_P186352 | TC1651685 | 0.010755 | 0.872046 |
| A_51_P518959 | 1110025P21 | 0.021296 | 0.87199 |
| A_51_P150530 | Tm2d1 | 0.017698 | 0.871385 |
| A_51_P416215 | Sirt6 | 0.04161 | 0.871292 |
| A_52_P258645 | NAP010554-001 | 0.025856 | 0.871018 |
| A_52_P272054 | Gm11658 | 0.004909 | 0.87033 |
| A_51_P418526 | Sfxn1 | 0.01534 | 0.87018 |
| A_51_P364600 | Fyb | 0.036013 | 0.870053 |
| A_52_P256322 | Wdsub1 | 0.036988 | 0.868504 |
| A_52_P600304 | Slc25a46 | 0.020769 | 0.868415 |
| A_51_P424569 | Tbc1d12 | 0.013853 | 0.868202 |
| A_51_P241159 | Rhot1 | 0.039999 | 0.868041 |
| A_52_P92398 | Bbs12 | 0.036191 | 0.867998 |
| A_52_P672647 | Ak5 | 0.04312 | 0.867946 |
| A_52_P326657 | Fam167b | 0.00525 | 0.86765 |
| A_51_P233534 | Adamts5 | 0.017832 | 0.867649 |
| A_52_P283501 | Zc3h7b | 0.032099 | 0.867518 |
| A_51_P157154 | Grin2d | 0.035777 | 0.867229 |
| A_52_P8324 | Tmem178 | 0.001911 | 0.866074 |
| A_51_P435027 | Tmem186 | 0.023159 | 0.866035 |
| A_51_P288522 | 0610010K06Rik | 0.036406 | 0.86601 |
| A_52_P680311 | Bap1 | 0.021507 | 0.865903 |
| A_51_P242767 | Cmtm4 | 0.004919 | 0.865245 |
| A_51_P100052 | Slitrk2 | 0.035004 | 0.865045 |
| A_52_P401579 | Tnpo2 | 0.044673 | 0.864728 |
| A_52_P542763 | Bzw1 | 0.038308 | 0.864544 |
| A_52_P527400 | Hist3h2bb-ps | 0.030482 | 0.864154 |
| A_52_P1092263 | AK048920 | 0.010304 | 0.864104 |
| A_52_P747196 | AK048779 | 0.023147 | 0.864057 |
| A_52_P279687 | Lrp8 | 0.042475 | 0.863844 |
| A_51_P374782 | Epn3 | 0.034858 | 0.863482 |
| A_52_P669212 | NAP026415-1 | 0.017751 | 0.863425 |
| A_52_P157880 | Gm1947 | 0.000565 | 0.863401 |
| A_51_P139096 | 1700122O11Rik | 0.024361 | 0.863317 |
| A_51_P174653 | Fam60a | 0.039199 | 0.863225 |
| A_52_P544760 | Cbx5 | 0.029613 | 0.863171 |
| A_51_P475523 | Brca1 | 0.035996 | 0.86309 |
| A_52_P781398 | Trp53 | 0.029718 | 0.86235 |
| A_52_P313607 | NAP108117-1 | 0.017332 | 0.862327 |
| A_52_P236233 | Gast | 0.018691 | 0.862121 |
| A_52_P619903 | Tmem216 | 0.031414 | 0.861875 |
| A_52_P426062 | ENSMUST00000073410 | 0.015467 | 0.861812 |
| A_52_P16472 | Fam60a | 0.031154 | 0.861659 |
| A_52_P143174 | Gabrg2 | 0.026497 | 0.861442 |
| A_51_P272728 | Tmem165 | 0.043389 | 0.861308 |
| A_52_P455428 | Glra2 | 0.047908 | 0.861223 |
| A_52_P285635 | Elmod1 | 0.026076 | 0.861044 |
| A_52_P617888 | LOC638038 | 0.01874 | 0.860939 |
| A_52_P608460 | A230051G13Rik | 0.007841 | 0.860761 |
| A_51_P299475 | Olfr63 | 0.048691 | 0.86047 |
| A_52_P615096 | Acox1 | 0.041268 | 0.860328 |
| A_51_P134574 | Col4a3bp | 0.002636 | 0.859918 |
| A_51_P350048 | Gstt2 | 0.048578 | 0.859755 |
| A_52_P542496 | Slco1a5 | 0.025529 | 0.859719 |
| A_52_P389484 | Pag1 | 0.047601 | 0.859683 |
| A_52_P35470 | AK042543 | 0.049007 | 0.859635 |
| A_51_P476481 | Cyp2j13 | 0.042164 | 0.859616 |
| A_52_P482689 | Scube1 | 0.048327 | 0.85938 |
| A_52_P422675 | Hook3 | 0.005292 | 0.859371 |
| A_51_P426373 | Cib4 | 0.028103 | 0.858931 |
| A_52_P298373 | ENSMUST00000068394 | 0.01981 | 0.858864 |
| A_51_P259296 | Lpl | 0.044966 | 0.858638 |
| A_51_P167097 | AK038845 | 0.046101 | 0.858628 |
| A_52_P244895 | ENSMUST00000069456 | 0.011568 | 0.85853 |
| A_52_P161526 | Cdc26 | 0.04844 | 0.858465 |
| A_52_P220485 | Uhrf2 | 0.014715 | 0.85842 |
| A_52_P393120 | 1810012P15Rik | 0.039284 | 0.858397 |
| A_51_P458778 | Hpgd | 0.008019 | 0.858391 |
| A_51_P195932 | Tacr1 | 0.049661 | 0.858237 |
| A_52_P194500 | TC1765952 | 0.022719 | 0.858122 |
| A_51_P249821 | Smap1 | 0.015722 | 0.858117 |
| A_51_P279100 | Ptgs1 | 0.041027 | 0.857856 |
| A_51_P120470 | Cd68 | 0.049146 | 0.857834 |
| A_52_P426416 | Gnal | 0.034883 | 0.85731 |
| A_52_P464524 | 9630050P21Rik | 0.002252 | 0.857256 |
| A_52_P522397 | Tmem44 | 0.013143 | 0.857248 |
| A_52_P499551 | Celf2 | 0.005 | 0.856445 |
| A_51_P223686 | Lmo2 | 0.014805 | 0.856374 |
| A_52_P254286 | Krtap3-1 | 0.023847 | 0.856211 |
| A_52_P199928 | ENSMUST00000113617 | 0.036094 | 0.856195 |
| A_51_P344734 | Rps6 | 0.002895 | 0.855903 |
| A_52_P279845 | ENSMUST00000070720 | 0.030423 | 0.855717 |
| A_51_P368743 | C630043F03Rik | 0.0449 | 0.854229 |
| A_52_P2259 | ENSMUST00000079151 | 0.042574 | 0.854082 |
| A_51_P356389 | A330106F07Rik | 0.049199 | 0.853885 |
| A_51_P457980 | NAP108447-1 | 0.035822 | 0.853784 |
| A_51_P511270 | Pou3f1 | 0.041286 | 0.853637 |
| A_52_P371401 | Sbf2 | 0.041857 | 0.853199 |
| A_51_P434995 | Srcin1 | 0.024491 | 0.853168 |
| A_52_P24280 | Abhd2 | 0.032397 | 0.852333 |
| A_51_P429212 | Npl | 0.004047 | 0.852281 |
| A_52_P1156957 | BB505010 | 0.008945 | 0.851834 |
| A_52_P168549 | Fgf14 | 0.024165 | 0.851641 |
| A_51_P215380 | AK086300 | 0.004977 | 0.850867 |
| A_52_P86116 | Serpinb1b | 0.011493 | 0.850855 |
| A_51_P331638 | Onecut3 | 0.030344 | 0.850764 |
| A_52_P487647 | Hsf1 | 0.046916 | 0.850176 |
| A_51_P293715 | Fgfrl1 | 0.035913 | 0.849552 |
| A_52_P436524 | Sytl5 | 0.049713 | 0.848736 |
| A_52_P218609 | NAP036221-1 | 0.007464 | 0.848661 |
| A_51_P504602 | Map4k3 | 0.038 | 0.848156 |
| A_52_P393314 | P2rx7 | 0.03694 | 0.847858 |
| A_52_P54906 | NAP028254-1 | 0.044593 | 0.847448 |
| A_52_P17446 | TC1662444 | 0.013492 | 0.846823 |
| A_51_P150598 | Tsen2 | 0.040625 | 0.846408 |
| A_52_P461166 | NAP101988-1 | 0.010078 | 0.846124 |
| A_52_P58404 | Cacna1a | 0.042293 | 0.84557 |
| A_51_P328613 | Fcgr3 | 0.047846 | 0.84553 |
| A_51_P193316 | AK038374 | 0.006189 | 0.845408 |
| A_52_P588539 | Snapin | 0.005849 | 0.845367 |
| A_51_P385884 | Kcnu1 | 0.010356 | 0.84482 |
| A_51_P397363 | Slc16a1 | 0.040395 | 0.84479 |
| A_52_P33453 | D13Mgi7 | 0.000123 | 0.844585 |
| A_51_P181297 | Serpinb1a | 0.04371 | 0.844537 |
| A_52_P42380 | Tmem106c | 0.004203 | 0.844421 |
| A_52_P605296 | Wdr26 | 0.012968 | 0.844149 |
| A_52_P460929 | BC048507 | 0.012224 | 0.843819 |
| A_52_P603129 | Nlrc3 | 0.042104 | 0.84296 |
| A_51_P314153 | Nr2c2ap | 0.003987 | 0.842753 |
| A_52_P58041 | Arpc5 | 0.021994 | 0.842515 |
| A_52_P91757 | Bcorl1 | 0.022497 | 0.842498 |
| A_51_P411609 | ENSMUST00000049869 | 0.047479 | 0.842184 |
| A_52_P498396 | Neil2 | 0.017493 | 0.842127 |
| A_52_P613528 | Clec4b1 | 0.040477 | 0.841988 |
| A_52_P90444 | Ppp3r1 | 0.036197 | 0.841266 |
| A_52_P236398 | LOC100045515 | 0.040255 | 0.840895 |
| A_52_P683836 | TC1655871 | 0.022348 | 0.840683 |
| A_52_P661327 | Phyhipl | 0.022715 | 0.840472 |
| A_52_P411376 | Sptlc3 | 0.011997 | 0.840416 |
| A_51_P290974 | Bcr | 0.009063 | 0.839789 |
| A_51_P187253 | Irf8 | 0.042825 | 0.839352 |
| A_52_P492643 | D19Bwg1357e | 0.025038 | 0.83917 |
| A_51_P374878 | D330045A20Rik | 0.038123 | 0.839071 |
| A_51_P403771 | S42506 | 0.034792 | 0.838852 |
| A_51_P155073 | Nudt14 | 0.043911 | 0.838609 |
| A_51_P390334 | 2900026A02Rik | 0.048967 | 0.838126 |
| A_52_P604303 | Ispd | 0.035803 | 0.837286 |
| A_52_P221198 | Rab11fip5 | 0.026114 | 0.837033 |
| A_51_P146320 | Sema4d | 0.016975 | 0.836899 |
| A_52_P476086 | Nxf3 | 0.034213 | 0.836795 |
| A_52_P779936 | ENSMUST00000118679 | 0.037442 | 0.836647 |
| A_52_P597775 | Gprc5a | 0.037644 | 0.836331 |
| A_51_P432563 | Tlr9 | 0.031523 | 0.836331 |
| A_52_P337740 | AK030611 | 0.038838 | 0.835307 |
| A_52_P16212 | 2610507B11Rik | 0.048156 | 0.835305 |
| A_52_P47121 | TC1717239 | 0.019764 | 0.834766 |
| A_52_P223814 | NAP026710-1 | 0.047501 | 0.833952 |
| A_52_P353698 | Clec4a2 | 0.037461 | 0.833852 |
| A_51_P453428 | Cdhr5 | 0.00921 | 0.833205 |
| A_51_P418662 | Snora3 | 0.04937 | 0.832809 |
| A_51_P116838 | Dct | 0.010562 | 0.832789 |
| A_52_P26047 | Olfr1387 | 0.023986 | 0.832534 |
| A_51_P413366 | Atrx | 0.047996 | 0.832428 |
| A_52_P182979 | Tcf12 | 0.044508 | 0.83224 |
| A_52_P387334 | Dcc | 0.029853 | 0.832226 |
| A_52_P101252 | ENSMUST00000124981 | 0.040828 | 0.83177 |
| A_51_P514351 | Xlr | 0.027103 | 0.831462 |
| A_51_P137688 | Pex1 | 0.037943 | 0.831167 |
| A_51_P245939 | Snhg1 | 0.044899 | 0.830668 |
| A_51_P256066 | Tiam2 | 0.003776 | 0.830232 |
| A_52_P20211 | TC1665419 | 0.013943 | 0.829816 |
| A_52_P395280 | Lrrc55 | 0.048882 | 0.829771 |
| A_52_P225207 | Bak1 | 0.023363 | 0.829438 |
| A_52_P1179890 | AK038886 | 0.014195 | 0.82843 |
| A_52_P282448 | D830012I24Rik | 0.047852 | 0.828056 |
| A_52_P570471 | Csf1r | 0.044699 | 0.827211 |
| A_52_P377111 | TC1691242 | 0.041285 | 0.827122 |
| A_52_P337700 | NAP070910-1 | 0.014875 | 0.827055 |
| A_51_P301173 | 6230400D17Rik | 0.046657 | 0.826549 |
| A_51_P343833 | Traf1 | 0.044326 | 0.825777 |
| A_52_P49643 | Nup62cl | 0.048518 | 0.825711 |
| A_52_P549985 | Asb13 | 0.001328 | 0.825653 |
| A_51_P179686 | 1810048J11Rik | 0.007177 | 0.825365 |
| A_52_P217100 | NAP113293-1 | 0.010688 | 0.825345 |
| A_51_P370810 | Htr2c | 0.005455 | 0.825095 |
| A_52_P23366 | Olfr234 | 0.017994 | 0.825078 |
| A_52_P323309 | NAP049879-1 | 0.028793 | 0.824482 |
| A_51_P113058 | Olfr1131 | 0.021444 | 0.824462 |
| A_52_P240164 | Adcy1 | 0.029664 | 0.824351 |
| A_52_P900107 | AK083452 | 0.004386 | 0.823982 |
| A_51_P247799 | Casp8 | 0.044234 | 0.823455 |
| A_52_P876207 | AK085773 | 0.014354 | 0.823406 |
| A_51_P467284 | Mc3r | 0.031331 | 0.82304 |
| A_51_P296866 | Msi1 | 0.020169 | 0.821827 |
| A_51_P152203 | Zfp386 | 0.003145 | 0.821713 |
| A_52_P403527 | Gm8109 | 0.012494 | 0.820704 |
| A_52_P266680 | Trpc4ap | 0.030183 | 0.820529 |
| A_51_P341736 | Mmp2 | 0.026544 | 0.820399 |
| A_52_P173766 | Ids | 0.008954 | 0.819647 |
| A_51_P366290 | 6430526N21Rik | 0.004179 | 0.819596 |
| A_52_P50063 | 9230105E10Rik | 0.025192 | 0.819546 |
| A_52_P486910 | TC1756779 | 0.020794 | 0.819327 |
| A_51_P510997 | Atp7b | 0.002471 | 0.818648 |
| A_51_P433615 | Klhl6 | 0.025504 | 0.818619 |
| A_52_P519324 | Gm11938 | 0.029688 | 0.817943 |
| A_51_P343613 | Ehd2 | 0.030978 | 0.817574 |
| A_51_P271803 | Nsun2 | 0.015429 | 0.816756 |
| A_52_P368057 | Slc7a11 | 0.021936 | 0.816576 |
| A_51_P406193 | Lhfpl5 | 0.004331 | 0.816556 |
| A_51_P331831 | Hvcn1 | 0.031005 | 0.816318 |
| A_52_P94983 | Pla2g5 | 0.024031 | 0.815834 |
| A_52_P136195 | Mycbp | 0.043092 | 0.815763 |
| A_52_P288495 | 2610301G19Rik | 0.000642 | 0.815632 |
| A_52_P405994 | Orai1 | 0.017858 | 0.815457 |
| A_52_P49712 | Hira | 0.034023 | 0.815214 |
| A_51_P285065 | Tollip | 0.043696 | 0.815139 |
| A_51_P265725 | NAP029533-1 | 0.007617 | 0.81424 |
| A_52_P82804 | Gnl3l | 0.018021 | 0.81422 |
| A_51_P305642 | Krtap5-1 | 0.01852 | 0.813949 |
| A_52_P170573 | TC1652123 | 0.041916 | 0.813272 |
| A_52_P50417 | 1700084J12Rik | 0.021142 | 0.81228 |
| A_51_P449878 | Abcc3 | 0.045285 | 0.812001 |
| A_52_P138110 | LOC100048251 | 0.037499 | 0.811872 |
| A_51_P256344 | Anxa4 | 0.029707 | 0.810881 |
| A_52_P492069 | NAP026468-1 | 0.012504 | 0.810574 |
| A_52_P455643 | 2810030E01Rik | 0.005012 | 0.810287 |
| A_52_P416014 | ENSMUST00000084123 | 0.043333 | 0.809044 |
| A_52_P341489 | Gm7120 | 0.021079 | 0.808482 |
| A_52_P35513 | Ddx19a | 0.002606 | 0.808262 |
| A_52_P189038 | Rbms1 | 0.014973 | 0.807667 |
| A_51_P329975 | Ninj2 | 0.001765 | 0.807575 |
| A_52_P65513 | Fhl3 | 0.009039 | 0.80744 |
| A_51_P157866 | Ccnk | 0.001061 | 0.80703 |
| A_52_P50305 | Ube2k | 0.038715 | 0.806739 |
| A_52_P472324 | Slpi | 0.026456 | 0.806506 |
| A_52_P415047 | Olfr229 | 0.033989 | 0.80629 |
| A_52_P61691 | Cd59b | 0.016582 | 0.806061 |
| A_52_P182866 | C230081A13Rik | 0.01592 | 0.80511 |
| A_52_P646515 | Clnk | 0.013289 | 0.804561 |
| A_52_P1069274 | BU512393 | 0.023781 | 0.804536 |
| A_52_P220275 | Gpr34 | 0.011819 | 0.804318 |
| A_52_P349467 | LOC100039011 | 0.027047 | 0.804117 |
| A_52_P90442 | Ppp3r1 | 0.019497 | 0.803392 |
| A_52_P552062 | Fgfr1 | 0.025657 | 0.80336 |
| A_52_P90747 | Ptprs | 0.01089 | 0.80317 |
| A_52_P47276 | Gm16530 | 0.049906 | 0.803066 |
| A_52_P478051 | Celf2 | 0.006872 | 0.803029 |
| A_52_P141739 | Lama3 | 0.011043 | 0.803026 |
| A_52_P628885 | Baalc | 0.032134 | 0.802753 |
| A_52_P555188 | Kndc1 | 0.006762 | 0.802474 |
| A_52_P436282 | AK041732 | 0.020061 | 0.802227 |
| A_52_P180310 | AK042645 | 0.046828 | 0.801913 |
| A_51_P472153 | Rnase1 | 0.032822 | 0.801701 |
| A_51_P324037 | A_51_P324037 | 0.039309 | 0.800976 |
| A_52_P430348 | Serpinb1b | 0.017762 | 0.800502 |
| A_52_P166735 | Zdbf2 | 0.012708 | 0.80042 |
| A_51_P101787 | Hapln1 | 0.001193 | 0.800079 |
| A_51_P289380 | Olfr1341 | 0.027731 | 0.799996 |
| A_51_P483436 | 4930404H21Rik | 0.014204 | 0.79987 |
| A_51_P174696 | Casq2 | 0.000716 | 0.799238 |
| A_51_P412438 | Sfmbt2 | 0.008324 | 0.798028 |
| A_51_P408343 | Ifi204 | 0.034571 | 0.797064 |
| A_52_P460977 | BC025933 | 0.008023 | 0.797043 |
| A_51_P416278 | Ccdc142 | 0.048627 | 0.796639 |
| A_51_P512047 | AK084258 | 0.033127 | 0.796625 |
| A_52_P537031 | Pik3r4 | 0.011578 | 0.796582 |
| A_51_P305350 | A_51_P305350 | 0.034753 | 0.796571 |
| A_52_P123693 | E130304I02Rik | 0.034841 | 0.796561 |
| A_51_P364121 | A930011O12Rik | 0.026393 | 0.795799 |
| A_52_P98100 | Dcxr | 0.004304 | 0.795555 |
| A_52_P315051 | TC1611910 | 0.014658 | 0.79534 |
| A_52_P317653 | Car1 | 0.011187 | 0.794932 |
| A_51_P520397 | Gm9802 | 0.015659 | 0.793995 |
| A_52_P221696 | Tex10 | 0.043119 | 0.793099 |
| A_52_P182987 | Tcf12 | 0.044245 | 0.792703 |
| A_52_P502999 | TC1735521 | 0.033605 | 0.792275 |
| A_51_P278070 | TC1632399 | 0.015366 | 0.792011 |
| A_52_P571537 | 3110039M20Rik | 0.017515 | 0.791691 |
| A_52_P668534 | Atpbd4 | 0.022638 | 0.789406 |
| A_51_P135491 | Cflar | 0.009412 | 0.788962 |
| A_51_P161207 | Rfx3 | 0.00467 | 0.786325 |
| A_52_P158476 | Prok2 | 0.006158 | 0.786026 |
| A_52_P356562 | NAP061492-1 | 5.13E-05 | 0.785956 |
| A_51_P233334 | Stc1 | 0.016937 | 0.785682 |
| A_52_P139438 | Fam150b | 0.022079 | 0.785407 |
| A_51_P281637 | Iah1 | 0.022512 | 0.785245 |
| A_51_P159711 | Car7 | 0.001819 | 0.78438 |
| A_51_P483108 | D930020B18Rik | 0.016782 | 0.784357 |
| A_52_P65237 | Zbtb7c | 0.043779 | 0.78387 |
| A_51_P275077 | Glp1r | 0.04298 | 0.783821 |
| A_51_P443514 | 1700001K19Rik | 0.009546 | 0.783133 |
| A_52_P441462 | Nudt6 | 0.010099 | 0.782687 |
| A_51_P468456 | S100a3 | 0.038338 | 0.782461 |
| A_51_P337675 | Cd53 | 0.036893 | 0.782134 |
| A_52_P456810 | AK053813 | 0.026078 | 0.781773 |
| A_52_P650473 | Gm7209 | 0.035492 | 0.780645 |
| A_52_P477269 | NAP113002-1 | 0.038956 | 0.7806 |
| A_51_P267949 | 1300002E11Rik | 0.026003 | 0.77882 |
| A_51_P433679 | 1600014C10Rik | 0.006287 | 0.778698 |
| A_52_P375156 | Scamp5 | 0.004832 | 0.778635 |
| A_51_P165602 | Lbx2 | 0.039265 | 0.778071 |
| A_51_P374476 | Hbb-b1 | 0.037061 | 0.777801 |
| A_51_P119055 | Ssb | 0.007966 | 0.777591 |
| A_52_P32437 | BC051212 | 0.046359 | 0.777533 |
| A_51_P422335 | Zfp420 | 0.004234 | 0.777106 |
| A_51_P327405 | Gbp8 | 0.008405 | 0.776889 |
| A_51_P425815 | AI835086 | 0.04728 | 0.776811 |
| A_52_P132612 | NAP027398-1 | 3.38E-05 | 0.776659 |
| A_51_P281673 | Krtap9-1 | 0.01469 | 0.776496 |
| A_52_P542419 | Csnk1e | 0.023622 | 0.776414 |
| A_52_P105913 | Tom1l2 | 0.008737 | 0.774778 |
| A_52_P596230 | Sfxn5 | 0.00928 | 0.774713 |
| A_52_P210424 | Cacna1a | 0.010668 | 0.774112 |
| A_51_P264114 | Galnt5 | 0.037236 | 0.773686 |
| A_51_P300787 | Trappc9 | 0.03055 | 0.773094 |
| A_51_P298802 | Bfsp2 | 0.048879 | 0.772817 |
| A_52_P487289 | NAP123705-1 | 0.022594 | 0.772329 |
| A_51_P187579 | M17518 | 0.009542 | 0.771451 |
| A_51_P469648 | 1700020A23Rik | 0.028096 | 0.769818 |
| A_52_P71624 | Olfr1410 | 0.044187 | 0.769041 |
| A_52_P522166 | TC1654900 | 0.03392 | 0.76879 |
| A_51_P487043 | A_51_P487043 | 0.043105 | 0.768518 |
| A_52_P150651 | Cog5 | 0.038907 | 0.767758 |
| A_52_P605129 | Ndor1 | 0.00341 | 0.767026 |
| A_52_P61076 | Qrfpr | 0.008865 | 0.766321 |
| A_52_P316283 | NAP113018-1 | 0.011426 | 0.765777 |
| A_51_P216303 | Mmp14 | 0.013839 | 0.76397 |
| A_52_P260346 | Hbb-b1 | 0.032193 | 0.763931 |
| A_51_P362104 | Enpp5 | 0.021694 | 0.763791 |
| A_52_P612868 | NAP061206-1 | 0.000274 | 0.762671 |
| A_51_P492346 | Tmem174 | 0.005197 | 0.761828 |
| A_52_P763332 | AK036293 | 0.023586 | 0.761725 |
| A_52_P568520 | NAP071006-1 | 0.003502 | 0.761605 |
| A_52_P119972 | Tmc6 | 0.013028 | 0.761324 |
| A_52_P216672 | Klk8 | 0.032234 | 0.761084 |
| A_52_P460285 | NAP058254-1 | 0.003062 | 0.760628 |
| A_51_P484329 | U07662 | 0.047804 | 0.760488 |
| A_52_P532973 | Pxn | 0.046492 | 0.759626 |
| A_52_P663446 | Slc7a11 | 0.015172 | 0.759435 |
| A_52_P64482 | Wdr33 | 0.003742 | 0.759403 |
| A_52_P414065 | Mgl2 | 0.038014 | 0.758927 |
| A_52_P1131944 | A130019P10Rik | 0.025845 | 0.758612 |
| A_52_P485654 | 1810010K12Rik | 0.046671 | 0.758484 |
| A_51_P430414 | Olfr745 | 0.010111 | 0.758268 |
| A_52_P74715 | NAP019282-001 | 0.003958 | 0.757881 |
| A_51_P197618 | Atad5 | 0.032635 | 0.756929 |
| A_52_P664853 | Csf2rb | 0.04913 | 0.756714 |
| A_52_P47150 | TC1675045 | 0.024199 | 0.755404 |
| A_52_P290944 | Mamdc4 | 0.00797 | 0.755169 |
| A_51_P136601 | Trim7 | 0.03152 | 0.753425 |
| A_52_P208681 | Hba-a2 | 0.007769 | 0.753406 |
| A_51_P351828 | Olfr656 | 0.02642 | 0.752882 |
| A_52_P623640 | Nuak1 | 0.032563 | 0.752537 |
| A_52_P629625 | Bend6 | 0.021691 | 0.751952 |
| A_52_P471046 | Ifnz | 0.018901 | 0.751635 |
| A_52_P420234 | BG083441 | 0.048427 | 0.750736 |
| A_51_P478688 | Samd10 | 0.012206 | 0.750509 |
| A_52_P147089 | BC038268 | 0.001418 | 0.750406 |
| A_51_P235553 | ENSMUST00000068360 | 0.014783 | 0.750223 |
| A_52_P201775 | AK037472 | 0.027393 | 0.748811 |
| A_52_P46211 | Agl | 0.005271 | 0.748253 |
| A_52_P260187 | Dusp23 | 0.008923 | 0.748132 |
| A_51_P215272 | Cacna1d | 0.00502 | 0.747877 |
| A_52_P366385 | Rnf123 | 0.035875 | 0.747681 |
| A_51_P419801 | D130062J21Rik | 0.046292 | 0.744386 |
| A_52_P141077 | Gm10540 | 0.042421 | 0.743814 |
| A_51_P237924 | Slc6a7 | 0.017691 | 0.743688 |
| A_52_P972159 | AK084173 | 0.027567 | 0.742831 |
| A_51_P182936 | ENSMUST00000112652 | 0.031037 | 0.742684 |
| A_52_P221067 | Serpina3j | 0.002071 | 0.740825 |
| A_51_P399143 | Rbp2 | 0.021169 | 0.74039 |
| A_52_P351205 | NAP070138-1 | 0.003318 | 0.738239 |
| A_51_P190671 | Coq10a | 0.019637 | 0.736738 |
| A_51_P468054 | Smyd1 | 0.003431 | 0.73628 |
| A_51_P254634 | AK046342 | 0.042619 | 0.735971 |
| A_52_P149801 | Pde4b | 0.002749 | 0.735805 |
| A_51_P323400 | Zfp59 | 0.008402 | 0.735072 |
| A_52_P462540 | NAP059611-1 | 0.004206 | 0.734404 |
| A_51_P433000 | LOC100044854 | 0.014463 | 0.733522 |
| A_52_P436318 | Rps25 | 0.003151 | 0.732949 |
| A_52_P380379 | Ucp3 | 0.029209 | 0.731894 |
| A_52_P482724 | Prok1 | 0.018148 | 0.731073 |
| A_52_P362772 | AK048349 | 0.014834 | 0.730911 |
| A_52_P580925 | TC1648970 | 0.035383 | 0.730253 |
| A_51_P427099 | Clcc1 | 0.046472 | 0.73 |
| A_51_P193925 | Mapk6 | 0.038438 | 0.729352 |
| A_52_P126070 | Cenpw | 0.031241 | 0.729063 |
| A_51_P432950 | Sstr1 | 0.007084 | 0.72884 |
| A_52_P218883 | NAP101638-1 | 0.022509 | 0.728822 |
| A_51_P316243 | Pgpep1l | 0.018071 | 0.728135 |
| A_51_P165500 | AK083594 | 0.035521 | 0.727896 |
| A_52_P245316 | Gm98 | 0.047029 | 0.726991 |
| A_51_P349546 | Cd109 | 0.005919 | 0.726833 |
| A_51_P199634 | Rgag4 | 0.047976 | 0.726396 |
| A_52_P379631 | Steap2 | 0.023462 | 0.725762 |
| A_52_P241612 | Aldoart2 | 0.001044 | 0.724946 |
| A_52_P467900 | NAP020493-001 | 0.04715 | 0.721602 |
| A_51_P139538 | 5330437I02Rik | 0.00489 | 0.72117 |
| A_51_P303308 | Hes2 | 0.049536 | 0.72081 |
| A_52_P272175 | Dusp15 | 0.037367 | 0.720184 |
| A_51_P127131 | AK084897 | 0.020168 | 0.719646 |
| A_52_P1107923 | AK046467 | 0.007958 | 0.719083 |
| A_52_P77655 | NAP123065-1 | 0.006836 | 0.71849 |
| A_52_P77695 | Fam189b | 0.042125 | 0.717516 |
| A_51_P453995 | Gm11961 | 0.000698 | 0.717047 |
| A_52_P494597 | Ppp2r1b | 0.049368 | 0.716245 |
| A_51_P365037 | Nub1 | 0.027075 | 0.716106 |
| A_51_P321057 | AK170323 | 0.046771 | 0.71498 |
| A_52_P594107 | Gm4821 | 0.000372 | 0.714327 |
| A_52_P160915 | AK140400 | 0.017053 | 0.714184 |
| A_52_P1068111 | Trip12 | 0.031594 | 0.713265 |
| A_52_P190394 | Gnas | 0.042737 | 0.712228 |
| A_51_P332416 | Gm5085 | 0.031767 | 0.712226 |
| A_51_P176104 | AK090149 | 0.016792 | 0.710685 |
| A_52_P564166 | Myom1 | 0.040041 | 0.709143 |
| A_51_P468055 | Smyd1 | 0.003433 | 0.707546 |
| A_51_P128696 | Slc16a8 | 0.042535 | 0.705997 |
| A_52_P493400 | LOC100044027 | 0.023306 | 0.705816 |
| A_51_P462870 | Mkl2 | 0.040954 | 0.703037 |
| A_51_P386280 | Olfr187 | 0.000291 | 0.702589 |
| A_51_P499615 | Cldn12 | 0.029991 | 0.702585 |
| A_51_P222261 | Ikzf3 | 0.009409 | 0.701685 |
| A_52_P377532 | Fads6 | 0.023104 | 0.700998 |
| A_51_P211770 | Dpy19l2 | 0.029765 | 0.700611 |
| A_52_P112591 | ENSMUST00000103740 | 0.02658 | 0.699851 |
| A_51_P301007 | Spata19 | 0.016247 | 0.698855 |
| A_51_P136680 | Krtap31-1 | 0.042597 | 0.698528 |
| A_51_P293278 | Tcte2 | 0.046286 | 0.698207 |
| A_51_P122075 | AK052606 | 0.020991 | 0.696332 |
| A_51_P429252 | Prok2 | 0.000585 | 0.696046 |
| A_51_P478498 | Olfr1026 | 0.025336 | 0.694034 |
| A_52_P475578 | B130020M22Rik | 0.017595 | 0.693764 |
| A_51_P280666 | Bend6 | 0.014657 | 0.692201 |
| A_51_P421418 | Hsd3b1 | 0.011533 | 0.691646 |
| A_52_P163021 | Slc17a8 | 0.011225 | 0.690102 |
| A_52_P811275 | AK040293 | 0.044591 | 0.688119 |
| A_51_P425944 | Kpna6 | 0.010891 | 0.685961 |
| A_52_P941407 | A_52_P941407 | 0.001098 | 0.685695 |
| A_51_P290074 | Fabp7 | 0.046388 | 0.685565 |
| A_52_P249514 | Ccl12 | 0.039713 | 0.685304 |
| A_51_P431785 | Myom2 | 0.041807 | 0.680171 |
| A_52_P250106 | AK048296 | 0.045696 | 0.678497 |
| A_52_P1083948 | AK039978 | 0.010111 | 0.677853 |
| A_51_P287225 | Cyp27b1 | 0.036435 | 0.677348 |
| A_52_P666615 | TC1723067 | 0.012344 | 0.677114 |
| A_52_P323975 | ENSMUST00000095222 | 0.042059 | 0.676274 |
| A_51_P203306 | Vmn1r216 | 0.032023 | 0.67295 |
| A_52_P82023 | Pitpnc1 | 0.046549 | 0.671861 |
| A_51_P444302 | AK039273 | 0.001046 | 0.671294 |
| A_52_P236709 | Sp7 | 0.033173 | 0.670249 |
| A_51_P463159 | Casp14 | 0.008083 | 0.668937 |
| A_52_P185615 | Ftsj1 | 0.03745 | 0.667922 |
| A_51_P360508 | Tmem143 | 0.004692 | 0.665457 |
| A_52_P115091 | NAP062461-1 | 0.040569 | 0.661506 |
| A_51_P424272 | Mt4 | 0.002002 | 0.657627 |
| A_52_P484000 | NAP027099-1 | 0.041246 | 0.656966 |
| A_52_P378937 | Gfm2 | 0.037621 | 0.655069 |
| A_51_P371078 | Smg5 | 0.02219 | 0.65306 |
| A_52_P507649 | B130065D12Rik | 0.048162 | 0.646952 |
| A_52_P189455 | Vps18 | 0.046835 | 0.645969 |
| A_51_P194609 | Prss34 | 0.042953 | 0.638288 |
| A_52_P373392 | Itga4 | 0.011492 | 0.634395 |
| A_51_P490070 | Xlr3c | 0.031337 | 0.632605 |
| A_52_P287739 | AK040677 | 0.040956 | 0.632073 |
| A_51_P268687 | Mc2r | 0.020325 | 0.62739 |
| A_51_P107302 | Cav1 | 0.017559 | 0.625182 |
| A_52_P540826 | Gm4636 | 0.024646 | 0.622353 |
| A_52_P617762 | Arhgap5 | 0.036273 | 0.622234 |
| A_51_P412705 | AK042960 | 0.028 | 0.622015 |
| A_52_P602669 | Serpinb6d | 0.031301 | 0.621582 |
| A_52_P361391 | Olfr1153 | 0.022361 | 0.618066 |
| A_52_P104107 | Zfp35 | 0.01756 | 0.616524 |
| A_51_P250161 | Pdzd11 | 0.042219 | 0.610663 |
| A_52_P1157511 | EH103393 | 0.001795 | 0.610097 |
| A_51_P230716 | ENSMUST00000103521 | 0.023998 | 0.598654 |
| A_52_P599624 | Pfas | 0.034987 | 0.595723 |
| A_52_P368434 | Zfp354a | 0.036504 | 0.595517 |
| A_51_P130895 | Olfr1459 | 0.039479 | 0.593687 |
| A_52_P1051766 | AK029197 | 0.016279 | 0.592557 |
| A_52_P33382 | Gm5226 | 0.012278 | 0.588298 |
| A_51_P136188 | Slc6a14 | 0.027015 | 0.587555 |
| A_52_P585224 | AK089047 | 0.007621 | 0.58558 |
| A_52_P219393 | Gnas | 0.006029 | 0.585571 |
| A_52_P508827 | Lhx8 | 0.03001 | 0.58261 |
| A_52_P459900 | AK136065 | 0.036599 | 0.581331 |
| A_51_P480427 | Olfr430 | 0.007814 | 0.580563 |
| A_52_P610277 | TC1688754 | 0.029857 | 0.577608 |
| A_51_P506733 | P2rx7 | 0.000895 | 0.577314 |
| A_51_P463562 | Gbp4 | 0.04399 | 0.575654 |
| A_52_P290673 | AK082409 | 0.03455 | 0.568052 |
| A_51_P455128 | Wdr69 | 0.023628 | 0.566782 |
| A_52_P244797 | Klrc2 | 0.045372 | 0.554924 |
| A_52_P229705 | Acot6 | 0.031801 | 0.543914 |
| A_51_P116289 | Fam54a | 0.03631 | 0.533532 |
| A_52_P152240 | Cep120 | 0.016203 | 0.525783 |
| A_52_P571419 | ENSMUST00000103426 | 0.024942 | 0.525453 |
| A_51_P468005 | Fmo5 | 0.030014 | 0.519344 |
| A_51_P464874 | 9.13E+15 | 0.000274 | 0.51569 |
| A_52_P29615 | Rab27a | 0.030879 | 0.512012 |
| A_52_P448510 | Katnb1 | 0.021947 | 0.509837 |
| A_52_P426740 | Rab27a | 0.006544 | 0.493025 |
| A_52_P682293 | Hcn1 | 0.046611 | 0.461778 |
| A_52_P494207 | Itpr2 | 0.03729 | 0.431732 |
| A_51_P307872 | Cyp2j5 | 0.003695 | 0.400719 |
