# Supplementary figures and images for: Chronic alcohol consumption from adolescence-to-adulthood in mice - hypothalamic gene expression changes in the dilated cardiomyopathy signaling pathway
Source: BMC Neurosci. 2014 May 9;15:61. doi: 10.1186/1471-2202-15-61 (PMC4027996; doi:10.1186/1471-2202-15-61)

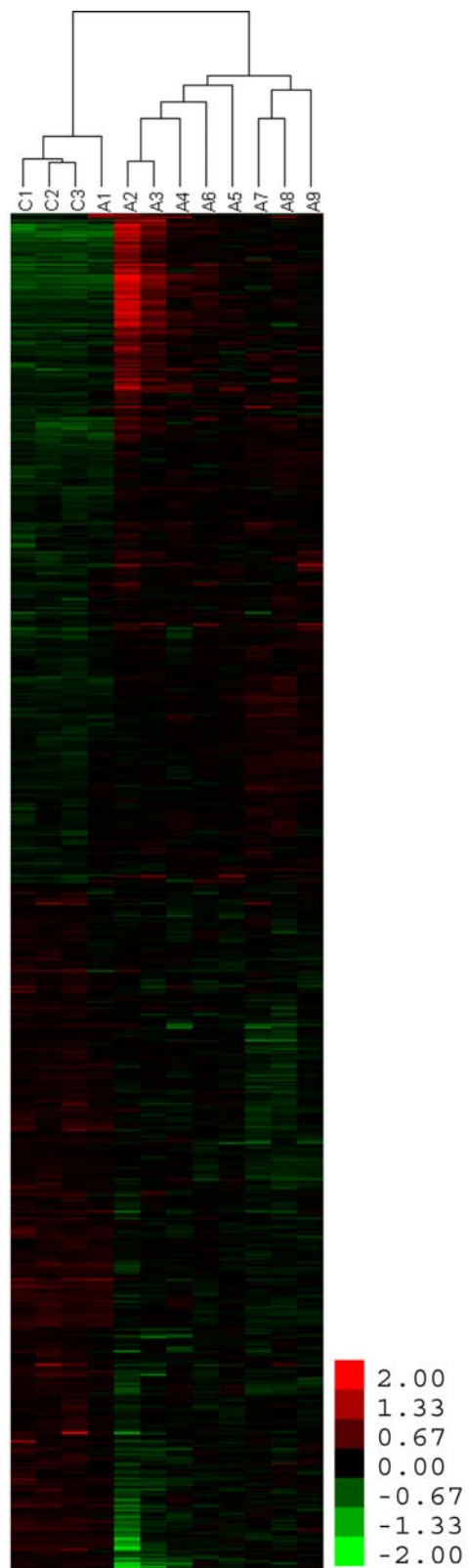

Figure S1

Supplement: Additional file 2: Figure S1 — Heat map of genes differentially expressed between chronic alcohol vs. control mouse hypothalamus samples. Gene expression values are color-coded according to the scale on the right. Displayed on top is the clustering display of differentially expressed genes using the unsupervised hierarchical clustering method. C1-C3: water-only control samples; A1-A9: chronic alcohol samples. [file 1471-2202-15-61-S2.pdf]
