# Supplementary material for: Chronic alcohol consumption from adolescence-to-adulthood in mice - hypothalamic gene expression changes in the dilated cardiomyopathy signaling pathway
Source: BMC Neurosci. 2014 May 9;15:61. doi: 10.1186/1471-2202-15-61 (PMC4027996; doi:10.1186/1471-2202-15-61)
Supplement: Additional file 3: Table S4 — Top 5 pathways from DAVID Functional Classification Analysis and Web-based Gene Set Analysis. [file 1471-2202-15-61-S3.doc]

Supplemental Table S4. Top 5 pathways from DAVID Functional Classification Analysis (top panel) and Web-based Gene Set Analysis (bottom panel), with DCM pathway highlighted and in italics.

| **Pathway name** | **KEGG ID** | ***P* value** |
| --- | --- | --- |
| ***Dilated cardiomyopathy*** | ***mmu05414*** | ***0.0089*** |
| Type II diabetes mellitus | mmu04930 | 0.0356 |
| Hypertrophic cardiomyopathy (HCM) | mmu05410 | 0.0635 |
| Focal adhesion | mmu04510 | 0.0669 |
| Leukocyte transendothelial migration | mmu04670 | 0.0841 |

| **Pathway name** | **KEGG ID** | ***P* value** |
| --- | --- | --- |
| MAPK signaling pathway | mmu04010 | 0.0005 |
| Type II diabetes mellitus | mmu04930 | 0.0032 |
| Long-term depression | mmu04730 | 0.0032 |
| ***Dilated cardiomyopathy*** | ***mmu05414*** | ***0.0039*** |
| Purine metabolism | mmu00230 | 0.0043 |
