# Supplementary material for: Chronic alcohol consumption from adolescence-to-adulthood in mice - hypothalamic gene expression changes in the dilated cardiomyopathy signaling pathway
Source: BMC Neurosci. 2014 May 9;15:61. doi: 10.1186/1471-2202-15-61 (PMC4027996; doi:10.1186/1471-2202-15-61)
Supplement: Additional file 4: Table S3 — Pooling of RNA samples from alcohol mice. [file 1471-2202-15-61-S4.doc]

Supplemental Table S3. Pooling of RNA samples from alcohol mice

| Pool name | Alcohol consumption status | Number of mice from | |
| --- | --- | --- | --- |
|  |  | 5% alcohol | 10% alcohol |
| A1 | Less alcohol consumption | 3 | 0 |
| A2 |  | 1 | 2 |
| A3 |  | 1 | 2 |
| A4 | More alcohol consumption | 2 | 0 |
| A5 |  | 2 | 0 |
| A6 |  | 0 | 2 |
| A7 |  | 1 | 0 |
| A8 |  | 0 | 1 |
| A9 |  | 0 | 2 |
