# Supplementary material for: Chronic alcohol consumption from adolescence-to-adulthood in mice - hypothalamic gene expression changes in the dilated cardiomyopathy signaling pathway
Source: BMC Neurosci. 2014 May 9;15:61. doi: 10.1186/1471-2202-15-61 (PMC4027996; doi:10.1186/1471-2202-15-61)
Supplement: Additional file 5: Table S2 — Sequences of primers for qRT-PCR. [file 1471-2202-15-61-S5.doc]

Supplemental Table S2. Sequences of primers for qRT-PCR

| **Symbol** | **Forward primer** | **Reverse primer** |
| --- | --- | --- |
| Adcy1 | CTTCTGTGGGCTCCTTGTTC | CACCTGGCTTAGCAGAGACC |
| Adrb1 | CGGCCTTTCGTGTGTTTAAT | CACACCAAACCTGAGCTGAA |
| Cacna1d | AATTCGGGGTGTCATAACCA | CGATCATGCTTGCAGGAGTA |
| Des | CAAAGGGGTTCTGAAGTCCA | GAAAAGTGGCTGGGTGTGAT |
| Gnas | GGAGAGTCTGGCAAAAGCAC | TGGGGTAGGACATAGCGAAG |
| Igf1 | TGCAAAGGAGAAGGAAAGGA | TGTTTTGCAGGTTGCTCAAG |
| Itga4 | CACAGCCACGGGTCGAA | AGGTCTGGTTTGGATTCTTTCTGA |
| Prkx | ATGCTGAGAACCAACCCAAC | GCACAACTCTCCCTGGACTC |
| Sgcd | GCCATGACCATCTGGATTCT | TCGGGACTTGATTTCTTTGG |
| Tnni3 | CTATGACCTCCGTGGCAAGT | CCTCCTTCTTCACCTGCTTG |
| β-actin | ACTGCCGCATCCTCTTCCT | AACCGCTCGTTGCCAATAG |
